# *Cpxm2* as a novel candidate for cardiac hypertrophy and failure in hypertension

**DOI:** 10.1038/s41440-021-00826-8

**Published:** 2021-12-16

**Authors:** Katja Grabowski, Laura Herlan, Anika Witten, Fatimunnisa Qadri, Andreas Eisenreich, Diana Lindner, Martin Schädlich, Angela Schulz, Jana Subrova, Ketaki Nitin Mhatre, Uwe Primessnig, Ralph Plehm, Sophie van Linthout, Felicitas Escher, Michael Bader, Monika Stoll, Dirk Westermann, Frank R. Heinzel, Reinhold Kreutz

**Affiliations:** 1 Charité—Universitätsmedizin Berlin, corporate member of Freie Universität Berlin, Humboldt-Universität zu Berlin, and Berlin Institute of Health (BIH), Institut für Klinische Pharmakologie und Toxikologie, 10178 Berlin, Germany; 2 Department of Genetic Epidemiology, Institute of Human Genetics, University Hospital Münster, Münster, Germany; 3 Max-Delbrück Center for Molecular Medicine (MDC), Berlin-Buch, Berlin, Germany; 4 German Center for Cardiovascular Research (DZHK), Partner site Hamburg/Kiel/Lübeck, Hamburg, Germany; 5 Clinic for Cardiology, University Heart and Vascular Center Hamburg, University Hospital Hamburg-Eppendorf, Hamburg, Germany; 6 Charité—Universitätsmedizin Berlin, corporate member of Freie Universität Berlin, Humboldt-Universität zu Berlin, and Berlin Institute of Health (BIH), Department of Cardiology, Campus Virchow Klinikum, 10178 Berlin, Germany; 7 German Center for Cardiovascular Research (DZHK), Partner Site Berlin, Berlin, Germany; 8 Charité—Universitätsmedizin Berlin, BCRT—Berlin Institute of Health Center for Regenerative Therapies, Berlin, Germany; 9 Institute of Cardiac Diagnostics and Therapy, IKDT GmbH, Berlin, Germany; 10 Charité—Universitätsmedizin Berlin, corporate member of Freie Universität Berlin, Humboldt-Universität zu Berlin, and Berlin Institute of Health (BIH), 10178 Berlin, Germany; 11 University of Lübeck, Institute for Biology, Ratzeburger Allee 160, 23562 Lübeck, Germany; 12 Department of Biochemistry, Cardiovascular Research Institute Maastricht, Maastricht University, Maastricht, The Netherlands

**Keywords:** Cardiac hypertrophy, Cpxm2, DOCA-salt hypertension, Genetics, Knock-out mice

## Abstract

Treatment of hypertension-mediated cardiac damage with left ventricular (LV) hypertrophy (LVH) and heart failure remains challenging. To identify novel targets, we performed comparative transcriptome analysis between genetic models derived from stroke-prone spontaneously hypertensive rats (SHRSP). Here, we identified carboxypeptidase X 2 (Cpxm2) as a genetic locus affecting LV mass. Analysis of isolated rat cardiomyocytes and cardiofibroblasts indicated Cpxm2 expression and intrinsic upregulation in genetic hypertension. Immunostaining indicated that CPXM2 associates with the t-tubule network of cardiomyocytes. The functional role of Cpxm2 was further investigated in Cpxm2-deficient (KO) and wild-type (WT) mice exposed to deoxycorticosterone acetate (DOCA). WT and KO animals developed severe and similar systolic hypertension in response to DOCA. WT mice developed severe LV damage, including increases in LV masses and diameters, impairment of LV systolic and diastolic function and reduced ejection fraction. These changes were significantly ameliorated or even normalized (i.e., ejection fraction) in KO-DOCA animals. LV transcriptome analysis showed a molecular cardiac hypertrophy/remodeling signature in WT but not KO mice with significant upregulation of 1234 transcripts, including Cpxm2, in response to DOCA. Analysis of endomyocardial biopsies from patients with cardiac hypertrophy indicated significant upregulation of CPXM2 expression. These data support further translational investigation of CPXM2.

## Abstract (web summary)

Differential increased cardiac expression of *Cpxm2* was assigned to cardiac hypertrophy in SHRSP rats. *Cpxm2* knock-out (KO) in mice reduced cardiac hypertrophy and remodeling in DOCA salt hypertension

## Graphical abstract

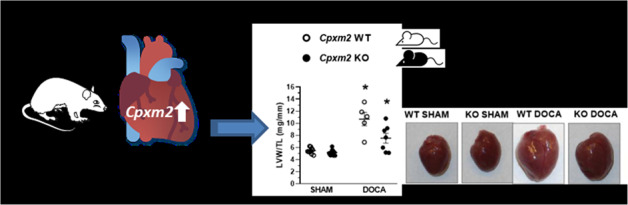

## Introduction

Hypertension-mediated organ damage (HMOD), including cardiac hypertrophy, has a major impact on the morbidity and mortality of hypertensive patients [1, 2]. Thus, sustained hypertension can result in maladaptive cardiac changes, including left ventricular (LV) structural remodeling with LV hypertrophy (LVH) and fibrosis, impaired (diastolic and systolic) LV function, left atrial enlargement and atrial fibrillation and, importantly, the development of heart failure with preserved ejection fraction (HFpEF) or reduced ejection fraction [2–4]. Approximately half of all patients presenting with HF have HFpEF [3], and hypertension is particularly prevalent in this condition [5], for which no established pharmacological therapies exist beyond blood pressure control [1, 2].

The development of LVH represents a pivotal early step in the pathophysiology of progression toward HF [3, 4], which is modulated by multiple individual and environmental factors, including genetic susceptibility variants that influence LV mass [6–8]. Animal models, including inbred hypertensive rat models [9], have proven to be an effective complementary tool for analyzing cardiovascular disease mechanisms, including the role of genetic factors in LV remodeling [10–14]. The stroke-prone spontaneously hypertensive rat (SHRSP) strain represents such a well-established inbred hypertension model with an inherited predisposition to developing HMOD [15, 16]. Regarding LV remodeling, Fischer (F344) rats are a suitable reference strain for the analysis of LVH because this strain features a contrasting phenotype with low LV mass and normal blood pressure levels [17]. Previous genetic studies in the SHRSP/F344 model confirmed the presence of a major quantitative trait locus (QTL) for LV mass on rat chromosome (RNO) 1 (Supplementary Fig. 1) [17]. Building on these previous findings, we set out to identify potential novel targets for LVH and LV remodeling in the SHRSP/F344 rat model. Our data support a functional role of CPXM2 as a novel candidate for LVH and adverse cardiac remodeling in hypertension.

## Methods

The authors declare that all supporting data are available within the article and in the Supplementary Material with an expanded Material and Methods section.

### Animal models

All animals were maintained under standard conditions of regular 12 h diurnal cycles using an automated light switching device and climate-controlled conditions at a constant room temperature of 22 °C. Animals had access to food and water ad libitum. All animal experiments were approved by the responsible local government committee and performed in accordance with the guidelines of the Charité-Universitätsmedizin Berlin and the local authority for animal protection (Landesamt für Gesundheit und Soziales, LaGeSo, Berlin, Germany). The registration numbers are G0354-13, T 0189-02, and O0052/03.

#### Rats

Male SHRSP and F344 rats were obtained from our colonies at the Forschungseinrichtung für Experimentelle Medizin (FEM), Charité—Universitätsmedizin, Berlin [17]. To develop the consomic strain SHRSP-1^F344^ (Rat Genome Database (RGD)-ID: SHRSP-Chr 1^F344^/Rkb), we crossed the SHRSP strain with the F344 strain in accordance with our linkage results and introgressed the whole rat chromosome (RNO)1 from F344 into the SHRSP background as previously described [17].

#### Mice

*Cpxm2* knock-out (KO) mice were obtained as heterozygous B6;129S5-*Cpxm2*^tm1Lex^/Mmucd/rk mice from the Mutant Mouse Regional Resource Center, University of California, Davis, USA, and were inbred to produce homozygous KO (*Cpxm2*^*−/−*^) and wild-type (WT) mice (*Cpxm2*^*+/+*^). Genotyping of the KO and WT alleles was performed by PCR (Supplementary Table 1). Only homozygous KO mice and corresponding WT mice were used in this study.

### DOCA-salt hypertension in Cpxm2 KO and WT mice - in vivo study

Baseline phenotypic characterization of all mice under control conditions was performed at ~12 weeks of age. Animals were subsequently followed for 8 weeks under either deoxycorticosterone acetate (DOCA) DOCA-salt or SHAM conditions.

### DOCA and SHAM experimental groups

At 12 weeks of age, unilateral nephrectomy was performed under anesthesia (isoflurane 3.5% induction followed by 1.5% maintenance dose) in WT and KO mice (*n* = 8/group), and a pellet containing DOCA was implanted in each mouse subcutaneously (DOCA pellet, 75 mg, 60-day release, Innovative Research of America, Sarasota, Florida, USA). Subsequently, these mice were exposed to salt by adding 1% NaCl to drinking water (DOCA groups). Unilateral nephrectomy was also performed in an additional group of WT and KO mice (*n* = 10/group), but these animals received no DOCA pellet and were given normal drinking water (SHAM groups). Animals were treated for 8 weeks overall.

### Radiotelemetry

Telemetry devices for continuous blood pressure and heart rate monitoring (TA11PA-C10, Data Sciences International, St. Paul, Minnesota, USA) were implanted in the DOCA mouse groups (WT DOCA *n* = 8, KO DOCA *n* = 8) under isoflurane (isoflurane 3.5% initially followed by 1.5%) anesthesia when the mice were 10 weeks of age [18]. The catheter was inserted into the right carotid artery, tunneled subcutaneously and placed in a subcutaneous pocket along the right flank. After a recovery period of 10 days, baseline recordings for blood pressure and heart rate were monitored for 3 days, and mice were subsequently followed with continuous 24 h blood pressure and heart rate monitoring for ~8 weeks under DOCA treatment.

### Analysis of cardiac and renal fibrosis and structural kidney damage

Hearts and kidneys were fixed in formalin (4%), embedded in paraffin and sectioned into 3 µm slices. Collagen was stained with picrosirius red for assessment of perivascular and interstitial fibrosis as reported [19]. The glomerulosclerosis index and tubulointerstitial damage index were determined as reported for the analysis of structural kidney damage [20].

### Isolation and analysis of primary cardiac cells

Cardiomyocytes were enzymatically isolated from adult SHRSP and F344 rats as previously described [21].

Rat primary cardiac fibroblasts were isolated via outgrowth culture as described for murine cardiac fibroblasts [22], with slight modifications. The outgrowing cardiac fibroblasts were cultured in Iscove’s medium (Biochrom, Berlin, Germany) containing 20% fetal calf serum (Biochrom), 100 U/ml penicillin and 100 µg/ml streptomycin (both PAA, Cölbe, Germany) at 37 °C with 95% air and 5% CO2.

### Expression analysis of human endomyocardial biopsies

Between 2003 and 2012, all patients with suspected cardiomyopathy were screened using clinical and endomyocardial biopsy-based diagnostics. The diagnosis of cardiac hypertrophy was made as recommended by the European Association of Cardiovascular Imaging and the American Society of Echocardiography [23]. In all patients, coronary artery disease and other possible causes of myocardial dysfunction were excluded by angiography and echocardiography before endomyocardial biopsy. All patients gave their written informed consent for data storage and evaluation. The study conformed to the principles outlined in the Declaration of Helsinki and was approved by the local institutional review committee of the University Clinic Benjamin Franklin, Berlin, Germany (approval 225-07). Patients’ data were anonymized for analyses.

Patients presenting with signs of acute myocarditis with recent onset of symptoms were excluded, as were those with proof of intramyocardial enterovirus, adenovirus, human herpesvirus 6, Epstein–Barr virus, or erythrovirus (B19V) genomes, as described previously [24]. Other exclusion criteria were a history of antiviral or immunosuppressive therapy, clinical or biochemical evidence of concomitant chronic inflammatory disease, inability to understand the consent form, or participation or consent to participate in another study. In addition, 16 patients without signs of congestive heart failure who underwent coronary angiography and endomyocardial biopsy to evaluate chest pain were also enrolled in this study and served as controls. Endomyocardial biopsies were obtained from the right ventricular septum. Out of the patients, we selected controls (*n* = 16 (38% male), median age 38.5 years, 95% CI 34.8–49.1) without any proven cardiac dysfunction and patients with hypertrophic cardiomyopathy or hypertrophic obstructive cardiomyopathy (*n* = 20 (75% male), median age 58 years, 95% CI 49.5–61.6).

Total RNA was isolated from endomyocardial biopsies during routine biopsy diagnostic analysis using TRIzol reagent (Life Technologies, Darmstadt, Germany), treated with DNase (PeqLab, Erlangen, Germany) to remove any traces of genomic DNA, and reverse-transcribed into cDNA using a High Capacity Kit (Life Technologies, Darmstadt, Germany) as described previously [25]. For quantitative real-time TaqMan PCR, 5 µl gene expression Mastermix (Thermo Fisher Scientific, USA) and 0.5 µl TaqMan gene expression assay purchased from Life Technologies; *CDNK1B*: Hs00153277_m1; *CPXM2*: Hs00406866_m1) were used in a final volume of 10 µl including 1 µl of cDNA template (the assays were performed in duplicate). Data were normalized to the *CDKN1B* mRNA level as an endogenous control and plotted as mRNA expression using the 2^−ΔCt^ method [26].

### Bioinformatic and statistical analysis

We assessed differences in gene expression between the three rat strains (SHRSP, F334, and SHRSP-1^F334^), and in the genome-wide transcriptome analysis, all samples except two samples (SHRSP and SHRSP-1^F334^) passed quality control and were subsequently analyzed (gene-level analysis with the R/Bioconductor packages systemsbio [27] and limma [28]; thresholds for differential expression: *p* < 0.05, Benjamini–Hochberg-corrected, and fold change > log2 (1.5)).

For genome-wide transcriptome analysis of the four experimental mouse groups (WT SHAM, KO SHAM, WT DOCA, and KO DOCA), microarray data were analyzed using TAC-console 4.0 from Affymetrix (parameters: analysis type “Expression (Gene)”, summarization: “Gene Level – RMA”, FDR corrected p value with fold change > 1, meta data loaded from “MoGene_2-0_st” array type). For mouse data, two outliers were identified and excluded from the analysis (one WT SHAM and one KO DOCA sample). Gene ontology (GO) enrichment analysis was performed on the transcriptome datasets as reported [29].

If not stated otherwise, data are presented as the means ± SEM. Differences between experimental groups were analyzed by Student’s *t* test, Mann–Whitney test, or one-way ANOVA with post hoc Bonferroni adjustments. A probability of *p* < 0.05 was considered to be statistically significant. We used IBM SPSS Statistics 22 for statistical analysis, GraphPad Prism and OriginLab 8.1 G for graph preparation (GraphPad Prism version 8.0 for Windows, GraphPad Software, La Jolla California USA, www.graphpad.com; OriginLab Corporation, Northampton, MA, USA).

## Results

### Transcriptome analysis in hypertensive rat models identifies *Cpxm2* as a novel molecule related to LVH

**Fig. 1:**
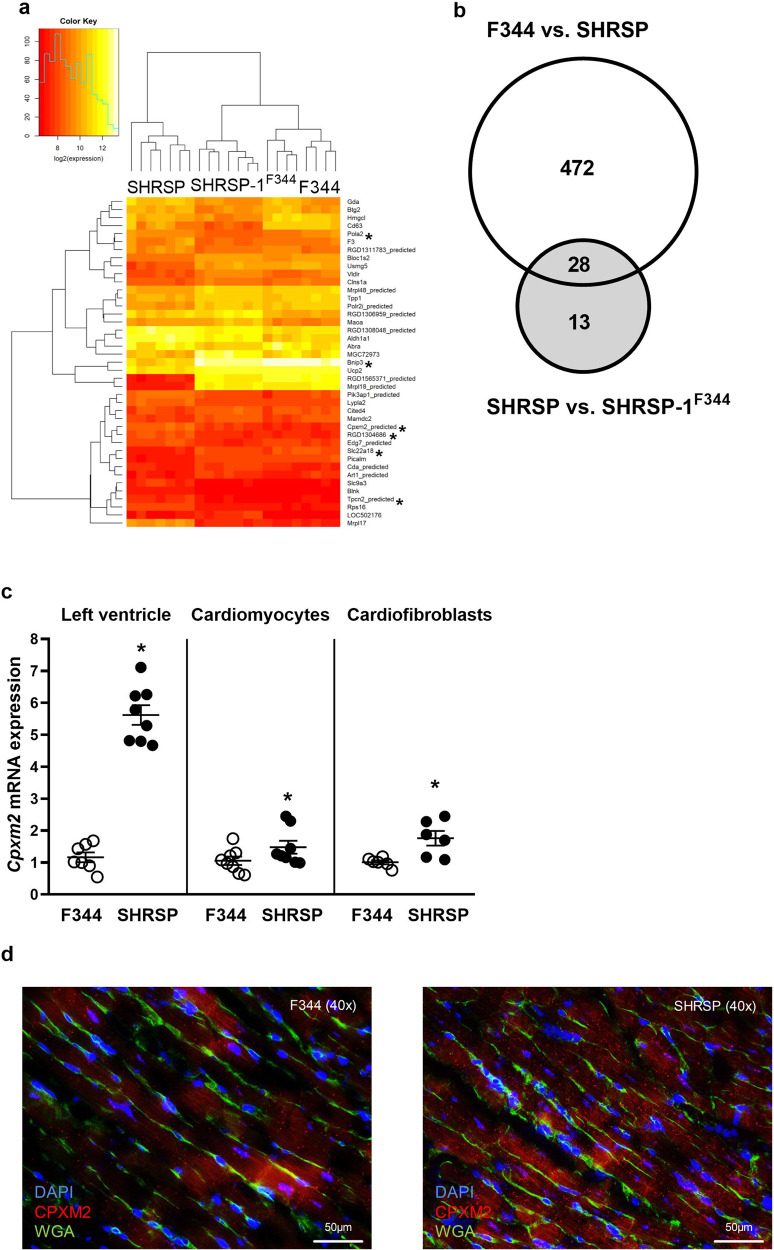
Detection of differential *Cpxm2* expression in rats. **a** Heatmap of differentially expressed transcripts between 14-week-old male SHRSP (*n* = 7), SHRSP-1^F344^ (*n* = 7) and F344 (*n* = 8) rats. Transcripts mapping within the LVH QTL interval between the genetic markers D1Rat60 and D1Rat71 are marked with an asterisk. A heatmap was generated using R 3.6.1 (R Core Team, 2019) and gplots were generated (Gregory R. Warnes, Ben Bolker, Lodewijk Bonebakker, Robert Gentleman, Wolfgang Huber, Andy Liaw, Thomas Lumley, Martin Maechler, Arni Magnusson, Steffen Moeller, Marc Schwartz and Bill Venables (2019)) using the Gplots package (Various R Programming Tools for Plotting Data, R, package version 3.0.1.1 https://CRAN.R-project.org/package=gplots). **b** Venn diagrams displaying the number of differentially expressed transcripts between the SHRSP and F344 (500 transcripts) and SHRSP and SHRSP-1^F344^ (41 transcripts) strains. Common transcripts in both comparisons are emphasized (*n* = 28). **c** *Cpxm2* mRNA expression (diagram) in left ventricular tissue, in primary isolated rat cardiomyocytes, and in primary isolated rat cardiofibroblasts derived from 14-week-old F344 (white) and SHRSP (black) rats (LV *Cpxm2* mRNA, *n* = 5–8). *Cpxm2* mRNA expression in rat cardiomyocytes and rat cardiofibroblasts is shown. At least three independent experiments were performed. Expression levels are displayed as fold changes of Cpxm2 expression compared to the expression in the F344 strain ± SEM. The rat mRNA expression of *Cpxm2* was normalized against Hprt expression. **d** Paraffin sections of hearts from the SHRSP (right section) and F344 (left section) strains were analyzed with immunofluorescent staining of CPXM2 (red, cy3), the cardiomyocyte cell wall (green, WGA Alexa488) and nuclei (blue, DAPI) (magnification ×40; scale bar: 50 µM). The corresponding whole LV sections are shown in Supplementary Fig. 9. Comparison of CPXM2 staining between the SHRSP and F344 strains indicated higher expression in the SHRSP strain. Statistical analyses were performed using Student’s *t* test in the rat experiments (*p < 0.05 vs. the F344 strain)

In LV transcriptome analysis, 541 transcripts were differentially expressed between SHRSP and the two reference strains (Supplementary Tables 2 and 3). To reduce model complexity, we subsequently focused on mRNAs that were differentially expressed between the SHRSP and consomic SHSRP-1^F344^ strains (Fig. 1a and Supplementary Table 3) because both strains share the same genetic background, while RNO1 carries the LVH QTL. In this analysis, 41 genes with differential expression were detected, mainly on RNO1 but also on rat chromosomes 2, 5, 7, 9, 13, 18 and X. Of these, 28 transcripts were also differentially expressed between the SHRSP and F344 strains as well as between the SHRSP and consomic SHSRP-1^F344^ strains (Fig. 1b and Tables S2 and 3). For further downstream studies, we selected only positional candidates, i.e., those transcripts mapping within the LVH QTL on RNO1 between genetic markers D1Rat60 and D1Rat71 (the chromosomal region between nucleotides 186,936,140 and 230,420,772; Fig. S2). Accordingly, six differentially expressed genes were identified, including Bnip3, Slc22a18, Cpxm2, Eif1ad, Tpcn2, and Pola2 (Fig. 1a, Tables S2 and 3 in bold). The gene expression of Slc22a18 and Bnip3 was lower in the SHRSP strain (fold change of 0.9 and 1.9, respectively), while the expression of Cpxm2 (fold change −0.9), Pola2 (fold change −0.9), Tpcn2 (fold change −0.8) and Eif1ad (fold change −0.8) was higher in the SHRSP strain than in the consomic strain. Slc22a18, Bnip3, Cpxm2, Tpcn2 and Eif1ad but not Pola2 were also differentially expressed between the SHRSP and F344 strain. Only Cpxm2 was confirmed in validation experiments because it demonstrated consistent and significant differential expression between the SHRSP and SHSRP-1^F344^ strains in separate qPCR expression analysis of LV tissue in adult and young rats at 4 and 8 weeks of age without established hypertension (Supplementary Fig. 3). Cpxm2 mRNA expression was significantly upregulated in LV tissue in the SHRSP strain in comparison to the F344 strain (at least a 2-fold difference in all age groups). GO enrichment analysis revealed no significant differential regulation of GO terms, i.e., biological process terms, between groups after correction for multiple tests.

### *Cpxm2* is expressed in both cardiomyocytes and cardiofibroblasts

Data mining of *Cpxm2* (gene ID: 293566 [rat], 55987 [mouse], and 119587 [human]) revealed its original classification as a carboxypeptidase, which was only predicted based on structural analysis [30]. Importantly, subsequent biochemical enzyme activity analysis demonstrated that CPXM2 lacks—despite its predicted classification—enzymatic carboxypeptidase function [30, 31]. Currently, its biological function remains unclear. We therefore determined the cardiac cell type(s) in which it is expressed by analyzing primary cardiomyocytes and cardiofibroblasts isolated and separated from LV cardiac tissue from the rat model. This analysis demonstrated that *Cpxm2* mRNA is expressed in both cardiomyocytes and cardiofibroblasts (Fig. 1c). In addition, the upregulation seen in total LV and heart paraffin tissue sections in the hypertensive model (Fig. 1c, d) was also confirmed for Cpxm2 mRNA expression in primary isolated cardiomyocytes and cardiofibroblasts obtained from the SHRSP strain compared to the F344 reference strain (Fig. 1c).

### Expression analysis of CPXM2 in rat cardiomyocytes

**Fig. 2:**
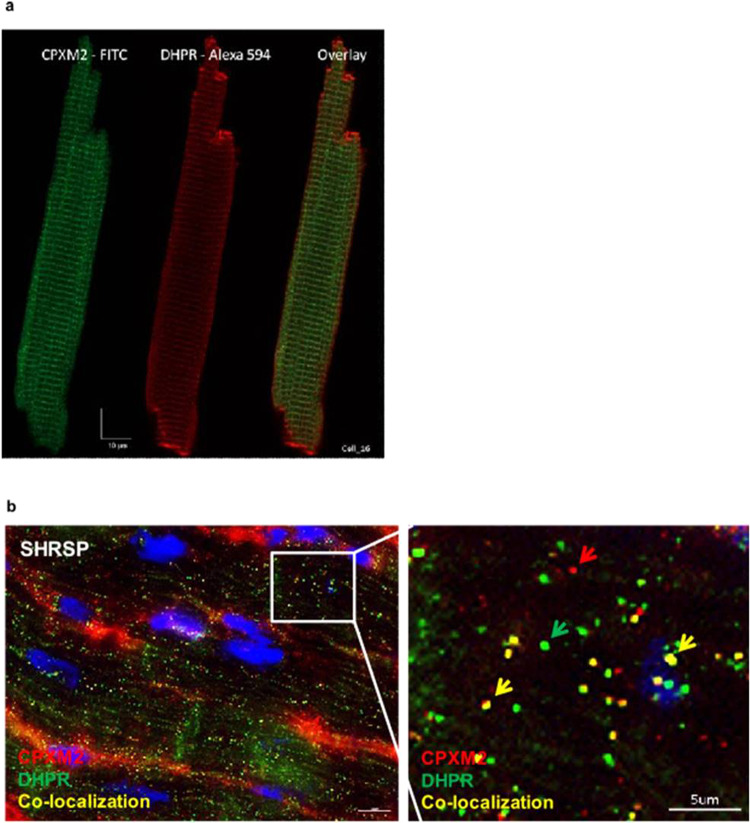
Cardiac fluorescence imaging analysis in rats. **a** Freshly isolated rat cardiomyocytes were analyzed using Z stack confocal fluorescence imaging. Cardiomyocytes were stained for CPXM2 (FITC, green) and L-type Ca2+ channel dihydropyridine receptor (DHPR) (Alexa 594, red), and corresponding overlays were obtained using a Leica SP5 confocal microscope (LeicaMicrosystems, Mannheim, Germany). Immunostaining was performed as described previously [63]. Image analysis after CPXM2 staining indicated expression along t-tubules but not in intercalated disks (Z-line), which was confirmed by corresponding DHPR staining. **b** Paraffin sections of SHRSP rats were analyzed for colocalization of CPXM2 (red arrow) and DHRP (green arrow). Colocalization is marked with yellow arrows. Scale bar: 5 µM

Confocal fluorescence image analysis of isolated SHRSP rat cardiomyocytes indicated that CPXM2 was expressed along the t-tubule network and colocalized with dihydropyridine receptor (DHPR) (Fig. 2a). This was also confirmed by immunostaining of paraffin sections of rat hearts (Fig. 2b), which also indicated expression in the noncardiomyocyte compartment.

### In vivo evaluation of *Cpxm2* effect on cardiac damage in *Cpxm2*-deficient mice with DOCA-salt hypertension

#### Body, heart, and kidney weight

**Fig. 3:**
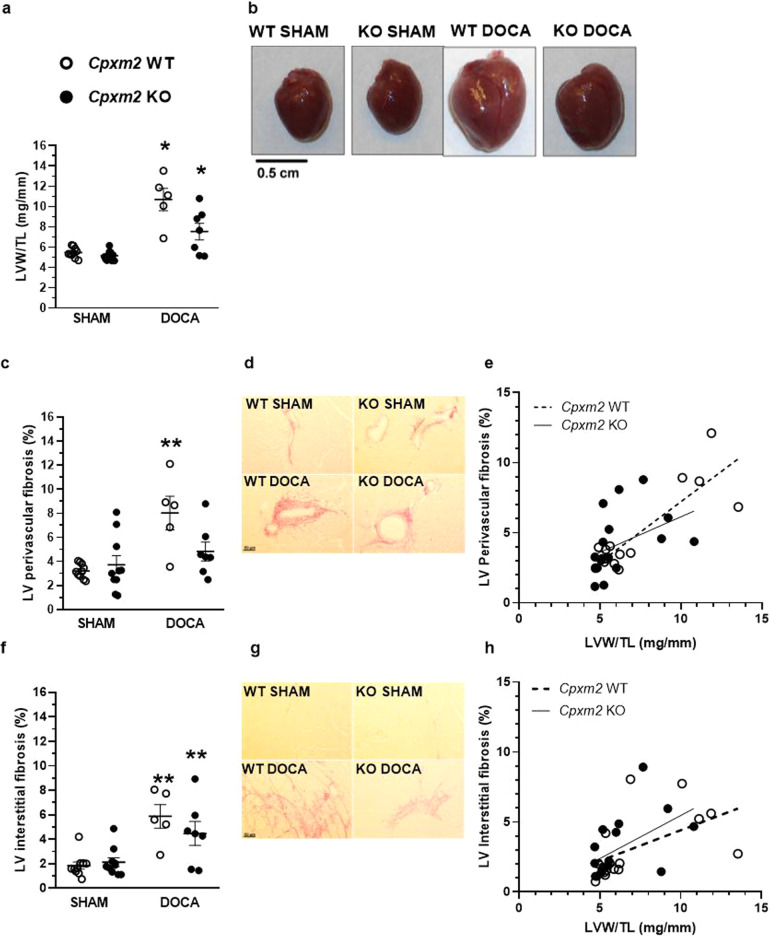
Cardiac structural and molecular characteristics of *Cpxm2* wild-type (WT) and knock-out (KO) mice under SHAM and DOCA conditions. The left ventricular (LV) weight and LV fibrosis rate as well as genetic expression profile in WT (white) and KO (black) after 8 weeks of SHAM or DOCA treatment, *n* = 3–10. **a** Mean relative left ventricular weight [LVW/tibia length (TL)]. **b** Example whole hearts. **c**–**e** Mean perivascular fibrosis was analyzed after picrosirius red staining in three fields of view per animal; representative photographs are shown (**d**). Mean perivascular fibrosis was plotted against relative LV weight for *Cpxm2* WT (*r* = 0.857, *p* = 0.00004, **e**) and *Cpxm2* KO mice (*r* = 0.417, *p* = 0.096, **e**). **f**–**h** Mean interstitial fibrosis was analyzed after picrosirius red staining in three fields of view per animal; representative photographs are shown (**g**). Mean interstitial fibrosis was plotted against relative LV weight for *Cpxm2* WT (*r* = 0.531, *p* = 0.04, **h**) and *Cpxm2* KO (*r* = 0.525, *p* = 0.03, **h**). Statistical analyses were performed using one-way ANOVA with the Bonferroni post hoc test, *p < 0.05 vs. all other groups, **p < 0.05 vs. SHAM

Untreated KO mice did not demonstrate any morphological or behavioral differences in comparison with WT animals under normal conditions. In response to DOCA or SHAM treatment, no significant group differences in body weight were observed at the end of the observation period (after 8 weeks) (Table 1). Total heart weight was significantly increased in DOCA-treated mice compared to the corresponding SHAM mice (Table 1). Relative LV weight was markedly increased in WT DOCA compared to WT SHAM mice (+95%, Fig. 3a, b, *p* = 0.000002), and this increase was significantly lower in the KO DOCA group than in the KO SHAM group (+46%, Fig. 3a, b, *p* = 0.01) compared to the WT group. Relative kidney weights were also increased in DOCA-treated animals but without group differences (Table 1).

**Table 1:** Characteristics of WT SHAM, WT DOCA, KO SHAM and KO DOCA animals after 8 weeks of DOCA treatment

Phenotype	Strain	SHAM	DOCA	*p*-value (ANOVA)				
				Overall	WT SHAM vs. WT DOCA	KO SHAM vs. KO DOCA	WT SHAM vs. KO SHAM	WT DOCA vs. KO DOCA
BW (g)	WT	31.62 ± 0.99	29.19 ± 0.94	**0.040**	0.387	0.237	1.000	1.000
	KO	32.70 ± 0.54	30.24 ± 0.63					
HW (mg)	WT	125.40 ± 3.63	225.38 ± 23.31	**0.000001**	**0.000006**	**0.014**	1.000	**0.011**
	KO	118.06 ± 2.41	166.30 ± 16.58					
KW (mg)	WT	222.90 ± 7.03	277.80 ± 15.30	**0.00002**	**0.044**	**0.00006**	1.000	0.643
	KO	220.30 ± 5.62	311.50 ± 22.60					
KW/TL (mg/mm)	WT	12.31 ± 0.36	15.52 ± 0.85	**0.00003**	**0.037**	**0.0001**	1.000	1.000
	KO	12.05 ± 0.35	17.08 ± 1.32					
SV (µl)	WT	28.78 ± 1.46	31.67 ± 4.72	0.198	1.000	0.359	1.000	1.000
	KO	28.83 ± 1.86	35.72 ± 3.35					
CO (ml/min)	WT	13.33 ± 0.86	14.10 ± 2.32	0.598	1.000	1.000	1.000	1.000
	KO	12.29 ± 1.03	14.62 ± 1.54					
FS (%)	WT	28.10 ± 1.77	12.47 ± 1.01	**0.00008**	**0.00003**	1.000	0.391	**0.005**
	KO	23.74 ± 1.31	23.58 ± 2.52					

Phenotype values are means ± SEM. Significant *p*-values are presented in bold

*BW* body weight, *HW* heart weight, *KW* kidney weight, *TL* tibia length, *SV* stroke volume, *CO* cardiac output, *FS* fractional shortening, *WT* wild type, *KO* knock-out, *SHAM* SHAM-operated, *DOCA* Desoxycorticosterone acetate-treated

#### LV remodeling analysis

Histological analyses of cardiac tissue revealed a significant increase in LV perivascular and interstitial fibrosis (Fig. 3c–h) in WT DOCA mice vs. WT SHAM mice (*p* = 0.01 vs. SHAM groups). The increase in LV perivascular fibrosis observed in WT DOCA mice was not observed in KO DOCA mice (Fig. 3c), while LV interstitial fibrosis, although quantified as lower than that in WT DOCA mice, was still significantly elevated in KO DOCA mice compared to SHAM mice (Fig. 3f, *p* = 0.048). Collectively, an overall significant positive correlation between LV fibrosis and relative LV weight was found in all groups (perivascular fibrosis *r* = 0.702, *p* = 0.000008; interstitial fibrosis *r* = 0.513, *p* = 0.003). However, while further analysis confirmed the significant positive correlation between relative LV weight and perivascular fibrosis (*p* = 0.0004, Fig. 3e) and interstitial fibrosis in WT mice (*p* = 0.04, Fig. 3h), KO mice exhibited a significant correlation only between relative LV weight and interstitial fibrosis (*p* = 0.03, Fig. 3h).

#### Blood pressure and heart rate

**Fig. 4:**
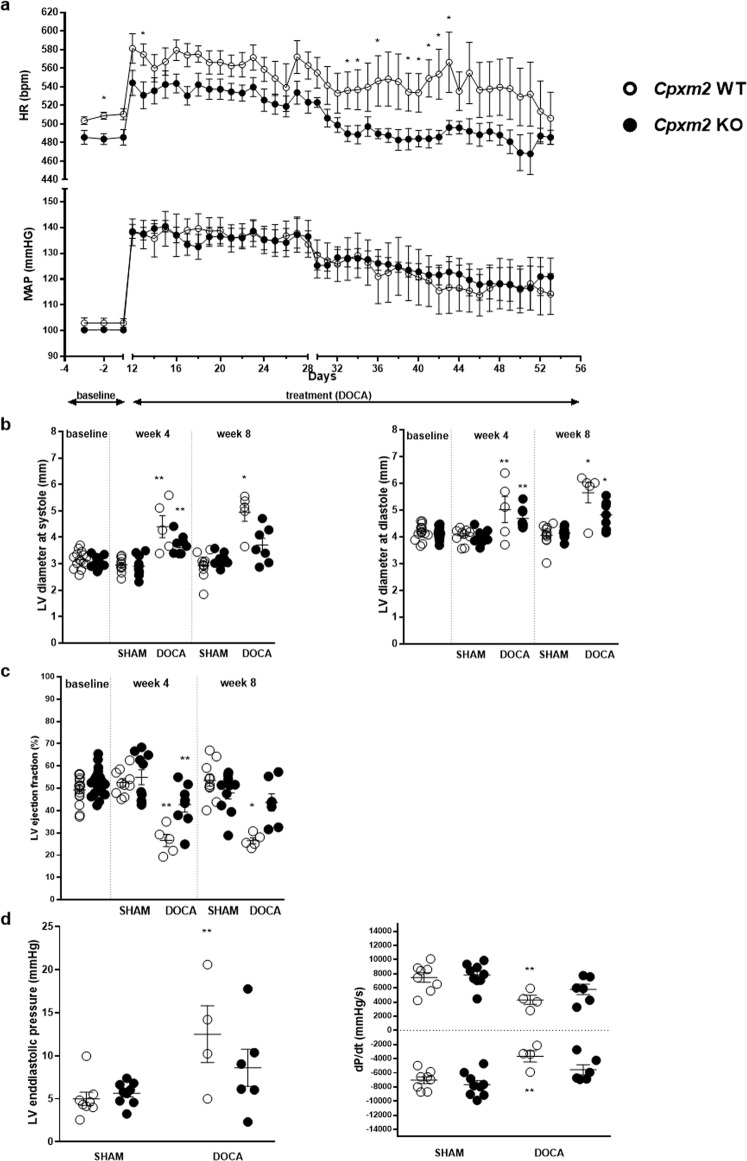
Cardiac function characteristics of *Cpxm2* wild-type (WT) and knock-out (KO) mice under SHAM and DOCA conditions. **a** Mean arterial blood pressure (MAP) and heart rate (HR) in the DOCA mouse model. MAP (bottom) and HR (top) in WT (white, *n* = 4–7) and KO (black, *n* = 7–8) mice before DOCA pellet implantation and unilateral nephrectomy (baseline, Days −3 to −1) and after DOCA pellet implantation and unilateral nephrectomy (DOCA treatment, Day 12 to Day 53). MAP and HR are displayed as the mean ± SEM; statistical analyses were performed using the Mann–Whitney test, *p < 0.05 vs. WT DOCA (Days −2, 13, 33, 34, 36, and 39–43). **b**, **c** Left ventricular (LV) function according to echocardiographic analysis of WT (white) and KO (black) mice under SHAM and DOCA conditions after baseline (before DOCA pellet implantation and unilateral nephrectomy), after 4 weeks of DOCA treatment and after 8 weeks of DOCA treatment; *n* = 5–10. Mean LV diameter at systole (**b**, left diagram) and diastole (**b**, right diagram) and mean LV ejection fraction (EF, **c**) are displayed. **d** LV function according to hemodynamic analysis of WT (white) and KO (black) mice after 8 weeks of SHAM or DOCA treatment, *n* = 5–10. The mean LV end-diastolic pressure (left diagram) and mean maximum and minimum values of the pressure derivative during a loop (dP/dt, right diagram) are displayed. Statistical analyses were performed using Student’s *t* test for the baseline data and one-way ANOVA with the Bonferroni post hoc test for data from 4 weeks and 8 weeks, *p < 0.05 vs. all other groups at the same time point, **p < 0.05 vs. the corresponding SHAM group

Telemetric analysis in DOCA group animals at baseline, i.e., before DOCA and salt exposure, revealed no significant blood pressure difference between WT and KO mice (Fig. 4a). After uninephrectomy and DOCA implantation (Day 0), the mean arterial pressure (MAP) increased significantly by almost 40 mmHg in both strains (KO *p* = 0.000004 and WT *p* = 0.001, Fig. 4a). After 4 weeks, MAP decreased continuously during the observation period but did not differ between KO and WT DOCA mice. Heart rate (HR) was slightly but significantly lower at baseline in KO animals than in WT animals (−23 bpm at day −2, *p* = 0.009, Fig. 4a). During the first 4 weeks in which DOCA-salt-induced hypertension was present, HR increased significantly in both strains (KO *p* = 0.004 and WT *p* = 0.0004), which showed no significant difference, although HR remained numerically lower in KO DOCA mice. Subsequently, between weeks 4 and 8 of DOCA exposure, between Days 33 and 43, HR was significantly lower in KO DOCA mice than in WT DOCA mice (Day 33 *p* = 0.045, Day 34 *p* = 0.045, Day 36 *p* = 0.048, Day 39 *p* = 0.048, Day 40 *p* = 0.048, Day 41 *p* = 0.016, Day 42 *p* = 0.016, Day 43 *p* = 0.028; Fig. 4a).

#### LV functional analysis

Echocardiography time course analysis demonstrated a significant and progressive enlargement of end-systolic (after 8 weeks + 69%, *p* = 0.0000006) and end-diastolic (after 8 weeks + 40%, *p* = 0.00001) LV internal diameters in DOCA-treated WT mice compared to SHAM WT mice (Fig. 4b). This LV enlargement was significantly attenuated in DOCA KO mice (end-systolic LV internal diameter *p* = 0.002 and end-diastolic LV internal diameter *p* = 0.04 vs. DOCA WT mice, respectively). WT DOCA mice showed significantly reduced LV ejection fraction (EF) after 4 and 8 weeks of DOCA treatment compared to WT SHAM mice (Fig. 4c, after 4 weeks *p* = 0.00003 and after 8 weeks *p* = 0.00001, respectively). This decline in EF was significantly attenuated in DOCA-treated KO mice (*p* = 0.01 vs. DOCA WT mice). Of interest, at 8 weeks of DOCA treatment, LV internal systolic diameter and EF were not significantly different between the DOCA KO groups and SHAM groups (Fig. 4b, c). Similarly, fractional shortening (FS) was significantly reduced in WT DOCA mice (after 4 weeks *p* = 0.000005 and after 8 weeks *p* = 0.00003, Table 1) but not in KO DOCA mice compared to the corresponding SHAM mice (Table 1).

Hemodynamic characterization at the end of the observation period by LV pressure-volume catheter analysis revealed that LV end-systolic volume (LVESV, +600%, *p* = 0.0000000001) and end-diastolic volume (LVEDV, + 146%, *p* = 0.00000004) were significantly elevated in the WT DOCA group compared with the SHAM group, in agreement with the echocardiography data. LVESV and LVEDV were significantly lower (−61% and −51%) in KO DOCA mice than in WT DOCA mice (LVESV *p* = 0.0000001 and LVEDV *p* = 0.00006, respectively). LV end-diastolic pressure (LVEDP) was significantly elevated in WT DOCA but not in KO DOCA mice compared to SHAM mice (*p* = 0.02, Fig. 4d). In addition, both systolic (+dP/dt) and diastolic (−dP/dt) LV function were significantly impaired (*p* = 0.04 and *p* = 0.01, respectively) in WT DOCA but not in KO DOCA mice compared to SHAM mice (Fig. 4d).

When we analyzed the correlation between individual Cpxm2 mRNA expression and cardiac phenotypes in WT mice following DOCA treatment, a nonsignificant (possible due to the limited sample size) negative trend for correlation between the Cpxm2 mRNA level and relative LV weight (*r* = −0.872, *p* = 0.054) and the Nppa mRNA expression level (*r* = −0.381, *p* = 0.527) was observed, while the correlation with LV EF was blunted (*r* = −0.003, *p* = 0.996) due to the low level of variation of this phenotype in all WT DOCA mice (Supplementary Fig. 4).

#### Renal damage analysis

Analysis of structural glomerular and tubulointerstitial damage indices revealed a significant increase in both DOCA groups but no significant difference between WT and KO mice (Supplementary Fig. 5b, c). Renal interstitial fibrosis was also numerically higher in both DOCA groups, although this increase in KO DOCA compared with KO SHAM mice was not significant (Supplementary Fig. 5a).

#### Cardiac transcriptome analysis

**Fig. 5:**
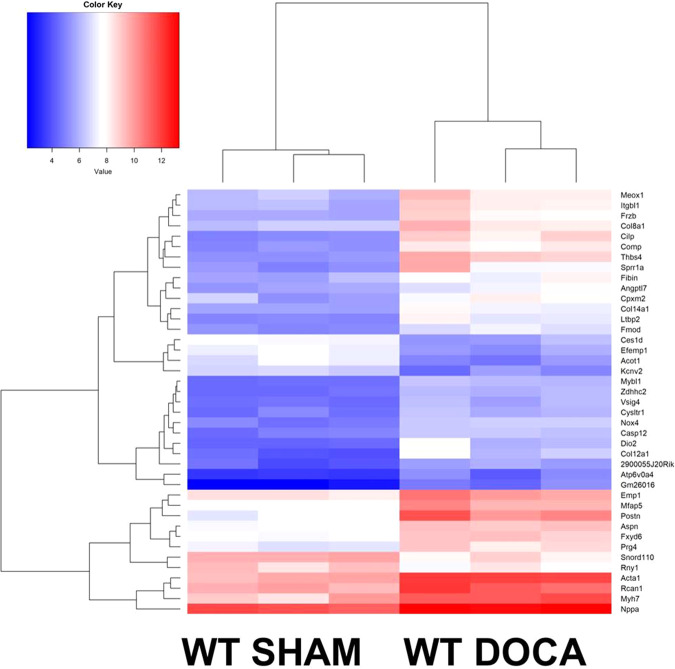
Cardiac molecular characteristics of *Cpxm2* wild-type (WT) and knock-out (KO) mice under SHAM and DOCA conditions. Heatmap of differentially expressed transcripts between 8-week-old WT SHAM mice (*n* = 3) and WT DOCA mice (*n* = 3). The top 41 differentially expressed transcripts are displayed. A heatmap was generated using R 3.6.1 (R Core Team, 2019) and the gplots package (Gregory R. Warnes, Ben Bolker, Lodewijk Bonebakker, Robert Gentleman, Wolfgang Huber, Andy Liaw, Thomas Lumley, Martin Maechler, Arni Magnusson, Steffen Moeller, Marc Schwartz and Bill Venables (2019); Various R Programming Tools for Plotting Data. R, package version 3.0.1.1 https://CRAN.R-project.org/package=gplots)

Under SHAM conditions, no significant differences in LV transcriptome analysis data were observed between WT and KO mice. In contrast, 1243 transcripts were differentially expressed between WT SHAM and WT DOCA mice (Fig. 5 and Supplementary Table 4); a typical cardiac hypertrophy and remodeling expression pattern, e.g., significant upregulation of actin alpha 1 (Acta 1, −4.8-fold change), Nppa (−3.2-fold change), and periostin (Postn −7.9-fold change), was observed in DOCA-treated WT mice (Supplementary Table 4). Of interest, there were no differentially expressed transcripts between the KO SHAM and KO DOCA mice in the transcriptome analysis, underlining the impact of Cpxm2 on cardiac remodeling. In the separate qPCR analysis, we confirmed the significantly increased mRNA expression of natriuretic peptide A (Nppa) in WT DOCA compared to WT SHAM mice (FDR *p* = 0.0000003, Supplementary Fig. 5), while no significant increase was seen in KO DOCA compared to KO SHAM mice (FDR *p* = 0.79, Supplementary Fig. 6). However, Nppa mRNA expression was also significantly higher in WT DOCA than in KO DOCA mice (*p* < 0.05, Supplementary Fig. 6) in the targeted separate mRNA expression analysis by qPCR. Interestingly, Cpxm2 was one of the most differentially expressed transcripts between the two WT groups (fold change −4.9, FDR *p* = 0.02, Supplementary Table 4) and was upregulated in DOCA-treated WT mice; the latter finding was confirmed in separate qPCR analysis, while KO mice showed no LV Cpxm2 mRNA expression (Supplementary Fig. 7). In the direct comparison between WT DOCA and KO DOCA mice, only one transcript, encoding the gene Zdhhc2, was significantly upregulated after correction for multiple tests (−2.71-fold change, FDR *p* = 0.04) in WT DOCA mice. This transcript was also significantly upregulated in WT DOCA mice compared to WT SHAM mice (−3.12-fold change, FDR *p* = 0.005). GO enrichment analysis indicated a significant association of GO terms with the response to DOCA in WT mice but not in KO mice, including the terms response to hypoxia, fatty acid beta-oxidation, heart development, cell adhesion, and response to mechanical stimulus (FDR *p* < 0.03).

#### CPXM2 expression is upregulated in patients with cardiac hypertrophy

**Fig. 6:**
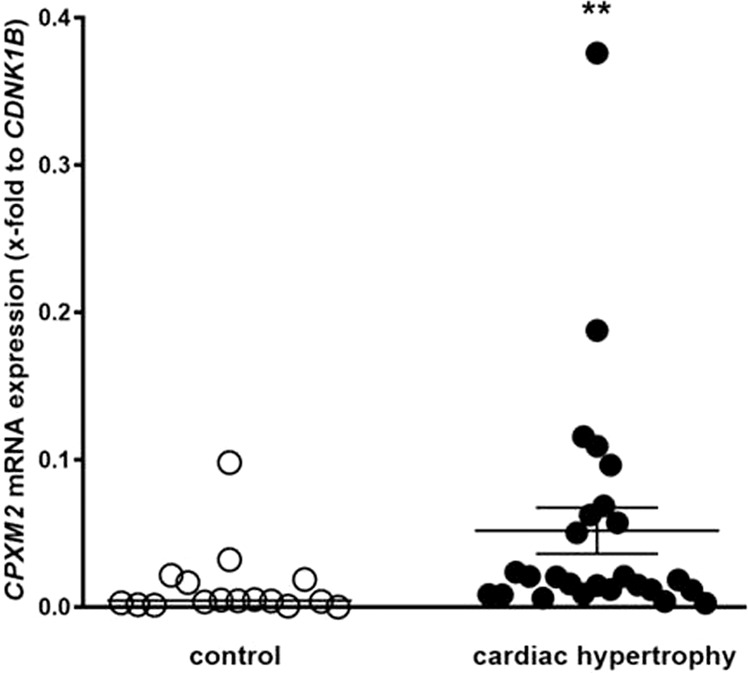
Detection of differential *Cpxm2* expression in humans.*CPXM2* mRNA expression in control patients without cardiac hypertrophy (*n* = 16) and in patients with cardiac hypertrophy (*n* = 20). The human mRNA expression of *CPXM2* was normalized against *CDKN1B* expression. Statistical analyses were performed using the Mann–Whitney test in human samples, **p < 0.01 vs. control

We performed explorative analysis of CPXM2 mRNA expression in samples isolated from endomyocardial biopsies of human patients with cardiac hypertrophy and controls. CPXM2 mRNA expression was significantly higher in patients with cardiac hypertrophy (*n* = 20) than in patients without cardiac hypertrophy (*n* = 16, *p* = 0.0054, Fig. 6).

## Discussion

In this study, we first performed comparative transcriptome analysis of LV tissues of rat models of hypertension, which mapped a novel candidate, i.e., *Cpxm2*, to a previously identified QTL for LV mass [17]. In subsequent validation experiments, we confirmed the expression and upregulation of Cpxm2 in primary cardiac fibroblasts, cardiomyocytes and total LV tissue in SHRSP rats.

The similar (in vivo) upregulation of Cpxm2 in the hearts of young animals at 4 weeks of age and before the development of sustained hypertension and in (in vitro) isolated cardiomyocytes and cardiofibroblasts corroborated a potential intrinsic role of Cpxm2 in LV remodeling in hypertension. In aging SHRSP rats, there was a subsequent further pronounced increase in Cpxm2 expression, which paralleled the reported increase in blood pressure in this strain (Fig. S3), suggesting additional upregulation in response to pressure overload. Nevertheless, other systemic effects, e.g., neurohumoral activation and cardiac infiltration of inflammatory cells, could also contribute to the further increase in Cpxm2 expression in the SHRSP strain. In a previous study, we clearly demonstrated that LV tissue levels of angiotensin II were not elevated in the SHRSP strain that we used in the current work [32]. Consequently, it appears that the upregulation of Cpxm2 expression in the SHRSP strain is not primarily induced by angiotensin II. This notion does not, however, exclude the possibility that inhibition of angiotensin II or aldosterone signaling with respective drug treatments can prevent the induction of Cpxm2 expression in the heart. Such strategies could be explored in future studies. Our subsequent studies in *Cpxm2-*deficient mice demonstrated a significant impact on maladaptive remodeling and heart failure development in DOCA-salt hypertension. DOCA-treated Cpxm2 KO mice showed attenuated LV dysfunction and enlargement as well as decreased LV fibrosis compared with DOCA-treated Cpxm2-WT mice [33]. In contrast, Cpxm2 deficiency had no protective effect on structural tissue damage in the kidney. The development of hypertension (mild to severe) and cardiac remodeling (diastolic to systolic dysfunction) in response to DOCA exposure may vary depending on the DOCA dose and time course of exposure, differences between species and strains, and combination with unilateral nephrectomy and increased dietary salt intake [34–37]. The DOCA protocol used in the current study in mice clearly induced severe hypertension and resulted in marked increases in LV masses and diameters as well as impairment of both diastolic and systolic LV function in WT mice. The lack of *Cpxm2* in KO mice significantly ameliorated this phenotype.

In the comparison between WT and KO mice, a significant, albeit modest, lower HR was noted at baseline in KO animals. This difference and the subsequent trend of a lower HR in Cpxm2-deficient mice might have contributed to the protective cardiac effect in response to DOCA. The lower HR in KO animals could be attributable either to intrinsic cardiac effects of Cpxm2 deficiency or to systemic effects, possibly related to the ability of the autonomic nervous system to control heart rate, in the general KO model. Future studies of conditional KO models could help dissect these effects; however, such studies would require targeting both cardiomyocytes and cardiofibroblasts based on the observed cardiac expression of Cpxm2.

Importantly, transcriptome analysis demonstrated a profound impact of *Cpxm2* KO on the LV transcriptome in response to DOCA hypertension. Hence, the induction of a characteristic LVH/remodeling transcriptome signature with a number of significant changes in response to DOCA in WT mice was abolished overall in *Cpxm2*-deficient DOCA mice. This result is in line with the moderate structural and functional cardiac remodeling phenotypes observed in the KO DOCA mice. However, a limitation of our microarray analysis is the limited sample size, which may have resulted in low statistical power for detecting differentially expressed genes (Supplementary Fig. 8).

Of interest, *Cpxm2* mRNA expression was also significantly upregulated in LV tissue of WT DOCA mice compared to control WT mice.

Our expression analysis of CPXM2 in endomyocardial biopsies of patients confirmed not only the expression of CPXM2 in the human heart but also significant CPXM2 upregulation in patients with various forms of cardiac hypertrophy. Our analysis of Cpxm2 expression in biopsies of patients should be considered explorative and for the purpose of generating hypotheses. Notwithstanding these limitations, it is important to confirm the expression of Cpxm2 in the human heart and to provide preliminary support for the differential regulation of CPXM2 in human heart diseases, which would provide a rationale for further clinical studies.

Carboxypeptidases by definition are enzymes with catalytic activity that remove protein C-terminal amino acids; they show diverse tissue distribution and biological functions [38, 39]. However, at least three members of the carboxypeptidase N/E subfamily lack catalytic function [31]: (i) carboxypeptidase × member 1 (CPXM1, CPX1, CPXM); (ii) carboxypeptidase × member 2 (CPXM2, CPX2); (iii) and aortic carboxypeptidase-like protein/adipocyte enhancer binding protein 1 (ACLP, alias AE binding protein AEBP1). Thus, it is important to emphasize that CPXM2 also lacks enzymatic carboxypeptidase function for typical substrates [30, 31]. Currently, its biological function remains unclear. Moreover, the presence and potential function of CPXM2 in the circulation remain to be determined. CPXM1 was demonstrated to be involved in adipogenesis, osteoclastogenesis, and cancer [40–42]. Earlier studies showed AEBP1 to have a functional role in fibroblast organization and transition, wound healing, and neuromuscular development [43, 44]. More recently, AEBP1 was identified as a candidate gene in Ehlers-Danlos syndrome [45]. Previous studies have indicated a role of Cpxm2 in oncogenesis and development, as well as in disorders [46–50]. Interestingly, a mouse model with caloric restriction demonstrated reduced Cpxm2 expression in whole hearts [51]. These three proteins all contain an N-terminal domain with homology to the discoidin domain, i.e., discoidin 1, whereas none of the other members in the metallocarboxypeptidase family contain such a domain [31]. Thus, the potential interacting structure of Cpxm2 may lie in its discoidin or factor 5/8 type C domain [52]. These domains can bind to lipids, glycans, heparin-like molecules and proteins (reviewed in [52]). Proteins containing discoidin domains have been found to be related to cellular adhesion, migration, and aggregation events. These relationships could be associated with extracellular matrix function and thus influence cardiac remodeling [53–55]. The expression of CPXM2 in cardiofibroblasts appears to be in agreement with existing knowledge of discoidin domain-containing proteins and their role in extracellular remodeling. In contrast, the expression of CPXM2 in cardiomyocytes and its potential relevance are currently unclear. Nevertheless, GO enrichment analysis of the transcriptome dataset comparing the response to DOCA of the WT and KO mice indicated a significant association with the GO terms response to hypoxia, fatty acid beta-oxidation, heart development, cell adhesion, and response to mechanical stimulus. These biological processes are linked to cardiac function, cardiac remodeling and cardiac failure, and their associations thereby substantiate a putative role of CPXM2 in cardiac failure. Furthermore, our immunostaining analysis revealed that expression of CPXM2 in cardiomyocytes was associated with the t-tubule network and colocalized with DHPR. Of interest, a recent systematic analysis of the proteome of cardiac t-tubules indicated that a few proteins (1.8%) of all extracellular (matrisome) proteins of the heart had a tubule-staining immunohistochemistry pattern [56]. In this regard, Cpxm2 may represent a novel member of this small group of proteins that maintain the structure and function of t-tubules by being expressed in both compartments [56, 57]. Cpxm2 could link mechanical signals that act on the extracellular matrix and cardiomyocyte cell membranes of the t-tubule network and may play an important role in cardiac tissue remodeling in hypertension, as recently reviewed by Saucerman et al. [57].

The identified localization of the Cpxm2 protein at t-tubules indicates its potential role in the homeostatic function of the t-tubule structure or calcium handling, which should be investigated in future functional studies on calcium-handling proteins. Although limited in sample size, our microarray transcriptome analysis revealed no significant differences in mRNA expression levels between WT and Cpxm2-deficient mice during SHAM conditions for genes involved in calcium handling, including Atp2a2 (SR Ca2+ -ATPase [(SERCA2], Cav3 (cavelolin 3), Ncx (sodium/calcium exchanger), Ryr2 (ryanodine receptor 2), Pln (phosholamban) and Sln (sarcolipin).

Our LV transcriptome analysis in the mouse model indicated that only Zdhhc2 was differentially expressed between WT DOCA and KO DOCA mice after correction for multiple tests, while for example, Nppa mRNA also showed significantly higher levels in WT DOCA mice in the separate targeted analysis comparing WT DOCA and KO DOCA mice using qPCR. This result points to a potential link between Cpxm2 and the enzymes of the zinc-finger aspartate-histidine-histidine-cysteine family (zDHHC enzymes). These enzymes mediate S-acylation (palmitoylation) and are thus important in posttranslational modification [58]. Enzymatic S-acylation regulates the localization and function of different intracellular and membrane proteins [58], including cardiac ion channels and transporters, e.g., Nav1.5 [59]. The impact of altered palmitoylation of NAV1.5 on cardiac excitability was previously demonstrated in vitro [59]. However, thus far only Carboxypeptidase D, a carboxypeptidase with enzymatic function, was identified as a substrate of S-palmitoylation, and this S-palmitoylation impacts its cellular localization and half-life [60]. Further studies are thus necessary to clarify the potential interaction of Cpxm2 and Zdhhc2.

Another limitation of our study is related to the fact that for the definitive confirmation of a causative effect of Cpxm2 on the development of LVH in SHRSP rats, targeted knock-out of this gene in SHRSP rats is warranted [61]. Moreover, experiments in transgenic animals overexpressing Cpxm2 in the heart could also provide additional important information.

In summary, novel proteins that are expressed in the left ventricle of the heart and contribute to the transition from LVH to heart failure are of interest for the prevention and treatment of heart failure [62]. *Cpxm2* represents such a novel target, as it is upregulated in genetically hypertensive rat models, in response to DOCA-salt hypertension in mice, and in human patients with cardiac hypertrophy. *Cpxm2* deficiency in mice with sustained hypertension results in profound protection against cardiac damage and failure. Although CPXM2 lacks enzymatic function and its molecular function is currently unclear, its expression and upregulation in both cardiomyocytes and cardiofibroblasts appears to be important. Moreover, its assignment to the small group of proteins that are expressed in both the matrisome and the t-tubule network of cardiomyocytes suggests that CPXM2 could play a role at this unique interface and possibly be involved in mechanosensitive pathways in the heart.

## Supplementary information

Supplementary Information

Supplementary Figures

Supplementary Table 2

Supplementary Table 3

Supplementary Table 4

Supplementary image

## Data Availability

The datasets generated and/or analyzed during the current study are available from the corresponding author on reasonable request.
