# Supplementary material for: Cpxm2 as a novel candidate for cardiac hypertrophy and failure in hypertension
Source: Hypertens Res. 2021 Dec 16;45(2):292–307. doi: 10.1038/s41440-021-00826-8 (PMC8766285; doi:10.1038/s41440-021-00826-8)
Supplement: Supplementary file 1 — Supplementary Information [file 41440_2021_826_MOESM1_ESM.docx]

***Cpxm2* as a novel candidate for cardiac hypertrophy and failure in hypertension**

Katja Grabowski^1^, Laura Herlan^1^, Anika Witten^9^, Fatimunnisa Qadri^4^, Andreas Eisenreich^1^, Diana Lindner ^7,8^, Martin Schädlich^9^, Angela Schulz^1^, Jana Subrova^1^, Ketaki Nitin Mhatre^2^, Uwe Primessnig^2,6^, Ralph Plehm^4^, Sophie van Linthout^6,11^, Felicitas Escher^2,6,12^, Michael Bader^3,4,5,6^, Monika Stoll^9,10^, Dirk Westermann^7,8^, Frank R. Heinzel^2,6^, Reinhold Kreutz^1*^

**Affiliations**

1. Charité - Universitätsmedizin Berlin, corporate member of Freie Universität Berlin, Humboldt-Universität zu Berlin, and Berlin Institute of Health (BIH), 10178 Berlin, Institut für Klinische Pharmakologie und Toxikologie, Germany
2. Charité - Universitätsmedizin Berlin, corporate member of Freie Universität Berlin, Humboldt-Universität zu Berlin, and Berlin Institute of Health (BIH), 10178 Berlin, Department of Cardiology, Campus Virchow Klinikum, Germany
3. Charité - Universitätsmedizin Berlin, corporate member of Freie Universität Berlin, Humboldt-Universität zu Berlin, and Berlin Institute of Health (BIH), 10178 Berlin, Germany
4. Max-Delbrück Center for Molecular Medicine (MDC), Berlin-Buch, Germany
5. University of Lübeck, Institute for Biology, Ratzeburger Allee 160, 23562 Lübeck, Germany
6. German Center for Cardiovascular Research (DZHK), Partner Site Berlin, Germany
7. German Center for Cardiovascular Research (DZHK), Partner site Hamburg/Kiel/Lübeck, Hamburg, Germany
8. Clinic for Cardiology, University Heart and Vascular Center Hamburg, University Hospital Hamburg-Eppendorf, Hamburg, Germany
9. Department of Genetic Epidemiology, Institute of Human Genetics, University Hospital Münster, Münster, Germany
10. Department of Biochemistry, Cardiovascular Research Institute Maastricht, Maastricht University, Maastricht, The Netherlands
11. Charité - Universitätsmedizin Berlin, BCRT - Berlin Institute of Health Center for Regenerative Therapies, Berlin, Germany
12. Institute of Cardiac Diagnostics and Therapy, IKDT GmbH, Berlin, Germany

***Correspondence to:**

Reinhold Kreutz

ORCID-ID: https://orcid.org/0000-0002-4818-211X

Department of Clinical Pharmacology and Toxicology

Charité – Universitätsmedizin Berlin

Charitéplatz 1

10117 Berlin, Germany

phone: +49-30-450525112

fax: +49-30-4507525112

e-mail: Reinhold.kreutz@charite.de

**Supplementary information**

The pdf-File “Supplementary information” includes the expanded Materials and Methods section (Echocardiography in mice, LV ventricular catheterization in mice, Heart and kidney harvesting, Quantitative reverse transcriptase PCR, Cardiac transcriptome analysis, Immunohistochemistry) and Supplementary Figures 1 – 9, and Supplementary Table 1

**Supplementary Tables 2 - 4**

The pdf Files includes Supplementary Table 2 (Microarray results F344 vs. SHRSP), Supplementary Table 3 (Microarray results SHRSP vs. SHRSP-1^F344^) and Supplementary Table 4 (Microarray results WT SHAM vs. WT DOCA). The full data sets from microarray results are available GEO accession number GSE154327.
