# Supplementary material for: Cpxm2 as a novel candidate for cardiac hypertrophy and failure in hypertension
Source: Hypertens Res. 2021 Dec 16;45(2):292–307. doi: 10.1038/s41440-021-00826-8 (PMC8766285; doi:10.1038/s41440-021-00826-8)
Supplement: Supplementary file 2 — Supplementary Figures [file 41440_2021_826_MOESM2_ESM.docx]

**ONLINE SUPPLEMENT**

***Cpxm2* as a novel candidate for cardiac hypertrophy and failure in hypertension**

Katja Grabowski^1^, Laura Herlan^1^, Anika Witten^9^, Fatimunnisa Qadri^4^, Andreas Eisenreich^1^, Diana Lindner ^7,8^, Martin Schädlich^9^, Angela Schulz^1^, Jana Subrova^1^, Ketaki Nitin Mhatre^2^, Uwe Primessnig^2,6^, Ralph Plehm^4^, Sophie van Linthout^6,11^, Felicitas Escher^2,6,12^, Michael Bader^3,4,5,6^, Monika Stoll^9,10^, Dirk Westermann^7,8^, Frank R. Heinzel^2,6^, Reinhold Kreutz^1,*^

**Affiliations**

1. Charité - Universitätsmedizin Berlin, corporate member of Freie Universität Berlin, Humboldt-Universität zu Berlin, and Berlin Institute of Health (BIH), 10178 Berlin, Institut für Klinische Pharmakologie und Toxikologie, Germany
2. Charité - Universitätsmedizin Berlin, corporate member of Freie Universität Berlin, Humboldt-Universität zu Berlin, and Berlin Institute of Health (BIH), 10178 Berlin, Department of Cardiology, Campus Virchow Klinikum, Germany
3. Charité - Universitätsmedizin Berlin, corporate member of Freie Universität Berlin, Humboldt-Universität zu Berlin, and Berlin Institute of Health (BIH), 10178 Berlin, Germany
4. Max-Delbrück Center for Molecular Medicine (MDC), Berlin-Buch, Germany
5. University of Lübeck, Institute for Biology, Ratzeburger Allee 160, 23562 Lübeck, Germany
6. German Center for Cardiovascular Research (DZHK), Partner Site Berlin, Germany
7. German Center for Cardiovascular Research (DZHK), Partner site Hamburg/Kiel/Lübeck, Hamburg, Germany
8. Clinic for Cardiology, University Heart and Vascular Center Hamburg, University Hospital Hamburg-Eppendorf, Hamburg, Germany
9. Department of Genetic Epidemiology, Institute of Human Genetics, University Hospital Münster, Münster, Germany
10. Department of Biochemistry, Cardiovascular Research Institute Maastricht, Maastricht University, Maastricht, The Netherlands
11. Charité - Universitätsmedizin Berlin, BCRT - Berlin Institute of Health Center for Regenerative Therapies, Berlin, Germany
12. Institute of Cardiac Diagnostics and Therapy, IKDT GmbH, Berlin, Germany

***Correspondence to:**

Reinhold Kreutz

ORCID-ID: https://orcid.org/0000-0002-4818-211X

Department of Clinical Pharmacology and Toxicology

Charité – Universitätsmedizin Berlin

Charitéplatz 1, 10117 Berlin, Germany

phone: +49-30-450525112

e-mail: [Reinhold.kreutz@charite.de](mailto:Reinhold.kreutz@charite.de)

**Expanded Materials and Methods**

**Animal Models**

Cpxm2 KO mice showed normal development with no apparent abnormalities. In addition KO mice exhibited successful mating and a normal litter size (n=6-11, respectively). Genotyping showed an expected genotype distribution of homozygous wildtype (WT) and KO-mice as well as heterozygous animals.

**Echocardiography**

Echocardiography in mice was performed at baseline and 4 and 8 weeks after follow-up in anesthetized mice (2.5% isoflurane) with a high-resolution transducer (MS-400, VisualSonics, Toronto, Canada) using VEVO 2100 digital imaging system (VisualSonics, Toronto, Canada). Stroke volume (SV), cardiac output (CO), fractional shortening (FS) and ejection fraction (EF) were measured.

**LV ventricular catheterization**

At the end of the observation period after 8 weeks mice were intubated and ventilated under isoflurane anaesthesia, and a pressure-volume catheter (Millar Instruments, Houston, USA) was inserted into the left carotid artery and advanced into the left ventricle. The LV end-systolic volume (LVESV), LV end-diastolic volume (LVEDV), LV end-diastolic pressure and isovolumic relaxation constant (Tau_w_) were measured.

**Heart and kidney harvesting**

Hearts and kidneys were dissected under deep anaesthesia. Hearts were arrested in diastole by rinsing in 1 M KCl solution and subsequently blotted dry. The atria were trimmed away and hearts and kidneys were weighed. LV tissue was separated from septum and right ventricle for determination of LV weight. LV tissue and kidney were snap frozen and used for further analyses.

**Quantitative reverse transcriptase PCR**

First-strand cDNA synthesis for quantitative reverse transcriptase PCR (qPCR) purposes (n = 5-10) was carried out on 2 μg of total RNA in a 20 μl reaction using the First Strand cDNA Synthesis Kit (Fermentas Life Sciences, St. Leon-Rot, Germany) as recommended by the manufacturer. Appropriate primers were designed with the Primer Express software and synthesized by TIB Molbiol (Berlin, Germany, Table S1). The 7500 Fast Real-Time PCR System and the Fast SYBR Green Master Mix (Applied Biosystems, Darmstadt, Germany) was used to perform the assays according to manufacturer’s recommendations. Relative quantification was done using the ΔΔ-ct method. Every sample was measured in triplicate. To normalize mRNA expression data Hprt (hypoxanthine guanine phosphoribosyl transferase) was used as reference gene.

**Cardiac transcriptome analysis**

RNA was isolated from the free wall LV by the TRIzol reagent (Invitrogen, Karlsruhe, Germany) according to the manufacturer's instructions and dissolved in DEPC-treated H2O. The quality and concentration of the purified total RNA were confirmed by spectroscopy (Nanodrop ND-1000 spectrophotometer; Thermo Fisher Scientific, Waltham, Massachusetts, USA).

In the rat model, we performed whole transcriptome analysis on LV tissue of male F344, SHRSP-1F344 and SHRSP (n = 8, respectively) using Illumina RatRef-12 Expression BeadChips. In the mouse model whole transcriptome profiling was performed on LV tissues from male SHAM and DOCA animals of both WT and KO mice (n = 3, respectively). The RNA was processed and hybridized on the GeneChip Mouse Gene 2.0 ST Array (Affymetrix UK Ltd., High Wycombe, UK). Total RNA for both microarray workflows was prepared for hybridization according to the manufacturer’s instructions.

**Immunohistochemistry**

For immunohistochemistry whole hearts were rinsed in 10% KCl solution, washed with PBS fixed in 4% PFA overnight and processed for embedding in paraffin blocks. For immunostaining, 5 µm thick sections were performed using a rotation microtome (Microm, ThermoFisher, Germany), air dried over night at room temperature (RT). The sections were then deparaffinized with xylol and rehydrated using descending concentrations of ethanol and washed in 1x PBS (pH 7.4). The sections were then boiled for 20 minutes in antigen retrieval buffer (citrate buffer, pH 6.0). The unspecific bindings were blocked using 10% normal donkey serum in wet chamber at RT. The sections were then incubated with primary antibodies (CPXM2 and DHPR, 1:100) over night in wet chamber at 4°C. Next day sections were washed in 1x PBS and incubated with secondary antibodies conjugated to either with Cyanin-3 or Alexa-488 (1:300) for 2h at RT, washed in PBS and cover slipped using a mounting medium containing DAPI (Vectashield Laboratories). To distinguish the cell wall of cardiomyocytes in paraffin sections, the sections were incubated with wheat germ agglutinin conjugated to Alexa-488 (1:1000) overnight at 4°C. The images were taken using a fluorescence microscope (Zeiss LSM800 or Keyence BZ9000, Germany).

**Supplementary Figure 1.**

Mean arterial blood pressure (MAP, **a**) and relative left ventricular weight (RLVW, **b**) in 14 weeks old F344 (white), SHRSP-1^F344^ (grey) and SHRSP (black) rats, n = 6-8, respectively. MAP and RLVW is displayed as mean ± SEM. Statistical analyses were performed using one-way ANOVA with Bonferroni post hoc test, *p < 0.001 vs. all other groups.

**
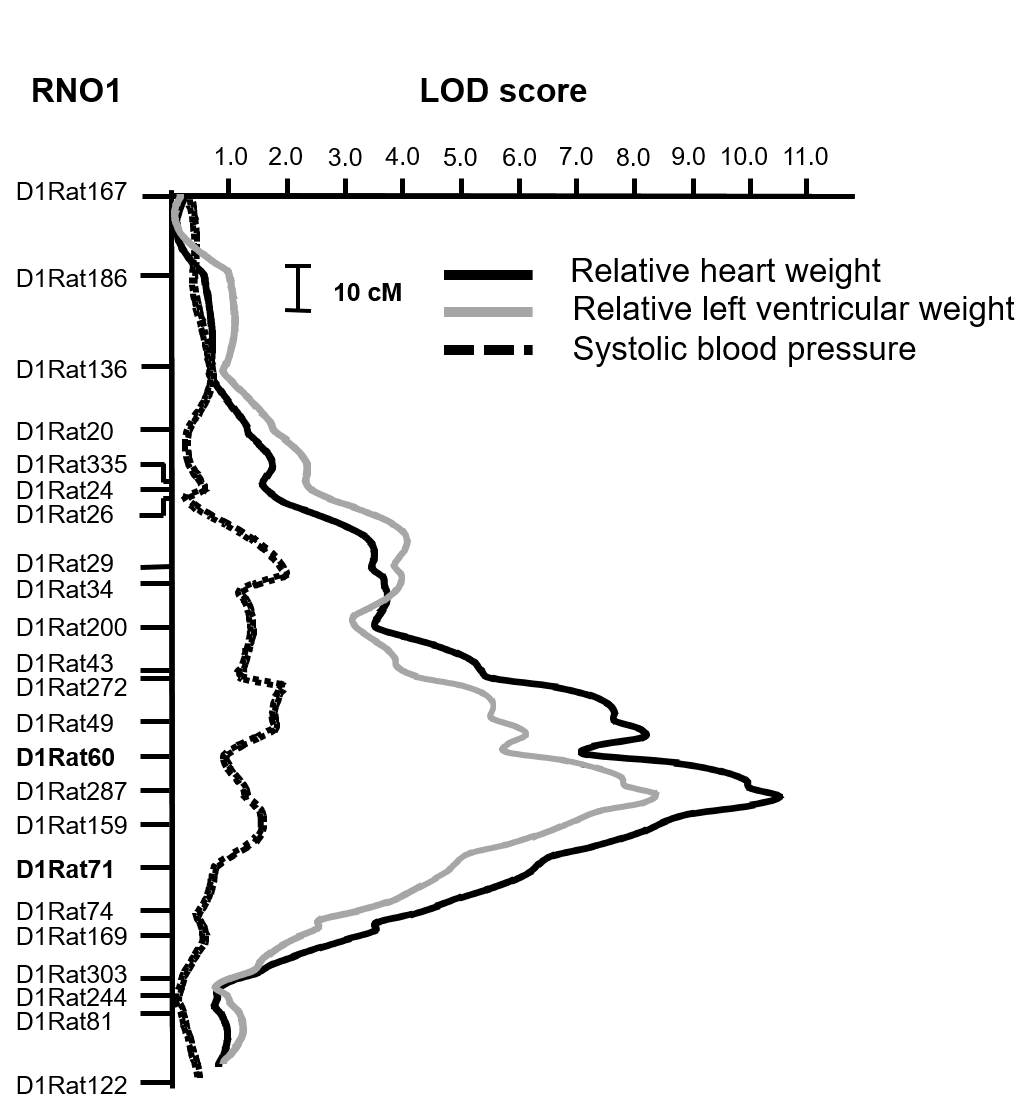
**

**Supplementary Figure 2**

Quantitaive trait loci of relative heart weight (RHW, black line), relative left ventricular weight (RLVW, grey line) and systolic blood pressure (dotted line) on rat chromosome 1 (RNO1) by generating (SHRSP x F344) F2 cross (n = 232) at 14 weeks of age. LOD score of RHW = 10.51, LOD score of RLVW = 8.38; 1-LOD and 2-LOD intervals are positioned between genetic markers D1Rat60 to D1Rat71 (bold). Distance in centi Morgan (cM).

**Supplementary Figure 3**

Mean left ventricular *Cpxm2* mRNA expression in 4 weeks, 8 weeks and 14 weeks old F344 (white), SHRSP-1^F344^ (grey) and SHRSP (black) rats, n = 7-8, respectively. Expression levels are displayed as fold changes of *Cpxm2* expression in 4 weeks old F344 ± SEM. The mRNA expression of *Cpxm2* was normalized against *Hprt* expression; Statistical analyses were performed using one-way ANOVA with Bonferroni post hoc test, * p < 0.05 vs. F344 at the same age, ** p < 0.001 vs. all other groups at the same age.

**Supplementary Figure 4**

Correlation between individual *Cpxm2* mRNA expression and cardiac phenotypes in Cpxm2 wild type mice following DOCA treatment, **a** *Cpxm2* mRNA and relative left ventricular weight [LVW/tibia length (TL)] r = -0.872, p = 0.054; **b** *Cpxm2* mRNA and natriuretic peptide A (*Nppa*) mRNA expression r = -0.381, p = 0.527; **c** *Cpxm2* mRNA and LV ejection fraction (EF) r = -0.003, p = 0.996

**Supplementary Figure 5**

Determination of renal damage in *Cpxm2* wild type (white) and knock-out (black) after 8 weeks SHAM or DOCA treatment, n = 5-10, respectively.  **a** Renal interstitial fibrosis was analyzed after picro-sirius red staining including 3 fields of view per animal (n = 5-10); **b** Glomerusclerosis index and **c** Tubular injury was determined after periodic acid-Schiff (PAS) staining using a semiquantitative scoring approach; Statistical analyses were performed using one-way ANOVA with Bonferroni post hoc test, *p < 0.05 vs. SHAM groups, ** p < 0.05 vs. WT SHAM.

**Supplementary Figure 6**

Mean left ventricular (LV) mRNA expression of natriuretic peptide A (*Nppa*) in *Cpxm2* wild type (WT, white) and *Cpxm2* knock-out (KO, black) mice after 8 weeks SHAM or DOCA treatment, n = 5-10, respectively. Expression levels are displayed as fold changes compared to WT SHAM ± SEM. The expression of Nppa was normalized against Hprt; Statistical analyses were performed using one-way ANOVA with Bonferroni post hoc test, *p < 0.05 vs. all other groups.

**Supplementary Figure 7**

Cpxm2 expression in left ventricular (LV) tissue of *Cpxm2* wild type (WT) and *Cpxm2* knock-out (KO) mice. **a** In qPCR analysis, expression levels are displayed as fold changes of Cpxm2 expression in SHAM or DOCA treated *Cpxm2* WT and KO mice compared to WT SHAM ± SEM. The expression of Cpxm2 was normalized against Hprt; Statistical analyses were performed using one-way ANOVA with Bonferroni post hoc test, * p < 0.05 vs. corresponding DOCA group. **b** Representative image of conventional PCR experiment displaying PCR product of *Cpxm2* of 124 bp using primer with location in Exon 1 and 3 of *Cpxm2* in *Cpxm2* WT and *Cpxm2* KO mice. A 50 bp marker (M) and a negative probe (NTC) are shown.

**a**

**b**

**Supplementary Figure 8**

Scatterplot of the arrays along the first two principal components of rat experiments (a) and mouse experiments (b; Cpxm2 wild-type [WT] and knock-out mice [KO], treated with DOCA [DOCA] or SHAM treated [SHAM]). With PCA we here show the multivariate data vector of each array into a two-dimensional plot, such that the spatial arrangement of the points in the plot reflects the overall data (dis)similarity between the arrays.


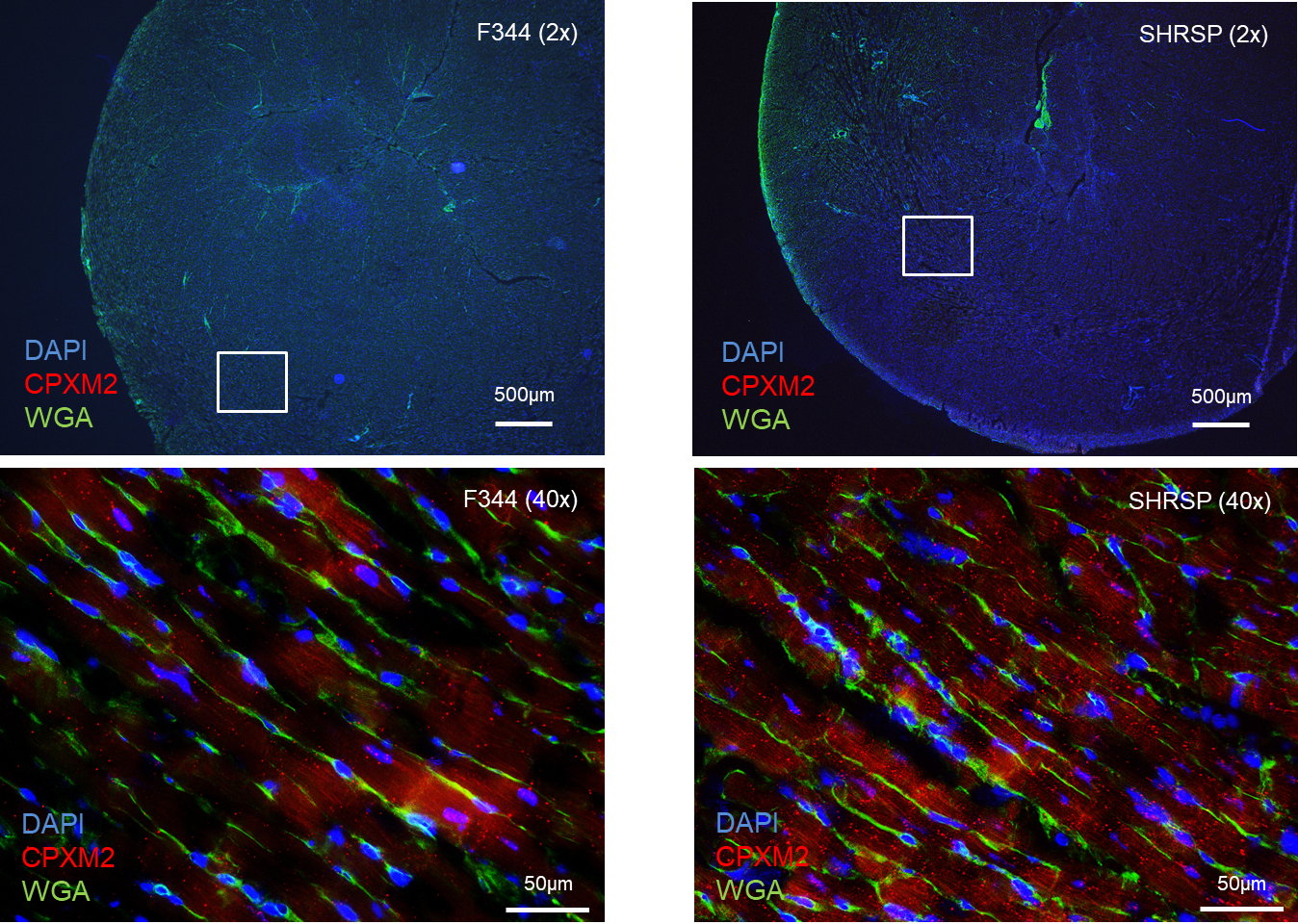


**Supplementary Figure 9**

Paraffin sections of hearts from SHRSP (right sections) and F344 (left sections) were analyzed with immunofluorescent staining of CPXM2 (red, cy3), cardiomyocyte cell wall (green, WGA Alexa488) and nuclei (blue, DAPI).

The top panels show the overview of the left ventricle (magnification 2x, scale bar 500 µM) and indicate with the white frame insert where the corresponding pictures with higher resolution (magnification 40x, scale bar 50µM) were obtained as shown in the corresponding Figure 1d.

**Supplementary Table 1.** Primer sequences

| **Gene** | **GenBank**  **accession no.** | **Species** | **Sense Primer**  **5’ → 3’** | **Antisense Primer**  **5’ → 3’** | **bp** |
| --- | --- | --- | --- | --- | --- |
|  |  |  |  |  |  |
| *PCR Genotyping* | | | | | |
| *Cpxm2* wild type | NM_018867.5 | Mouse | TGGCCCTGGCTGGAGTCAGAGC | TACCTGGTGGAGGCGTCTCTGC | 273 |
| *Cpxm2* knock-out | n.a. | Mouse | GCAGCGCATCGCCTTCTATC | CCTTAGGCTGTGCCAGCGAGC | 359 |
| *Quantitative reverse transcriptase PCR analysis* | | | | | |
| *Cpxm2* Ex 1-3 | NM_018867.5 | Mouse | CCACCAGGTAAAAATAGCAACAGAA | GACCAAGAGGTGGGCAACTC | 124 |
| *Nppa* | NM_008725.2 | Mouse | GGATTTCAAGAACCTGCTAGACCA | CTCCCCAGTCCAGGGAGG | 145 |
| *Hprt* | NM_013556.2 | Mouse | GCTATAAGTTCTTTGCTGACCTGCT | TTTTATGTCCCCCGTTGACTG | 134 |
|  |  |  |  |  |  |
| *Cpxm2* | NM_001106306.1 | Rat | CATCCCTGAGTGGTTTCTGTCTG | TGCTACAACCAGCTCACCCC | 127 |
| *Hprt* | NM_ 012583.2 | Rat | CTCATGGACTGATTATGGACAGGACT | TCCAGCAGGTCAGCAAAGAAC | 127 |

*Cpxm2*, carboxypeptidase X (M14 family), member 2; *Nppa*, natriuretic peptide type A; *Hprt*, hypoxanthin phosphoribosyltransferase
