# Supplementary material for: Cpxm2 as a novel candidate for cardiac hypertrophy and failure in hypertension
Source: Hypertens Res. 2021 Dec 16;45(2):292–307. doi: 10.1038/s41440-021-00826-8 (PMC8766285; doi:10.1038/s41440-021-00826-8)
Supplement: Supplementary file 3 — Supplementary Table 2 [file 41440_2021_826_MOESM3_ESM.pdf]

Supplementary Table 2. Microarray results F344 vs. SHRSP
The full data sets from microarray results are available via GEO accession number GSE154327.

PROBE_ID	SYMBOL	ENTREZ_GENE_ID	logFC	adj.P.Val
4120021	RGD1565371_predicted*	501140	-4.818041851	4.61E-20
1190731	Cct6a_predicted	288620	-4.317422136	2.91E-25
1660403	Mrpl18_predicted*	292244	-4.314620497	1.75E-24
60576	RGD1562905_predicted	292539	-4.113679982	2.19E-23
2260064	Myl7_predicted	289759	-3.904835547	1.49E-12
6770563	Ephx2	65030	-2.399684625	3.97E-16
**2230576**	**Bnip3***	**84480**	**-2.359720187**	**7.29E-12**
4920575	Adhfe1	362474	-2.28606457	2.08E-16
2760022	LOC311772	311772	-2.257341856	8.78E-17
3610286	Ccl5	81780	-2.116082102	2.67E-14
6620239	Zfp364_predicted	362002	-2.010160656	1.12E-13
580162	Usmg5*	171069	-1.987377681	5.08E-12
5340301	RGD1563903_predicted	365613	-1.963673951	1.54E-13
6760575	Cds1	81925	-1.95673114	7.86E-17
1050746	Rragd_predicted	297960	-1.942494395	2.96E-18
4850500	Hamp	84604	-1.774461672	0.006076878
6520132	Nkg7	171062	-1.773935052	1.49E-12
520064	RGD1310766_predicted	364147	-1.745857148	2.19E-12
3360487	Trh	25569	-1.691897452	0.014893719
3940494	Lgmn	63865	-1.686915963	2.67E-14
6200541	Ubd	29168	-1.592123398	1.44E-09
1740072	C2	24231	-1.570744362	1.44E-10
2480403	Gzma	266708	-1.544872826	3.20E-13
7100348	Psmb8	24968	-1.457994008	9.68E-09
6400364	RT1-Db1	294270	-1.445958265	3.50E-09
1400736	RGD1564247_predicted	499574	-1.44203446	1.16E-16
460167	LOC682869	682869	-1.396881005	5.34E-08
430161	Gzmc	171290	-1.393829755	2.51E-06
6770397	Serpinb6a	291085	-1.279518754	1.73E-13
5570114	Slpi	84386	-1.258114041	0.000128986
5670133	LOC502009	502009	-1.229693858	6.90E-07
7050270	Rps18	294282	-1.210088678	2.40E-11
1090048	RT1-A1	24973	-1.192986648	1.15E-07
450397	Cd63*	29186	-1.186659215	0.000256424
580239	Picalm*	89816	-1.154190124	1.24E-12
2570088	Per2	63840	-1.153413061	0.002147244
3710025	Cbx7	362962	-1.135445778	1.40E-09
3390025	Dhx36_predicted	310461	-1.107639198	5.40E-14
2260411	Cd8a	24930	-1.092943229	1.41E-11
2680754	RGD1563224_predicted	500874	-1.08998019	4.92E-08
430278	Rims1	84556	-1.07359358	1.35E-08
460494	Acat1	25014	-1.072651946	2.51E-09
3120577	Mrc1_predicted	291327	-1.06267038	2.42E-05
1850022	Polr2i_predicted*	292778	-1.060830493	5.16E-13
2940500	RGD1564936_predicted	361875	-1.059250625	2.84E-12
4480671	LOC361929	361929	-1.05870798	1.20E-12
6900563	Rhoj	299145	-1.058140421	2.18E-08
2760390	LOC501744	501744	-1.053536799	6.11E-07
6110020	RGD1564247_predicted	499574	-1.050298746	3.20E-12
**510408**	**Slc22a18***	**309131**	**-1.048787287**	**1.28E-09**
3610279	Fhl2	63839	-1.041230195	3.58E-07
5670750	Klkb1	25048	-1.040593587	4.08E-13
1400082	Inha	24504	-1.027015304	0.000640108
4070138	Zcchc9	309986	-1.025817966	1.47E-10
5390022	Wfdc1	171112	-1.025047436	2.50E-05

450270	Akr1b10	296972	-1.022786383	2.94E-06
4570142	Ucp2*	54315	-1.018752193	5.34E-09
6400253	Mrpl48_predicted*	293149	-1.011366999	3.85E-11
1580053	LOC501015	501015	-0.990100462	0.010359054
2510053	Irx2	306657	-0.982539204	4.65E-06
4540487	Ifi44	310969	-0.976567718	4.40E-06
60520	Lamc2	192362	-0.975135888	3.28E-10
1980341	Lancl1	114515	-0.938507596	3.63E-10
4610273	MGC72973*	361619	-0.937815424	0.004220149
4730377	Tnrc6_predicted	308971	-0.934419977	1.11E-12
430100	RGD1311381_predicted	300870	-0.923027857	0.000955541
3710278	LOC290704	290704	-0.922389369	0.000419143
580647	Cxcl16	497942	-0.914365898	2.48E-06
1820678	Prf1	50669	-0.905319019	8.41E-08
5890411	RT1-Da	294269	-0.90229582	2.64E-05
2470440	Rgs2	84583	-0.899812413	0.003547398
5690242	Klre1	297645	-0.895195238	4.23E-07
630010	Lztr2	89868	-0.886646603	0.000858813
3450349	Becn1	114558	-0.884923621	4.54E-12
6110288	Klrd1	25110	-0.863832836	1.25E-08
3610037	RGD1303232	309381	-0.862087573	1.65E-06
5390164	Hdac2	84577	-0.857905322	3.44E-09
6770079	LOC501066	501066	-0.857447592	4.55E-05
6100601	Serpinb6b	364705	-0.856949026	2.10E-09
3610450	LOC361172	361172	-0.846235731	3.83E-07
610438	Clic2	294141	-0.846133846	2.34E-09
2060136	RGD1305687_predicted	287453	-0.843678464	8.33E-11
1690446	Gnpda2_predicted	289608	-0.841222758	1.46E-12
2650064	Gcgr	24953	-0.838244763	1.79E-05
510154	Cd3g	300678	-0.835712747	1.33E-09
1170040	Casq2	29209	-0.834696761	4.73E-07
5690546	Prmt5_predicted	364382	-0.828805839	2.78E-07
770142	Gcgr	24953	-0.825267058	0.000313724
1580446	Tna_predicted	316099	-0.824040203	4.02E-06
3130601	Lbp	29469	-0.821841419	1.93E-05
4610402	Uxt	299313	-0.821656876	3.10E-09
4570577	Gzmk	29165	-0.820070229	2.11E-10
2340088	RGD1562351_predicted	499990	-0.819929618	4.68E-10
1050465	C4-2	406161	-0.805155519	0.001120302
6040671	RGD1305020_predicted	311575	-0.803869305	1.45E-12
2030079	Vldlr*	25696	-0.798071286	0.000332983
7100603	LOC310968	310968	-0.79705978	1.22E-09
1340390	Abca2	79248	-0.794611891	5.74E-09
3360504	Spsb1_predicted	313722	-0.789155594	0.008018959
2640292	Mgl1	64195	-0.782724102	3.14E-05
770193	LOC498174	498174	-0.781013281	1.42E-11
4230048	RGD1564762_predicted	313052	-0.775927948	0.002254496
2260039	Rsn	65201	-0.775694177	1.97E-07
7050097	Dock11	313438	-0.771102831	1.50E-07
630133	RT1-M10-1	414787	-0.765225388	5.03E-05
5390092	LOC361990	361990	-0.760575957	1.10E-10
360041	Tgm1	60335	-0.760545851	0.044924244
5900463	Ms4a4a_predicted	361734	-0.759175428	0.007022235
5340148	Ptpro	50677	-0.758777368	4.02E-06
4570112	Cml3	113892	-0.756902778	2.14E-10
4810020	LOC305633	305633	-0.754496294	2.04E-06
3360670	Agl_predicted	362029	-0.754297407	2.77E-09
3450500	Bloc1s2*	293938	-0.751434609	5.34E-09

5910215	RGD1565715_predicted	361149	-0.750203646	1.77E-05
110309	Smarca3_predicted	295568	-0.749834146	2.39E-07
3140538	Pom210	58958	-0.74894468	1.69E-09
4480500	Klf15	85497	-0.745606029	0.000722195
2360100	LOC310395	310395	-0.736178822	0.000211587
5900039	Ndufa10	314071	-0.735283735	3.72E-08
770242	Pex3	83519	-0.734818729	1.69E-09
2470672	LOC302996	302996	-0.733772091	1.12E-06
60184	RT1-149	414784	-0.721978259	5.13E-07
4670008	Efnb3_predicted	360546	-0.717278315	0.007085363
1660315	Stat3	25125	-0.712706654	0.003553638
3610156	Fdx1	29189	-0.711988428	0.023778009
610113	Ces3	113902	-0.710342625	0.001056562
3130609	Wasl	296936	-0.710206458	9.30E-06
1660195	Cacna2d1	25399	-0.709927115	1.02E-08
6200154	Crsp6	300367	-0.709155751	3.35E-09
6840594	RGD1562408_predicted	501502	-0.708209349	2.52E-07
840463	Gnao	50664	-0.70786508	0.000680402
2570156	Jak3	25326	-0.706169777	7.91E-05
5080372	Hfe	29199	-0.70364269	3.80E-08
2510273	Hla-dmb	294273	-0.702104514	1.32E-05
4010154	Igsf7	287813	-0.699047614	1.19E-07
2470082	RGD1310039	361747	-0.698358271	1.01E-09
4060609	Lsamp	29561	-0.696434107	9.43E-06
2970364	Bcl6_predicted	303836	-0.695309135	0.014430139
6020180	Plag1	297804	-0.694302681	5.25E-08
3780402	Cd7_predicted	303747	-0.693915539	6.64E-07
3940039	RGD1562920_predicted	292486	-0.689878782	1.04E-07
4540309	Ptafr	58949	-0.685403362	5.38E-10
5290609	Ezh1_predicted	303547	-0.685351027	0.001330554
5690524	RGD1310724	360803	-0.682472601	2.65E-06
2450746	Ppp1r14c	171010	-0.678190261	1.41E-05
4010168	Irx4_predicted	306655	-0.677292106	0.005208842
2850537	Bok	29884	-0.672197803	5.90E-05
5670600	Tcrg	24821	-0.66820356	2.81E-05
940484	LOC498277	498277	-0.663576028	5.94E-05
5220364	Ifi27l	170512	-0.662848918	0.000172618
5340446	RGD1562214_predicted	500305	-0.660235689	1.59E-05
7100408	Fcgr3	116591	-0.656795956	8.64E-10
2570280	Rbm8_predicted	295284	-0.656570446	1.05E-07
3060095	RGD1560364_predicted	363087	-0.653473893	2.28E-10
6450129	Slc16a1	25027	-0.648635593	4.98E-06
5910202	Hla-dma	294274	-0.648303304	6.14E-06
6370181	Abcb4	24891	-0.646363406	1.87E-09
580563	LOC499300	499300	-0.642253296	1.71E-05
130091	Coro1a	155151	-0.638572963	1.99E-05
4070164	Slc4a3	24781	-0.638512492	0.000651215
2360093	Aox1	54349	-0.638142056	5.02E-05
4590463	Trp53i13_predicted	287550	-0.636071622	9.12E-10
6550494	Cirbp	81825	-0.634952046	0.011614025
1240372	Pycard	282817	-0.634529399	0.000751743
1770504	Cbr1	29224	-0.631827204	0.000989454
5720215	Ifngr1	116465	-0.626692622	0.001131277
5050100	Drd4	25432	-0.625561571	0.008546366
1170451	Vil2	54319	-0.62453514	6.17E-07
4810129	Arhgap9	362893	-0.622755683	8.05E-07
6760239	Clns1a*	65160	-0.620175951	5.53E-08
6020044	MGC94190	288616	-0.617341388	1.69E-05

1450204	Eno1	24333	-0.615207119	1.49E-06
2760048	RGD1304816_predicted	362528	-0.614518003	5.17E-08
1500170	Ifi30	290644	-0.612592122	3.96E-08
6380132	Bcl11b_predicted	314423	-0.603822743	1.57E-06
3170670	Prr3	361788	-0.602980388	2.25E-06
2070605	Gstm2	24424	-0.597269393	0.006672361
610725	Sema4a	310630	-0.595768644	5.95E-05
5550279	Hmox2	79239	-0.592816451	5.81E-10
6840736	Abr_predicted	287537	-0.590870926	0.003877755
1500181	RGD1311703	293160	-0.589461737	6.28E-07
1450706	C1qg	362634	-0.589339884	0.039450498
2030121	Art5	259167	-0.588408987	0.000174568
1340736	Itgad	64350	-0.586403634	1.85E-06
7000403	Argbp2	114901	4.25615964	6.82E-20
3990280	Card9	64171	3.287536236	2.08E-17
1190487	Ccdc53_predicted	299707	3.241095762	3.37E-21
5050170	Nppa	24602	2.721124887	1.09E-10
3290551	Hmgn1	360704	2.608896753	4.61E-20
6660368	LOC363865	363865	2.414392283	9.69E-18
6220377	Acta1	29437	2.368028959	5.20E-10
3130670	Mrpl17*	171061	2.350335606	1.12E-17
1190692	Tcte1l	363448	2.116779373	8.07E-15
7000324	Spp1	25353	2.089790605	6.75E-05
2030685	LOC497757	497757	1.824878062	1.19E-18
4480082	Defb1	83687	1.822195675	4.73E-11
2570338	LOC500733	500733	1.795350091	5.10E-13
4570731	RGD1309065_predicted	315179	1.775040857	9.44E-07
2450014	RGD1562162_predicted	366481	1.752221406	6.25E-14
940184	Zadh1	299194	1.743595867	1.14E-17
5220138	LOC362725	362725	1.729323877	2.90E-15
6840086	Kcne1	25471	1.719144117	7.75E-06
7000332	Atp6v1g2	368044	1.707708288	2.91E-11
1500768	Nppb	25105	1.675129807	1.74E-06
3830035	Slc3a1	29484	1.634776701	6.57E-12
7040647	Lcn2	170496	1.616555289	0.007075062
6840593	Atcay_predicted	362826	1.612655976	0.001591516
4780136	LOC497685	497685	1.602150858	9.87E-14
3710504	Anxa3	25291	1.592497358	2.18E-10
3290487	Xylt1	64133	1.574630896	4.45E-13
2340079	Aldh1a1*	24188	1.563667243	5.05E-11
1570390	Atad2_predicted	314993	1.55685408	1.02E-09
60575	Prl8a4	59088	1.493699929	1.01E-12
2690551	LOC297481	297481	1.47555739	3.77E-13
2060014	Tuba8	500377	1.467898034	0.000182461
1580408	LOC683522	683522	1.447069542	5.88E-15
380072	Tnn_predicted	304913	1.433617927	4.86E-15
4730402	Trem2_predicted	301227	1.431840348	3.89E-09
3940053	Csprs_predicted	316579	1.414242298	3.81E-13
940301	LOC361990	361990	1.406503441	7.21E-13
3450600	LOC499255	499255	1.390104448	1.41E-11
450112	RGD1566241_predicted	364381	1.389817233	7.25E-14
3440270	LOC364343	364343	1.365878373	1.20E-12
3170341	Serpinf1	287526	1.354368122	1.65E-12
6650397	LOC499660	499660	1.352178868	2.91E-11
1780551	RGD1560397_predicted	360596	1.348153243	5.33E-14
2850546	LOC689695	689695	1.345795875	4.54E-13
2510735	RGD1564029_predicted	499598	1.343025723	9.39E-12
2340377	Olr1751_predicted	302825	1.340350015	9.20E-13

6100053	LOC499581	499581	1.337676661	5.33E-14
380563	RGD1566178_predicted	364823	1.336997726	8.99E-11
670520	LOC363701	363701	1.335267539	8.07E-11
460113	LOC361140	361140	1.333219994	4.08E-13
3130035	Mkks	311456	1.328953834	9.25E-09
6180414	Sema3f_predicted	315996	1.327630003	7.23E-11
4050619	Irak1_predicted	363520	1.323982442	2.67E-14
4010161	Hoxc4_mapped	24459	1.321626197	5.08E-12
6980082	Mocs1_predicted	301221	1.312364006	3.90E-12
6450041	Rps24	81776	1.311455213	2.84E-12
4010095	Asgr2	29403	1.305066931	2.68E-10
1740577	Dclre1b	310745	1.292780999	3.10E-11
4480025	Olr29_predicted	404896	1.292441202	1.54E-13
2640592	RGD1311103_predicted	301100	1.285726927	9.39E-12
730017	Dhdds	298541	1.285204941	4.28E-09
3140500	Slc30a2	25362	1.266994455	2.70E-14
4060020	Ttyh3_predicted	304315	1.258416862	5.90E-13
3390162	Eml1	362783	1.257427875	1.71E-12
2630341	Smp2a	24902	1.255607049	6.87E-14
6860167	RGD1564674_predicted	499649	1.254198733	3.56E-09
2510390	RGD1565435_predicted	502743	1.251114103	1.64E-12
4760379	Abcg4	300664	1.237877114	5.32E-12
6510722	Flrt2_predicted	299236	1.234245001	2.67E-14
1570044	Slc24a4_predicted	314396	1.230549122	1.33E-12
3450746	Qscn6	84491	1.226570176	0.000199063
1990138	Olr677_predicted	405950	1.225648708	5.36E-12
1690091	Thbs4	29220	1.221344499	1.45E-05
1170184	LOC503197	503197	1.216475112	5.79E-12
**5570040**	**Cpxm2_predicted***	**293566**	**1.216366362**	**1.19E-11**
2190341	RGD1307814_predicted	362559	1.213540566	2.42E-12
3450605	Olr552_predicted	405943	1.213268249	4.21E-13
5360433	Srd5a2	64677	1.20557738	2.19E-07
6370082	Indo	66029	1.205541054	2.07E-10
5080333	Rgs14	114705	1.195464025	1.39E-13
2120064	RGD1562963_predicted	498729	1.191833475	2.79E-13
4060053	RGD1560462_predicted	365664	1.187927229	3.93E-14
4200670	Prss8	192107	1.180190929	1.32E-12
1450184	Trim21_predicted	308901	1.179272725	8.12E-14
70270	Cyr61	83476	1.170539421	0.000333865
5690025	Bphl	361239	1.165389875	4.69E-13
2190739	LOC499316	499316	1.160059659	3.84E-13
1170112	RGD1564914_predicted	500616	1.149447202	1.15E-11
2360440	Ms4a8b_predicted	361733	1.147055636	2.66E-12
4230102	LOC499031	499031	1.144352745	1.44E-13
1230242	Rpl3l_predicted	287122	1.137387421	3.65E-11
4480093	Dscr1	266766	1.134918443	6.98E-06
2570408	Rab3d	140665	1.130080747	2.11E-09
70176	LOC303730	303730	1.126918194	1.28E-13
3990722	Slit2	360272	1.126438761	7.35E-11
5220402	Mybph	83708	1.123360366	1.03E-10
5890563	Tcam1	59305	1.122690263	4.92E-12
1990692	LOC295015	295015	1.116206192	1.06E-11
1450592	Myl6_predicted	362816	1.111057466	1.27E-09
2970368	RGD1309310_predicted	303677	1.110278096	2.56E-12
4480288	Ptrh1_predicted	362113	1.100849701	5.36E-12
3130722	Comt	24267	1.095805329	1.62E-12
3170086	Cdh19	360835	1.09571499	1.15E-12
4050458	RGD1561273_predicted	302093	1.094017578	3.57E-13

610682	Ccr7	287673	1.093808852	7.01E-11
7100195	LOC500675	500675	1.088647883	9.48E-11
2120364	RGD1566007_predicted	367332	1.086053342	2.89E-12
4210333	Olr1201_predicted	405106	1.084824901	2.58E-09
1740438	Vnn1	29142	1.080100553	5.90E-10
1500725	LOC360997	360997	1.076857273	4.02E-06
870725	Tas2r41	246219	1.074917644	1.72E-11
4810048	RGD1563144_predicted	498486	1.073113493	1.08E-09
3060722	Scand1_predicted	362252	1.068579381	1.27E-10
5890347	RGD1559841_predicted	307816	1.064896029	1.15E-11
1410056	Rps16*	140655	1.063694458	4.37E-12
1580070	Cyp4a10	50549	1.061592583	5.10E-13
2810095	RGD1559767_predicted	367806	1.0608392	1.64E-12
1090358	Tfam	83474	1.059974771	2.29E-15
6380403	RGD1311530_predicted	300967	1.056052303	5.83E-05
6770594	Mx2	286918	1.051526507	0.000707766
4590019	Hod	171160	1.050880227	3.09E-07
360520	LOC305332	305332	1.050475002	5.94E-10
5670647	Slc38a4	170573	1.050143687	5.91E-12
1740673	Spn	24796	1.046656746	3.65E-12
6110725	RGD1566402_predicted	500127	1.046114536	4.99E-11
2850161	Olr1597_predicted	405222	1.043497754	1.93E-11
4200129	LOC500033	500033	1.041702754	3.49E-09
1450070	Pxmp4	282634	1.041152664	5.36E-12
3450504	LOC290864	290864	1.039541888	9.39E-12
1050008	Cfh	155012	1.037669636	4.22E-10
2940215	Serpinh1	29345	1.0366745	2.72E-11
6650692	Olr557_predicted	404871	1.035855777	3.66E-10
50270	Npepps	50558	1.035091684	1.11E-11
5900040	Ddah1	64157	1.033973086	5.20E-09
4560170	RGD1308048_predicted*	298557	1.030461052	6.44E-08
2900707	Trpv6	114246	1.030378097	1.46E-09
3290603	Usf2	81817	1.025371608	1.06E-07
5550164	Spred2_predicted	305539	1.02506152	1.06E-12
5290671	Rage	362787	1.020704528	5.11E-12
2810435	Metap1_predicted	295500	1.009594305	9.43E-11
3170113	LOC687424	687424	1.008948635	8.27E-11
4780451	Ctnnd1_predicted	311163	1.008149088	1.40E-06
130358	LOC362985	362985	1.007661095	3.28E-10
5860040	Perq1_predicted	304378	1.00517979	4.32E-13
5050041	RGD1562251_predicted	497906	1.001575486	2.81E-12
5290605	RGD1564133_predicted	502612	0.998363053	5.54E-12
6200164	LOC502536	502536	0.996722172	6.60E-12
2900162	Lad1_predicted	313325	0.995519556	1.34E-07
6940181	LOC501534	501534	0.994109439	5.33E-10
780300	LOC288396	288396	0.986644244	1.70E-11
4150025	Olr1340_predicted	300640	0.981773565	2.71E-12
1690239	Enah	360891	0.980342301	7.89E-08
3360102	Lrrc42	298309	0.973229515	8.07E-11
1570167	Tmprss7_predicted	288118	0.972318217	6.95E-15
4200592	Ednrb	50672	0.972088945	6.77E-07
1340441	Olr1568_predicted	363826	0.971930853	2.65E-12
2970685	RGD1306233_predicted	296608	0.97056966	5.26E-10
3290670	LOC498899	498899	0.966777804	2.52E-13
1190465	Drd2	24318	0.963865741	3.71E-06
2850528	LOC294726	294726	0.960696983	4.99E-11
3870433	Lgals7	29518	0.954627728	3.76E-09
3120082	ORF19	367328	0.95283825	4.96E-12

4760035	Pla2g7	301265	0.951120985	2.88E-06
7100403	Hdac4_predicted	363287	0.947911459	2.03E-08
5910450	Ccnd1	58919	0.94652339	0.002401648
1230433	LOC500046	500046	0.929549907	5.54E-12
50484	RGD1560542_predicted	499847	0.927533117	1.57E-07
5220609	RGD1563697_predicted	499353	0.925710261	4.00E-08
5270538	Olr1690_predicted	294162	0.924338122	3.41E-10
70706	Mta2	361724	0.922545885	3.19E-11
1240711	RGD1560110_predicted	366565	0.918827252	1.28E-10
3520711	Rhcg	293048	0.91524137	1.74E-06
4570184	Pafah2	313611	0.910145281	3.87E-12
5890592	Birc6_predicted	313876	0.909417474	3.84E-08
5130280	Fzd9	266608	0.909064765	6.82E-07
**4850673**	**RGD1304686***	**293673**	**0.908597237**	**4.08E-13**
6660039	Chek1	140583	0.905491608	2.59E-10
1990136	Chchd3_predicted	296966	0.902904502	5.34E-12
610731	Pfn2	81531	0.897668997	4.00E-05
5050164	Dhx29_predicted	294741	0.893539577	1.72E-11
6100050	Degs1	58970	0.892674651	2.03E-08
2320746	LOC362012	362012	0.889691418	3.80E-10
4920598	Agtrl1	83518	0.885502854	0.044924244
4590722	Adcy5	64532	0.883431603	5.46E-10
6020176	Sytl2_predicted	361604	0.881889949	3.07E-09
540086	Trim2	361970	0.880816923	2.74E-10
1850546	Ankrd23_predicted	316330	0.874752758	0.000235192
780605	Them2_predicted	291135	0.868079832	4.59E-07
2650010	RGD1306143_predicted	287110	0.866240023	3.98E-08
6650349	Aqp9	65054	0.859354182	1.54E-09
3290647	RGD1565566_predicted	499057	0.851209603	4.33E-08
2570288	Olr818_predicted	405133	0.851094872	3.09E-10
6770053	Col8a1_predicted	304021	0.84817078	7.31E-08
2760494	LOC498268	498268	0.845057915	1.04E-07
940731	Gramd1b_predicted	300644	0.844669703	1.60E-11
3990113	Dhx9_predicted	304859	0.844587422	6.59E-06
2060100	Fgf12	170630	0.839298556	0.043727454
6180370	S100a13_predicted	295213	0.838711838	9.05E-09
510433	Ttc3_predicted	360702	0.831166349	3.84E-07
3290128	Galnt13	311039	0.830018329	4.52E-07
4120152	Il17rb_predicted	306247	0.829435307	4.76E-10
3140095	Npy	24604	0.826212099	0.002515032
4120136	RGD1561229_predicted	502492	0.825956121	1.75E-09
4540484	MGC109194	500400	0.825280141	8.11E-11
520110	LOC686988	686988	0.824954371	2.33E-10
4230673	Bmp3	25667	0.823429606	7.51E-11
4200014	Ctla4	63835	0.822630235	5.53E-09
5360647	LOC299264	299264	0.821125733	5.17E-07
4730706	Nsd1_predicted	306764	0.817058914	3.61E-09
6590497	Pik3ap1_predicted*	294048	0.811603838	1.64E-05
460136	Cd47	29364	0.804416358	5.77E-07
6040603	Nbn	85482	0.799058694	3.06E-09
2100504	Usp48	362636	0.798861188	3.31E-07
5670019	Acox2	252898	0.796564185	2.21E-09
3990133	Aer61	494219	0.793992183	1.09E-09
6860735	Tmem5	299841	0.793877917	1.44E-10
5360092	RGD1306959_predicted*	361624	0.793581277	0.001069814
870128	LOC503070	503070	0.791089236	5.77E-08
6770204	Gpc4	317322	0.781419108	0.000406369
**6900736**	**Tpcn2_predicted***	**309139**	**0.781366381**	**2.95E-08**

360309	RGD1565165_predicted	501604	0.781235149	2.20E-08
4610593	Fbxo11	301674	0.780756344	4.36E-08
510286	Plekha4	308584	0.77700709	5.00E-10
4760239	Rtp4_predicted	360733	0.775287323	0.001230945
4050537	RGD1563580_predicted	500244	0.774370864	3.08E-11
2640070	Btbd14a	296583	0.772746224	3.07E-09
2470129	LOC304860	304860	0.772464721	5.52E-08
510735	Slc1a3	29483	0.768436375	5.79E-06
3990239	Mlc3	56781	0.767204118	3.69E-10
1340066	MGC72614	310540	0.767124816	4.40E-06
1090154	Oas1k	494198	0.761517919	0.018476595
3440068	RGD1309085_predicted	297821	0.761197711	8.53E-08
5910441	Znhit2_predicted	309177	0.761056916	4.03E-08
6860593	LOC498786	498786	0.760305326	1.02E-09
380458	LOC501280	501280	0.756988372	5.90E-10
730301	RGD1563880_predicted	503478	0.75521686	3.59E-09
5130164	LOC502668	502668	0.75230009	4.85E-11
3870309	Vdac3	83532	0.748674709	2.50E-10
1170463	RGD1564295_predicted	317585	0.743816028	2.31E-11
4920451	Col4a2_predicted	306628	0.74169335	2.51E-09
3120671	Ogt	26295	0.735524516	6.13E-08
2030044	RGD1309189_predicted	310787	0.733269034	1.41E-08
5690519	RGD1564924_predicted	500267	0.731965498	0.000613341
1850368	Cs	170587	0.731591237	1.02E-07
1170494	Kcnj8	25472	0.728923203	9.21E-11
6840170	RGD1560514_predicted	498757	0.726101256	8.75E-09
1580452	RGD1564048_predicted	499917	0.725900861	4.56E-08
7000136	RGD1564089_predicted	314304	0.724469207	6.18E-08
4070288	Freq	65153	0.723718318	3.76E-07
430170	LOC501064	501064	0.723289564	4.08E-08
7050451	Ing4	297597	0.721127743	3.91E-11
460411	Hexb	294673	0.717939131	0.000283343
7040435	Slc9a3*	24784	0.717619124	3.24E-05
7000068	Csdc2	266600	0.715804332	0.003065751
4070670	Gnb3	60449	0.712925977	3.36E-08
6900035	Igfbp3	24484	0.711843813	0.001981135
4810047	LOC302210	302210	0.710223176	2.18E-08
7100131	Ppm1a	24666	0.70757459	1.05E-09
4920278	Lypla2*	83510	0.697798128	6.90E-11
5700592	LOC687292	687292	0.69720367	3.07E-09
6900725	LOC500297	500297	0.694825817	0.000781031
5700382	RGD1560328_predicted	499317	0.693680883	2.17E-09
3850504	Lgals5	25475	0.691673569	3.00E-05
1770100	Ccr3	117027	0.690512997	3.21E-08
4540463	Kcnmb2	294961	0.6866632	5.34E-08
1770338	Cilp_predicted	315761	0.686079229	0.000126308
450079	Socs7_predicted	287659	0.685048268	2.74E-07
6770132	RGD1566120_predicted	497904	0.681631632	1.49E-07
2100059	Olr753_predicted	405237	0.680475537	4.28E-07
1580494	Cd34_predicted	305081	0.679875955	1.33E-07
5670746	Btnl8	406160	0.677330028	5.56E-10
6650131	Pfkfb1	24638	0.672699091	0.00012236
3850324	Trim14_predicted	313236	0.669824083	3.53E-08
6200452	Lig4_predicted	290907	0.660736649	3.76E-09
450148	RGD1311307	361238	0.655609815	0.015197414
3440040	LOC316130	316130	0.655103565	0.001779342
1990369	RGD1305283_predicted	363162	0.654030665	3.10E-09
1500068	Gda*	83585	0.652976047	0.028895211

4560278	Pmm1	300089	0.652253447	4.25E-08
870497	RGD1309228	298851	0.651486301	1.48E-08
6100128	Slc35e1_predicted	306328	0.651430693	2.25E-08
3130075	Zmynd10	363139	0.646301305	7.66E-08
1580010	Rab6b_predicted	363123	0.64180891	1.05E-08
430113	Hspb3	78951	0.64100089	1.84E-06
430128	RGD1561042_predicted	498386	0.63884434	3.39E-07
2630129	RGD1566029_predicted	500913	0.638274493	5.43E-10
5860075	LOC681618	681618	0.637458524	1.26E-06
1570132	Mx1	24575	0.636320507	0.019535617
6420131	Mgst3_predicted	289197	0.634711836	5.22E-07
6450025	Prpf40a_predicted	295607	0.63329763	9.30E-06
1660685	Gm2a	282838	0.633190267	8.21E-11
4730725	LOC297968	297968	0.630025814	3.97E-09
2370341	Tbx15_predicted	295315	0.629456644	0.022886502
5360097	Hebp1_predicted	362454	0.629309212	4.44E-10
2510092	RGD1562402_predicted	499133	0.625579621	7.23E-06
4540095	Crb3	301112	0.624781154	6.33E-06
5860093	Nudt7_predicted	361413	0.62239831	0.001020999
6550707	Zbtb7b_predicted	295248	0.620574492	2.18E-08
1660735	Blnk*	499356	0.618978225	2.02E-10
4210204	RGD1565969_predicted	361261	0.617309536	8.79E-09
6350110	Zyx	114636	0.617166188	2.06E-07
5340451	Nedd1_predicted	299730	0.613868203	2.40E-07
2690605	RGD1560559_predicted	303817	0.610430331	2.88E-09
1990471	Igfbp7	289560	0.608756407	0.00040373
150520	Mycn	298894	0.608619058	0.00030805
1570671	RGD1564507_predicted	363974	0.608114039	2.01E-07
4850010	Yars2	287924	0.608045863	0.001850105
580408	Svil_predicted	361256	0.604509404	0.001872441
3190129	RGD1564725_predicted	361174	0.604045147	5.00E-09
1090195	LOC685888	685888	0.603408788	2.14E-06
60400	RGD1560538_predicted	291844	0.602088777	6.92E-05
870022	RGD1306000_predicted	363611	0.601462277	6.85E-08
3440041	Tnnc2	296369	0.601383746	2.49E-06
630333	Tctex1	83462	0.598748543	1.01E-08
5130279	Hrh3	85268	0.592134894	5.12E-08
50731	Cyp11a1	29680	0.587689479	0.001147393
6180451	RGD1311783_predicted*	294012	0.585369041	0.000602294

* transcripts with differential expression between SHRSP and F344 as well as between SHRSP and consomic SHSRP-1^{F344}; transcripts mapping within the LVH QTL interval on RNO1 between genetic markers D1Rat60 to D1Rat71 in bold
