# Supplementary material for: Cpxm2 as a novel candidate for cardiac hypertrophy and failure in hypertension
Source: Hypertens Res. 2021 Dec 16;45(2):292–307. doi: 10.1038/s41440-021-00826-8 (PMC8766285; doi:10.1038/s41440-021-00826-8)
Supplement: Supplementary file 4 — Supplementary Table 3 [file 41440_2021_826_MOESM4_ESM.pdf]

Supplementary Table 3. Microarray results SHRSP vs. SHRSP-1^{F344}
The full data sets from microarray results are available via GEO accession number GSE154327.

PROBE_ID	SYMBOL	RNO	Position	ENTREZ_GENE_ID	logFC	adj.P.Val
1990048	Maoa	X	6554698-6620722	29253	0.626586829	0.012482078
580239	Picalm*	1	154377247-154458425	89816	1.195350238	6.71E-12
6760239	Clns1a*	1	162676269-162696544	65160	0.742225548	2.37E-08
4570142	Ucp2*	1	165506361-165512744	54315	0.90147734	5.99E-07
6400253	Mrpl48_predicted*	1	165563788-165606375	293149	0.982335744	6.77E-10
6550528	Art1_predicted	1	167202064-167206615	308873	0.659525519	0.004571152
4610273	MGC72973*	1	168945531-168953023	361619	1.910247366	3.01E-06
3140068	Tpp1	1	170588035-170594168	83534	0.731535661	1.26E-06
3130670	Mrpl17*	1	170652294-170653920	171061	-2.289800604	4.79E-17
5360092	RGD1306959_predicted (Akip1)*	1	174330628-174339494	361624	0.673238632	0.037315182
**5570040**	**Cpxm2_predicted***	**1**	**204048785-204161090**	**293566**	**-0.876937203**	**6.50E-08**
**2230576**	**Bnip3***	**1**	**211248098-211265282**	**84480**	**1.871184919**	**6.23E-09**
**510408**	**Slc22a18***	**1**	**216677373-216700641**	**309131**	**0.910133089**	**2.09E-07**
**6900736**	**Tpcn2_predicted***	**1**	**218419182-218448902**	**309139**	**-0.767678123**	**4.92E-07**
**4850673**	**RGD1304686 (Eif1ad)***	**1**	**220746387-220751687**	**293673**	**-0.836379601**	**1.77E-11**
**2060035**	**Pola2**	**1**	**221273538-221321570**	**85242**	**-0.89378895**	**3.17E-05**
2340079	Aldh1a1*	1	238222521-238264330	24188	-0.817139572	5.13E-05
1500068	Gda*	1	238982392-239057732	83585	-1.032856772	0.00382334
4810358	Mamdc2	1	241071110-241223798	309410	0.606849673	0.005810741
2030079	Vldlr*	1	245237736-245269205	25696	1.295438266	2.64E-06
1660735	Blnk*	1	260186967-260254600	499356	-0.586928774	5.91E-09
6590497	Pik3ap1_predicted*	1	260527344-260679132	294048	-0.789243279	0.000227075
3450500	Bloc1s2*	1	263913854-263920962	293938	0.852116261	5.91E-09
6180451	RGD1311783_predicted*	1	266451021-266464903	294012	-0.774768042	0.000142357
580162	Usmg5*	1	266859961-266866753	171069	1.791045126	3.66E-10
7040435	Slc9a3*	1	31734327-31777070	24784	-0.742864203	0.000182765
1660403	Mrpl18_predicted*	1	48033531-48038726	292244	4.397582601	1.40E-24
1410056	Rps16*	1	85405512-85408444	140655	-0.984339739	1.92E-10
1850022	Polr2i_predicted*	1	88686812-88688249	292778	0.977903977	2.14E-11
2850368	F3	2	225310624-225322272	25584	-0.777690497	0.037974465
6100722	Edg7_predicted	2	252090634-252159221	360355	-0.832269195	6.12E-07
780400	Cited4	5	139597731-139598622	114491	0.597870566	0.016338136
2570279	Hmgcl	5	154294806-154308640	79238	-0.936428122	0.000129629
4920278	Lypla2*	5	154315033-154319629	83510	-0.787870922	6.94E-11
4560170	RGD1308048_predicted*	5	154322562-154333080	298557	-0.642027071	0.000973133
1740014	Cda_predicted	5	156703579-156734541	362638	0.596336263	0.000302229
450397	Cd63*	7	3320103-3335582	29186	-1.251620641	0.001181263
6420735	Abra	7	80792600-80796670	286965	-0.986718581	0.014244553
4120021	RGD1565371_predicted*	9	53616224-53617837	501140	4.326344936	9.79E-19
2360358	Btg2	13	50913180-50916982	29619	-0.753912845	0.022335627
5700129	LOC502176	18	60745035-60767280	502176	0.727145794	0.002416997

* transcripts with differential expression between SHRSP and F344 as well as between SHRSP and consomic SHSRP-1^{F344};
transcripts mapping within the LVH QTL interval on RNO1 between genetic markers D1Rat60 to D1Rat71 in bold
