# Supplementary material for: Cpxm2 as a novel candidate for cardiac hypertrophy and failure in hypertension
Source: Hypertens Res. 2021 Dec 16;45(2):292–307. doi: 10.1038/s41440-021-00826-8 (PMC8766285; doi:10.1038/s41440-021-00826-8)
Supplement: Supplementary file 5 — Supplementary Table 4 [file 41440_2021_826_MOESM5_ESM.pdf]

Supplementary Table 4. Microarray results WT SHAM vs. WT DOCA
The full data sets from microarray results are available via GEO accession number GSE154327.

ID	Fold Change	FDR P-val	Gene Symbol	Description
17500662	-3.12	0.0052	Zdhhc2	zinc finger, DHHC domain containing 2
17516777	-3.09	0.0052	Fxyd6	FXYD domain-containing ion transport regulator 6
17468573	-1.98	0.0052	Antxr1	anthrax toxin receptor 1
17217373	-3.32	0.0052	Fmod	fibromodulin
17503825	-2.12	0.0052	Mmp2	matrix metallopeptidase 2
17221158	-2.95	0.0052	Mybl1	myeloblastosis oncogene-like 1
17294934	-12.95	0.0052	Thbs4	thrombospondin 4
17325904	-2.27	0.0052	Ccdc80	coiled-coil domain containing 80
17495191	-2.57	0.0052	Dkk3	dickkopf homolog 3 (Xenopus laevis)
17501919	-8.58	0.0052	Comp	cartilage oligomeric matrix protein
17508722	-1.93	0.0052	Leprotl1	leptin receptor overlapping transcript-like 1
17292390	2.13	0.0052	Wnk2	WNK lysine deficient protein kinase 2
17520425	-1.86	0.0052	Pcolce2	procollagen C-endopeptidase enhancer 2
17227828	-2.57	0.0052	Pla2g4a	phospholipase A2, group IVA (cytosolic, calcium-dependent)
17519394	-1.77	0.0052	Myo5a	myosin VA
17547719	-2.34	0.0052	Ahnak2	AHNAK nucleoprotein 2
17367216	-1.95	0.0052	Plxdc2	plexin domain containing 2
17387305	-4.56	0.0052	Frzb	frizzled-related protein
17287160	-3.12	0.0052	Aspn	asporin
17398233	-1.95	0.0052	Smc4	structural maintenance of chromosomes 4
17320583	-1.89	0.0052	Cpne8	copine VIII
17293469	2.83	0.0052	Gm23739	predicted gene, 23739 [Source:MGI Symbol;Acc:MGI:5453516]
17513995	-4.85	0.0052	Acta1	actin, alpha 1, skeletal muscle
17472192	-2.75	0.0062	Mgp	matrix Gla protein
17510114	-2.18	0.0062	Gdf15	growth differentiation factor 15
17422732	-1.61	0.0067	Mxra8	matrix-remodelling associated 8
17240181	-1.95	0.0067	Fam26e	family with sequence similarity 26, member E
17280125	-2.34	0.0067	Pqlc3	PQ loop repeat containing
17335506	-2.43	0.0067	Pi16	peptidase inhibitor 16
17459423	-2.82	0.0067	Igkv6-23; Igkv3-4; Igk-V8; Igk-V21; Igkc; Igk-V28	immunoglobulin kappa variable 6-23; immunoglobulin kappa variable 3-4; immunoglobulin kappa chain variable 8 (V8); immunoglobulin kappa chain variable 21 (V21); immunoglobulin kappa constant; immunoglobulin kappa chain variable 28 (V28)
17227892	-3.09	0.0067	Prg4	proteoglycan 4 (megakaryocyte stimulating factor, articular superficial zone protein)
17433815	-1.67	0.0067	Mmp23	matrix metallopeptidase 23
17287170	-2.11	0.0067	Omd	osteomodulin
17347355	-1.73	0.0067	Eif2ak2	eukaryotic translation initiation factor 2-alpha kinase 2
17496345	-2.6	0.0067	Nupr1	nuclear protein transcription regulator 1
17309482	-1.75	0.0067	Uggt2	UDP-glucose glycoprotein glucosyltransferase 2
17531999	-1.76	0.0067	Rbms3	RNA binding motif, single stranded interacting protein
17481550	-2.18	0.0067	Olfml1	olfactomedin-like 1
17500735	-2.08	0.0067	Pdgfrl	platelet-derived growth factor receptor-like
17241731	-1.56	0.0067	Bicc1	bicaudal C homolog 1 (Drosophila)
17457876	2.09	0.0067	Gstk1	glutathione S-transferase kappa 1
17311551	-3.21	0.0067	Col14a1	collagen, type XIV, alpha 1
17364192	-2.03	0.0067	1500017E21Rik	RIKEN cDNA 1500017E21 gene
17518512	-14.23	0.0067	Cilp	cartilage intermediate layer protein, nucleotide pyrophosphohydrolase
17287148	-1.78	0.0067	Ecm2	extracellular matrix protein 2, female organ and adipocyte specific
17247948	3.24	0.0067	Efemp1	epidermal growth factor-containing fibulin-like extracellular matrix protein 1
17250839	-1.9	0.0067	Pmp22	peripheral myelin protein 22
17356383	2.32	0.0067	Gal3st3	galactose-3-O-sulfotransferase 3
17269974	-4.13	0.0067	Meox1	mesenchyme homeobox 1
17539611	-2.02	0.0067	Gpm6b	glycoprotein m6b
17334406	2.39	0.0067	Rpl3l	ribosomal protein L3-like
17240665	-6.15	0.0067	Gm26016	predicted gene, 26016 [Source:MGI Symbol;Acc:MGI:5455793]
17503446	1.84	0.0067	Gpt2	glutamic pyruvate transaminase (alanine aminotransferase) 2
17221014	-1.78	0.0067	Cd34	CD34 antigen
17464040	1.63	0.0067	Pde3a	phosphodiesterase 3A, cGMP inhibited
17279230	1.97	0.0067	Zfyve21	zinc finger, FYVE domain containing 21
17254128	1.74	0.0067	Unc45b	unc-45 homolog B (C. elegans)
17318428	1.97	0.0067	Oplah	5-oxoprolinase (ATP-hydrolysing)
17430997	1.74	0.0067	Wdtc1	WD and tetratricopeptide repeats 1
17284037	1.65	0.0067	Mpc1; Mpc1-ps	mitochondrial pyruvate carrier 1; mitochondrial pyruvate carrier 1, pseudogene
17309981	-1.83	0.0067	Fyb	FYN binding protein
17309268	-1.95	0.0067	Ednrb	endothelin receptor type B
17361032	1.79	0.0067	Ndufs8	NADH dehydrogenase (ubiquinone) Fe-S protein 8
17405489	-1.99	0.0067	Igsf10	immunoglobulin superfamily, member 10
17399176	2.12	0.0069	5830417I10Rik	gon-4-like pseudogene
17523659	-1.69	0.0069	Ccr5	chemokine (C-C motif) receptor 5
17451649	2.13	0.0069	Acads	acyl-Coenzyme A dehydrogenase, short chain
17272877	1.98	0.007	Tbc1d16	TBC1 domain family, member 16
17509315	-1.91	0.007	Tenm3	teneurin transmembrane protein 3
17271244	-1.54	0.0071	Slc16a6	solute carrier family 16 (monocarboxylic acid transporters), member 6
17338529	-1.96	0.0071	Gm7334; Btg3	B-cell translocation gene 3 pseudogene; B cell translocation gene 3
17458393	-1.85	0.0073	Tmem176a	transmembrane protein 176A
17508609	-2.34	0.0074	Nrg1	neuregulin 1
17388733	-1.92	0.0074	Cd44	CD44 antigen
17524264	-1.85	0.0074	Naalad2	N-acetylated alpha-linked acidic dipeptidase 2
17351634	-1.81	0.0074	Ccdc68	coiled-coil domain containing 68
17444970	-1.91	0.0074	Medag	mesenteric estrogen dependent adipogenesis
17503884	-2.17	0.0074	Gnao1	guanine nucleotide binding protein, alpha O
17514447	-3.01	0.0074	Casp12	caspase 12
17364176	-2.06	0.0074	Ankrd1	ankyrin repeat domain 1 (cardiac muscle)
17477116	2	0.0076	Etfb	electron transferring flavoprotein, beta polypeptide
17500922	-2.06	0.0077	Pdlim3	PDZ and LIM domain 3
17511693	3.99	0.0077	Ces1d	carboxylesterase 1D
17459870	-2.7	0.0077	Loxl3	lysyl oxidase-like 3
17282570	-3.96	0.0077	Ltbp2	latent transforming growth factor beta binding protein 2
17410390	1.69	0.0077	Hadh	hydroxyacyl-Coenzyme A dehydrogenase
17479575	-2.09	0.0077	Prc1	protein regulator of cytokinesis 1
17520624	-2.75	0.0077	Rbp1	retinol binding protein 1, cellular
17524969	-1.7	0.0077	Anln	anillin, actin binding protein
17257874	-1.59	0.0077	Prkar1a	protein kinase, cAMP dependent regulatory, type I, alpha
17277939	2.1	0.0077	9030617O03Rik; Gm36572	RIKEN cDNA 9030617O03 gene; predicted gene, 36572
17350078	-3.09	0.0077	2900055J20Rik	RIKEN cDNA 2900055J20 gene
17227696	-2.13	0.0077	Cfh	complement component factor h
17275524	-1.68	0.0077	Srp54c	signal recognition particle 54C
17394036	1.63	0.0077	Fitm2	fat storage-inducing transmembrane protein 2
17355435	2.48	0.0077	Scarna17	small Cajal body-specific RNA 17
17497612	1.52	0.0077	Echs1	enoyl Coenzyme A hydratase, short chain, 1, mitochondrial
17305709	-2.27	0.0077	Bmp4	bone morphogenetic protein 4
17462437	-1.62	0.0077	Usp18	ubiquitin specific peptidase 18
17240357	-1.45	0.0077	Gtf3c6	general transcription factor IIIC, polypeptide 6, alpha
17466783	-1.83	0.0077	Skap2	src family associated phosphoprotein 2
17337955	1.73	0.0077	Mrpl14	mitochondrial ribosomal protein L14
17346136	1.85	0.0077	Plin5	perilipin 5
17292157	1.61	0.0077	Dtnbp1	dystrobrevin binding protein 1
17222564	1.98	0.0077	Snord89	small nucleolar RNA, C/D box 89
17549432	1.75	0.0077		
17312405	1.49	0.0077	Maf1	MAF1 homolog (S. cerevisiae)
17373680	-2.69	0.0078	Pamr1; Gm39891	peptidase domain containing associated with muscle regeneration 1; predicted gene, 39891
17279608	-1.82	0.0082	Ncapg2	non-SMC condensin II complex, subunit G2
17245923	-1.51	0.0082	Lrp1	low density lipoprotein receptor-related protein 1
17415973	-1.7	0.0082	Leprot	leptin receptor overlapping transcript
17395165	2.21	0.0082	Atp5e	ATP synthase, H+ transporting, mitochondrial F1 complex, epsilon subunit
17469636	-1.64	0.0082	Rad18	RAD18 E3 ubiquitin protein ligase
17513590	1.86	0.0084	Fbxo31	F-box protein 31
17227536	-1.67	0.0084	Ptprc	protein tyrosine phosphatase, receptor type, C
17428801	1.99	0.0084	Snord55	small nucleolar RNA, C/D box 55
17224015	1.82	0.0085	Abca12	ATP-binding cassette, sub-family A (ABC1), member 12
17408960	-1.84	0.0085	Cd53	CD53 antigen
17358838	-1.51	0.0086	Kif20b	kinesin family member 20B
17468143	-1.7	0.0086	Mthfd2	methylenetetrahydrofolate dehydrogenase (NAD+ dependent), methenyltetrahydrofolate cyclohydrolase
17462663	-3.28	0.0086	Mfap5	microfibrillar associated protein 5
17508775	1.87	0.0086	Gm16793	predicted gene, 16793
17479974	-3.37	0.0086	Nox4	NADPH oxidase 4
17541404	-1.49	0.0086	Elf4	E74-like factor 4 (ets domain transcription factor)
17239040	-2.04	0.0086	Ulbp1	UL16 binding protein 1
17312567	2.03	0.0086	Gpt	glutamic pyruvic transaminase, soluble
17399164	2.02	0.0086	Rit1	Ras-like without CAAX 1
17211198	-2.25	0.0086	Sulf1	sulfatase 1
17227077	-1.97	0.0086	Prelp	proline arginine-rich end leucine-rich repeat
17329454	-2.45	0.0086	P3h2	prolyl 3-hydroxylase 2
17266590	-1.47	0.0086	Evi2a; Evi2b; Gm21975	ecotropic viral integration site 2a; ecotropic viral integration site 2b; predicted gene 21975
17487533	2.14	0.0086	Vmn1r103	vomeronasal 1 receptor 103
17284222	-1.75	0.0086	Ahnak2	AHNAK nucleoprotein 2
17483007	1.64	0.0086	Sbk1	SH3-binding kinase 1
17302936	-4.38	0.0086	Itgbl1	integrin, beta-like 1
17226969	1.65	0.0086	Gm19461	predicted gene, 19461
17242416	1.69	0.0086	D10Jhu81e	DNA segment, Chr 10, Johns Hopkins University 81 expressed
17410410	-1.85	0.0086	Sgms2	sphingomyelin synthase 2
17329759	-2.61	0.0086	Apod	apolipoprotein D
17244864	-1.83	0.0086	Nav3	neuron navigator 3
17530967	1.8	0.009	Slc38a3	solute carrier family 38, member 3
17500068	-2.75	0.009	Sfrp1	secreted frizzled-related protein 1
17376270	2.93	0.009	Snord110	small nucleolar RNA, C/D box 110
17215820	1.45	0.009	Gpc1	glypican 1
17348812	1.86	0.009	Dsg2	desmoglein 2
17299268	-1.69	0.009	Cdkn3	cyclin-dependent kinase inhibitor 3
17371374	-1.46	0.009	Cers6	ceramide synthase 6
17287536	-2.1	0.009	Tspan17	tetraspanin 17
17331162	-3.24	0.009	Col8a1	collagen, type VIII, alpha 1
17509976	2.64	0.0091	Slc25a42	solute carrier family 25, member 42
17248019	-1.77	0.0091	Ccdc88a	coiled coil domain containing 88A
17548117	1.82	0.0093	Gm6293	predicted pseudogene 6293 [Source:MGI Symbol;Acc:MGI:3649011]
17527661	-1.96	0.0093	Islr	immunoglobulin superfamily containing leucine-rich repeat
17289527	-1.79	0.0093	Cd180	CD180 antigen
17327613	-1.9	0.0093	Nlrc3	NLR family, CARD domain containing 3
17346125	1.84	0.0093	Plin4	perilipin 4
17334302	1.73	0.0093	Snord60; Rab26os	small nucleolar RNA, C/D box 60 [Source:MGI Symbol;Acc:MGI:5453296]; RAB26, member RAS oncogene family, opposite strand [Source:MGI Symbol;Acc:MGI:1922864]
17378337	2.01	0.0093	Map1lc3a	microtubule-associated protein 1 light chain 3 alpha
17304406	-1.59	0.0094	Prkcd	protein kinase C, delta
17420777	1.92	0.0095	Aldh4a1	aldehyde dehydrogenase 4 family, member A1
17378540	1.65	0.0095	Romo1	reactive oxygen species modulator 1
17231163	-1.85	0.0095	Sertad4	SERTA domain containing 4
17219643	-1.74	0.0096	Aim2	absent in melanoma 2
17226142	-1.88	0.0096	Dsel	dermatan sulfate epimerase-like
17528247	1.58	0.0096	Kbtbd13	kelch repeat and BTB (POZ) domain containing 13
17342414	1.57	0.0096	Rhot2	ras homolog gene family, member T2
17504293	1.88	0.0096	Mmp15	matrix metallopeptidase 15
17375327	-1.86	0.0096	Casc4	cancer susceptibility candidate 4
17279499	1.56	0.0096	Crip2	cysteine rich protein 2
17246674	1.67	0.0097	Dusp18	dual specificity phosphatase 18
17229403	1.78	0.0098	Mgst3	microsomal glutathione S-transferase 3
17275069	-1.91	0.0099	Agmo	alkylglycerol monooxygenase
17258287	1.77	0.0099	Tmem94	transmembrane protein 94
17212199	-1.98	0.0099	Il1rl2	interleukin 1 receptor-like 2
17254508	1.6	0.0099	Car4	carbonic anhydrase 4
17543365	-3.05	0.0099	Vsig4	V-set and immunoglobulin domain containing 4
17502233	2.79	0.0099	Slc27a1	solute carrier family 27 (fatty acid transporter), member 1
17510264	-1.93	0.0099	Haus8	4HAUS augmin-like complex, subunit 8
17267066	2.24	0.0099	Dhrs11	dehydrogenase/reductase (SDR family) member 11
17321768	-1.76	0.0099	Bin2	bridging integrator 2
17529099	-6.54	0.0101	Col12a1	collagen, type XII, alpha 1
17295130	-1.94	0.0103	F2r	coagulation factor II (thrombin) receptor
17489508	-1.69	0.0104	Gm5329	predicted gene 5329 [Source:MGI Symbol;Acc:MGI:3647426]
17535644	1.76	0.0104	Plxnb3	plexin B3
17409217	-1.52	0.0104	Gnai3	guanine nucleotide binding protein (G protein), alpha inhibiting 3
17211572	-1.55	0.0105	Rab23	RAB23, member RAS oncogene family
17543870	-2.28	0.0105	Zdhhc15	zinc finger, DHHC domain containing 15
17301594	-2.84	0.0105	Stc1	stanniocalcin 1
17235053	1.61	0.0106	Wdr18	WD repeat domain 18
17453533	2.01	0.0106	Gm25492	predicted gene, 25492 [Source:MGI Symbol;Acc:MGI:5455269]
17515106	1.65	0.0107	Pde4a	phosphodiesterase 4A, cAMP specific
17480675	-2.28	0.0107	P4ha3	procollagen-proline, 2-oxoglutarate 4-dioxygenase (proline 4-hydroxylase), alpha polypeptide III
17425631	-2.34	0.0107	Svep1	sushi, von Willebrand factor type A, EGF and pentraxin domain containing 1
17262065	1.52	0.0107	Cyfip2	cytoplasmic FMR1 interacting protein 2
17243022	1.57	0.0107	Btbd2	BTB (POZ) domain containing 2
17341743	1.57	0.0107	Tbc1d24	TBC1 domain family, member 24
17370883	2.06	0.0107	Kcnj3	potassium inwardly-rectifying channel, subfamily J, member 3
17265129	1.68	0.0107	Acadvl	acyl-Coenzyme A dehydrogenase, very long chain
17548264	-1.79	0.0107		
17342689	1.59	0.0107	Uqcc2	ubiquinol-cytochrome c reductase complex assembly factor 2

17363407	-2.2	0.0108	Anxa1	annexin A1
17243544	1.96	0.0108	Aldh1l2	aldehyde dehydrogenase 1 family, member L2
17300390	2.12	0.0108	Mhrt	myosin heavy chain associated RNA transcript
17387517	-1.55	0.0108	Serping1	serine (or cysteine) peptidase inhibitor, clade G, member 1
17426306	1.96	0.0108	Whrn	whirlin
17392660	-1.52	0.0109	Zfp442; 3300002I08Rik	zinc finger protein 442; RIKEN cDNA 3300002I08 gene
17374833	-1.45	0.0109	Nusap1	nucleolar and spindle associated protein 1
17470580	-1.81	0.011	Apobec1	apolipoprotein B mRNA editing enzyme, catalytic polypeptide 1
17418168	1.95	0.011	Nt5c1a	5-nucleotidase, cytosolic IA
17310187	1.62	0.0111	Nadk2	NAD kinase 2, mitochondrial
17230830	-2.55	0.0113	Tgfb2	transforming growth factor, beta 2
17407956	1.49	0.0114	Car14	carbonic anhydrase 14
17236288	-1.56	0.0115	Igf1	insulin-like growth factor 1
17418945	1.66	0.0117	Fndc5	fibronectin type III domain containing 5
17389157	-3.87	0.0117	Fibin	fin bud initiation factor homolog (zebrafish)
17460826	2.07	0.0117	Klf15	Kruppel-like factor 15
17217639	1.58	0.0117	Lad1	ladinin
17480312	-1.77	0.0118	Gab2	growth factor receptor bound protein 2-associated protein 2
17432976	-3.4	0.0119	Angptl7	angiopoietin-like 7
17397575	-7.86	0.0122	Postn	periostin, osteoblast specific factor
17262650	1.95	0.0122	Uqcrq	ubiquinol-cytochrome c reductase, complex III subunit VII
17336896	-1.43	0.0124	Clic1	chloride intracellular channel 1
17355304	-1.44	0.0126	4930503L19Rik	RIKEN cDNA 4930503L19 gene
17435089	-2.23	0.0126	Fgl2	fibrinogen-like protein 2
17494020	1.65	0.0126	Art5	ADP-ribosyltransferase 5
17468551	-1.54	0.0126	Anxa4	annexin A4
17497334	-1.94	0.0126	Mki67	antigen identified by monoclonal antibody Ki 67
17464588	-1.51	0.0126	Sgce	sarcoglycan, epsilon
17398634	-1.76	0.0126	Sh3d19	SH3 domain protein D19
17349926	-1.58	0.0126	Pcdhb17	protocadherin beta 17
17432256	1.9	0.0126	Slc25a34	solute carrier family 25, member 34
17547978	1.82	0.0126	LOC102638850; Gm5963	40S ribosomal protein S21 pseudogene; predicted pseudogene 5963 [Source:MGI Symbol;Acc:MGI:3648418]
17541036	1.8	0.0126	Ndufa1	NADH dehydrogenase (ubiquinone) 1 alpha subcomplex, 1
17366670	-2.16	0.0128	Itih5	inter-alpha (globulin) inhibitor H5
17251816	1.76	0.0128	Ybx2	Y box protein 2
17519738	-2.51	0.0128	Cd109	CD109 antigen
17548236	-2.02	0.0128	LOC100041903	proteinase-activated receptor 1-like
17548924	-2.02	0.0128	Gm15433	predicted pseudogene 15433
17358905	-1.67	0.0128	Hectd2	HECT domain containing 2
17217969	-2.07	0.0128	Kcnt2	potassium channel, subfamily T, member 2
17298556	1.7	0.0128	Ogdhl	oxoglutarate dehydrogenase-like
17230231	-1.64	0.0128	Akt3	thymoma viral proto-oncogene 3
17397990	2.18	0.0129	Mme	membrane metallo endopeptidase
17531954	1.82	0.0129	Rps27rt; Rps27	ribosomal protein S27, retrogene; ribosomal protein S27
17541917	1.59	0.0129	Snord61	small nucleolar RNA, C/D box 61
17395512	1.88	0.0129	Lama5	laminin, alpha 5
17298775	-1.49	0.0133	Anxa8	annexin A8
17398274	1.4	0.0133	Ppm1l	protein phosphatase 1 (formerly 2C)-like
17265958	-2.18	0.0133	Serpinf1	serine (or cysteine) peptidase inhibitor, clade F, member 1
17336322	1.43	0.0133	Rxrb	retinoid X receptor beta
17475787	1.65	0.0133	Dyrk1b	dual-specificity tyrosine-(Y)-phosphorylation regulated kinase 1b
17464235	1.55	0.0133	Gm15543	predicted gene 15543
17521996	1.41	0.0133	Uqcrc1	ubiquinol-cytochrome c reductase core protein 1
17291275	-1.49	0.0133	Lrrc16a	leucine rich repeat containing 16A
17225929	-1.53	0.0133	Pam	peptidylglycine alpha-amidating monooxygenase
17302161	1.54	0.0133	Vwa8	von Willebrand factor A domain containing 8
17357486	1.73	0.0133	Syt7	synaptotagmin VII
17504146	1.49	0.0133	Coq9	coenzyme Q9 homolog (yeast)
17387276	-1.63	0.0134	Pde1a	phosphodiesterase 1A, calmodulin-dependent
17306577	-4.72	0.0134	Myh7	myosin, heavy polypeptide 7, cardiac muscle, beta
17461433	-1.47	0.0134	Edem1	ER degradation enhancer, mannosidase alpha-like 1
17360386	1.48	0.0134	Rbm20	RNA binding motif protein 20
17219110	1.59	0.0138	Rxrg	retinoid X receptor gamma
17226043	-2.71	0.0138	Bcl2	B cell leukemia/lymphoma 2
17431861	-1.6	0.014	Nbl1	neuroblastoma, suppression of tumorigenicity 1
17506206	-1.64	0.014	Crispld2	cysteine-rich secretory protein LCCL domain containing 2
17351811	1.93	0.014	Acaa2	acetyl-Coenzyme A acyltransferase 2 (mitochondrial 3-oxoacyl-Coenzyme A thiolase)
17449609	-1.52	0.014	Cdkl2	cyclin-dependent kinase-like 2 (CDC2-related kinase)
17306929	1.63	0.014	Sdr39u1	short chain dehydrogenase/reductase family 39U, member 1
17260023	1.39	0.014	Uqcr10	ubiquinol-cytochrome c reductase, complex III subunit X
17423006	1.44	0.014	Uba52	ubiquitin A-52 residue ribosomal protein fusion product 1
17547728	-1.39	0.014		
17290324	-2.18	0.014	Pfkp	phosphofructokinase, platelet
17315305	-1.73	0.014	Igfbp6	insulin-like growth factor binding protein 6
17428967	1.67	0.014	Ipo13	importin 13
17548354	-1.63	0.0141	Llph-ps1	LLP homolog, pseudogene 1
17333320	2.46	0.0144	Mrgprh	MAS-related GPR, member H
17525304	2.18	0.0144	Kcnj5	potassium inwardly-rectifying channel, subfamily J, member 5
17383633	1.61	0.0144	Crat	carnitine acetyltransferase
17525524	-1.48	0.0144	Stt3a	STT3, subunit of the oligosaccharyltransferase complex, homolog A (S. cerevisiae)
17254166	-1.93	0.0144	Slfn2	schlafen 2
17387385	-1.66	0.0144	Calcrl	calcitonin receptor-like
17367536	-1.84	0.0145	Apbb1ip	amyloid beta (A4) precursor protein-binding, family B, member 1 interacting protein
17230573	-2.13	0.0146	Enah	enabled homolog (Drosophila)
17514424	-1.5	0.0146	Casp1	caspase 1
17501976	2.01	0.0146	Isyna1	myo-inositol 1-phosphate synthase A1
17378827	-1.85	0.0146	Lbp	lipopolysaccharide binding protein
17410748	-1.59	0.0147	Pdlim5	PDZ and LIM domain 5
17349542	1.51	0.0147	Gm5239	predicted pseudogene 5239 [Source:MGI Symbol;Acc:MGI:3648788]
17256632	1.58	0.0147	Wnk4	WNK lysine deficient protein kinase 4
17477832	-2.31	0.0147		
17512337	-1.65	0.0147	B3gnt9	UDP-GlcNAc:betaGal beta-1,3-N-acetylglucosaminyltransferase 9
17406979	-1.43	0.0147	Slc50a1	solute carrier family 50 (sugar transporter), member 1
17381779	-1.42	0.0147	Rsu1	Ras suppressor protein 1
17389034	-1.49	0.0147	Rcn1	reticulocalbin 1
17350267	-1.73	0.0147	Myot	myotilin
17459716	1.43	0.0147	Suclg1	succinate-CoA ligase, GDP-forming, alpha subunit
17233001	1.42	0.0148	Pdss2	prenyl (solanesyl) diphosphate synthase, subunit 2
17534899	1.55	0.0148	Smim10l2a	small integral membrane protein 10 like 2A
17282743	-1.73	0.0149	Tgfb3	transforming growth factor, beta 3
17535208	-1.87	0.0149	Fmr1	fragile X mental retardation syndrome 1
17340177	1.63	0.015	Prkce	protein kinase C, epsilon
17440130	1.8	0.015	Rpl5	ribosomal protein L5
17213295	-1.52	0.015	Nop58	NOP58 ribonucleoprotein
17287827	-1.63	0.015	Tgfbi	transforming growth factor, beta induced
17465830	-1.55	0.015	Mtpn	myotrophin
17526776	-1.74	0.015	Ncam1	neural cell adhesion molecule 1
17524724	1.41	0.015	Tmed1	transmembrane emp24 domain containing 1
17364280	-1.81	0.0151	Myof	myoferlin
17294738	-2.46	0.0152	Vcan	versican
17459676	1.6	0.0153	Retsat	retinol saturase (all trans retinol 13,14 reductase)
17456934	-1.57	0.0154	Mest	mesoderm specific transcript
17463781	-2.85	0.0154	Emp1	epithelial membrane protein 1
17301010	-1.52	0.0154	Mtmr6	myotubularin related protein 6
17478797	1.49	0.0154	Atp5l-ps1	ATP synthase, H+ transporting, mitochondrial F0 complex, subunit G, pseudogene 1
17236811	-2.04	0.0154	Lum	lumican
17307231	-1.56	0.0154	Micu2	mitochondrial calcium uptake 2
17349979	1.57	0.0154	0610009O20Rik	RIKEN cDNA 0610009O20 gene
17247695	-1.45	0.0154	Xpo1	exportin 1, CRM1 homolog (yeast)
17214166	1.43	0.0157	Pnkd	paroxysmal nonkinesiogenic dyskinesia
17231859	-1.51	0.0158	Ifngr1	interferon gamma receptor 1
17500595	1.42	0.0158	D8Ertd82e	DNA segment, Chr 8, ERATO Doi 82, expressed
17249151	1.58	0.0158	Mgat4b	mannoside acetylglucosaminyltransferase 4, isoenzyme B
17514460	-1.56	0.0158	Pdgfd	platelet-derived growth factor, D polypeptide
17330773	-1.44	0.0158	Pvrl3	poliovirus receptor-related 3
17548352	-1.58	0.0159	Gm12816	predicted gene 12816 [Source:MGI Symbol;Acc:MGI:3649628]
17353752	1.44	0.0159	Ndufa2	NADH dehydrogenase (ubiquinone) 1 alpha subcomplex, 2
17404021	1.48	0.016	Mrps28	mitochondrial ribosomal protein S28
17286354	-1.37	0.016	Serpinb6b	serine (or cysteine) peptidase inhibitor, clade B, member 6b
17397511	-1.72	0.0161	Lhfp	lipoma HMGIC fusion partner
17288442	1.45	0.0161	Irx2	Iroquois related homeobox 2 (Drosophila)
17464404	1.59	0.0161	Mrps35	mitochondrial ribosomal protein S35
17411547	1.52	0.0161	Chchd7	coiled-coil-helix-coiled-coil-helix domain containing 7
17548926	-1.95	0.0161	LOC101055758; Gm7592	component of Sp100-rs; predicted gene 7592 [Source:MGI Symbol;Acc:MGI:3644077]
17216012	-1.43	0.0161	02. Sep	septin 2
17222053	-1.54	0.0163	Bend6; Gm38471	BEN domain containing 6; predicted gene, 38471
17532367	1.89	0.0163	Hhatl	hedgehog acyltransferase-like
17523457	1.36	0.0163	Tcaim	T cell activation inhibitor, mitochondrial
17259693	2.06	0.0163	Ptchd3	patched domain containing 3
17298364	1.97	0.0163	Nt5dc2	5-nucleotidase domain containing 2
17434497	-1.4	0.0163	Sri	sorcin
17270082	1.6	0.0163	Hdac5	histone deacetylase 5
17531932	2.13	0.0164	Cmtm8	CKLF-like MARVEL transmembrane domain containing 8
17493632	1.94	0.0164	Dgat2	diacylglycerol O-acyltransferase 2
17506273	1.52	0.0164	Cox4i1	cytochrome c oxidase subunit IV isoform 1
17277140	4.6	0.0164	Acot1	acyl-CoA thioesterase 1
17374121	-1.55	0.0164	Ccdc34	coiled-coil domain containing 34
17467269	-1.58	0.0164	Hpgds	hematopoietic prostaglandin D synthase
17275490	-1.51	0.0164	1700047I17Rik2; Fam177a	RIKEN cDNA 1700047I17 gene 2; family with sequence similarity 177, member A
17275516	-1.51	0.0164	1700047I17Rik2; Fam177a	RIKEN cDNA 1700047I17 gene 2; family with sequence similarity 177, member A
17464367	-1.8	0.0165	Ppfibp1	PTPRF interacting protein, binding protein 1 (liprin beta 1)
17518191	-1.55	0.0166	Itga11	integrin alpha 11
17423030	2.61	0.0166	Penk	preproenkephalin
17266698	-1.39	0.0166	Myo1d	myosin ID
17382729	1.58	0.0166	Tmem141	transmembrane protein 141
17434585	-1.47	0.0166	Abcb4	ATP-binding cassette, sub-family B (MDR/TAP), member 4
17312191	1.59	0.0166	Them6	thioesterase superfamily member 6
17365098	-1.79	0.0167	Scd1	stearoyl-Coenzyme A desaturase 1
17423463	1.45	0.0167	Decr1	2,4-dienoyl CoA reductase 1, mitochondrial
17479099	-1.7	0.0167	Igf1r	insulin-like growth factor I receptor
17412123	-1.5	0.0168	Ccnc	cyclin C
17243525	-1.42	0.0168	Slc41a2	solute carrier family 41, member 2
17245302	-1.37	0.0168	Rap1b	RAS related protein 1b
17434742	-2.24	0.017	Sema3d	sema domain, immunoglobulin domain (Ig), short basic domain, secreted, (semaphorin) 3D
17438361	-1.39	0.017	Cep135	centrosomal protein 135
17341240	1.53	0.017	Oaz1-ps; Oaz1	ornithine decarboxylase antizyme 1, pseudogene; ornithine decarboxylase antizyme 1
17351655	-1.5	0.0171	Stard6	StAR-related lipid transfer (START) domain containing 6
17334253	1.69	0.0172	Eci1	enoyl-Coenzyme A delta isomerase 1
17306274	-1.47	0.0172	Rab2b	RAB2B, member RAS oncogene family
17431749	1.71	0.0172	Pink1	PTEN induced putative kinase 1
17409284	1.82	0.0173	Celsr2	cadherin, EGF LAG seven-pass G-type receptor 2 (flamingo homolog, Drosophila)
17362953	-1.76	0.0173	Ms4a7	membrane-spanning 4-domains, subfamily A, member 7
17542220	-1.7	0.0174	Gabra3	gamma-aminobutyric acid (GABA) A receptor, subunit alpha 3
17529417	-2.2	0.0174	Snap91	synaptosomal-associated protein 91
17357611	1.48	0.0174	Prpf19	PRP19/PSO4 pre-mRNA processing factor 19 homolog (S. cerevisiae)
17367163	1.43	0.0175	Cacnb2	calcium channel, voltage-dependent, beta 2 subunit
17533474	-1.6	0.0175	Maoa	monoamine oxidase A
17225499	1.6	0.0175	Hes6	hairy and enhancer of split 6
17357815	-1.6	0.0175	Fam111a	family with sequence similarity 111, member A
17357810	-1.76	0.0175	Mpeg1	macrophage expressed gene 1
17499962	-1.55	0.0176	Plat	plasminogen activator, tissue
17406129	-1.55	0.0176	Tmem144	transmembrane protein 144
17528405	-1.63	0.0176	Rab8b	RAB8B, member RAS oncogene family
17520851	1.36	0.0176	Anapc13	anaphase promoting complex subunit 13
17463332	-1.67	0.0176	Fgf6	fibroblast growth factor 6
17492031	-2.17	0.0177	4833412C05Rik	RIKEN cDNA 4833412C05 gene
17401269	-1.5	0.0178	Ptpn22	protein tyrosine phosphatase, non-receptor type 22 (lymphoid)
17248809	-1.43	0.0178	Havcr2	hepatitis A virus cellular receptor 2
17351833	1.84	0.0178	Gm23301	predicted gene, 23301 [Source:MGI Symbol;Acc:MGI:5453078]
17310349	1.32	0.0178	Amacr; Slc45a2	alpha-methylacyl-CoA racemase; solute carrier family 45, member 2
17286587	-1.77	0.0178	Ly86	lymphocyte antigen 86
17314061	1.52	0.0178	Trabd	TraB domain containing
17334354	1.68	0.0178	Gm24589	predicted gene, 24589 [Source:MGI Symbol;Acc:MGI:5454366]
17550064	1.68	0.0178		
17380549	1.6	0.0179	Gm14420	predicted gene 14420
17441037	-1.83	0.0179	Oasl2	2-5 oligoadenylate synthetase-like 2
17383619	1.44	0.0179	Sh3glb2	SH3-domain GRB2-like endophilin B2
17236800	-1.42	0.0179	Dcn	decorin
17458587	-1.94	0.018	Halr1	Hoxa adjacent long noncoding RNA 1
17529859	-1.51	0.018	E030011O05Rik; Zbtb38	RIKEN cDNA E030011O05 gene; zinc finger and BTB domain containing 38
17544061	-2.92	0.018	Cysltr1	cysteinyl leukotriene receptor 1
17242474	1.42	0.018	Agpat3	1-acylglycerol-3-phosphate O-acyltransferase 3
17475975	1.99	0.018	Ech1	enoyl coenzyme A hydratase 1, peroxisomal

17501994	1.37	0.018	Lsm4	LSM4 homolog, U6 small nuclear RNA associated (S. cerevisiae)
17436877	1.7	0.0181	Ablim2	actin-binding LIM protein 2
17389481	-1.68	0.0181	Arhgap11a	Rho GTPase activating protein 11A
17407412	-4.8	0.0181	Sprr1a	small proline-rich protein 1A
17523650	-1.76	0.0181	Ccr2	chemokine (C-C motif) receptor 2
17288079	-1.61	0.0181	Gm24588	predicted gene, 24588 [Source:MGI Symbol;Acc:MGI:5454365]
17495839	-1.59	0.0183	Igsf6	immunoglobulin superfamily, member 6
17274394	-1.73	0.0183		
17366201	-1.79	0.0183	LOC101055672	nuclear body protein SP140-like
17342656	1.71	0.0183	Nkx2-5	NK2 homeobox 5
17547939	1.82	0.0183	Gm4943	predicted pseudogene 4943
17447884	-1.53	0.0183	Ldb2	LIM domain binding 2
17456308	2.46	0.0183	Kcnd2	potassium voltage-gated channel, Shal-related family, member 2
17294489	-1.44	0.0183	Arsk	arylsulfatase K
17433469	1.89	0.0183	Rnf207	ring finger protein 207
17252767	1.45	0.0183	Cluh	clustered mitochondria (cluA/CLU1) homolog
17224629	1.46	0.0183	Obsl1	obscurin-like 1
17292562	1.57	0.0183	Sema4d	sema domain, immunoglobulin domain (Ig), transmembrane domain (TM) and short cytoplasmic domain, (semaphorin) 4D
17297759	1.49	0.0183	Anxa11; Gm2260; Gm2274	annexin A11; predicted gene 2260; predicted gene 2274
17239371	-1.39	0.0185	Ltv1	LTV1 homolog (S. cerevisiae)
17351511	-1.36	0.0186	Cep192	centrosomal protein 192
17264769	2.36	0.0186	Efnb3	ephrin B3
17260177	1.61	0.0186	Mrps24	mitochondrial ribosomal protein S24
17319806	-1.91	0.0186	Arfgap3	ADP-ribosylation factor GTPase activating protein 3
17291833	1.38	0.0187	Eci2	enoyl-Coenzyme A delta isomerase 2
17485963	1.92	0.0187	Sbk2	SH3-binding domain kinase family, member 2
17379314	-2.04	0.0187	Wisp2	WNT1 inducible signaling pathway protein 2
17267471	-2.15	0.0187	Ppm1e	protein phosphatase 1E (PP2C domain containing)
17332228	-3.62	0.0187	Rcan1	regulator of calcineurin 1
17301968	-1.66	0.0187	Lcp1	lymphocyte cytosolic protein 1
17446185	1.46	0.0187	Fastk	Fas-activated serine/threonine kinase
17305116	-1.34	0.0187	Ccser2	coiled-coil serine rich 2
17441202	1.37	0.0187	Gcn1l1	GCN1 general control of amino-acid synthesis 1-like 1 (yeast)
17396638	-1.64	0.0187	Skil	SKI-like
17243113	2.58	0.0187	Lingo3	leucine rich repeat and Ig domain containing 3
17542516	1.44	0.0187	Hcfc1	host cell factor C1
17400375	-2.07	0.0187	Ctss	cathepsin S
17527927	1.46	0.0187	Rplp1	ribosomal protein, large, P1
17308525	-1.41	0.0187	Fndc3a	fibronectin type III domain containing 3A
17250193	1.93	0.0189	Gm10435; LOC105244175	predicted gene 10435 [Source:MGI Symbol;Acc:MGI:3642820]; uncharacterized LOC105244175
17363598	1.4	0.0189	Fxn	frataxin
17226622	-1.54	0.0189	Cd55	CD55 molecule, decay accelerating factor for complement
17400592	1.52	0.0189	Hfe2	hemochromatosis type 2 (juvenile) (human homolog)
17212185	-1.51	0.0189	Il1r1	interleukin 1 receptor, type I
17394578	1.76	0.0189	Gm14267	predicted gene 14267 [Source:MGI Symbol;Acc:MGI:3649851]
17487982	1.87	0.0191	Bckdha	branched chain ketoacid dehydrogenase E1, alpha polypeptide
17306344	-1.48	0.0191	Slc7a7	solute carrier family 7 (cationic amino acid transporter, y+ system), member 7
17537038	1.89	0.0191	Fgf16	fibroblast growth factor 16
17537190	-1.41	0.0191	Sh3bgrl	SH3-binding domain glutamic acid-rich protein like
17535558	-1.71	0.0192	Bgn	biglycan
17464836	1.59	0.0192	Ndufa4	NADH dehydrogenase (ubiquinone) 1 alpha subcomplex, 4
17288112	2.12	0.0192	2010111I01Rik; Mir24-1	RIKEN cDNA 2010111I01 gene; microRNA 24-1
17437686	-1.48	0.0193	Fam114a1	family with sequence similarity 114, member A1
17314190	1.48	0.0193	Shank3	SH3/ankyrin domain gene 3
17444713	-1.51	0.0193	Cdk8	cyclin-dependent kinase 8
17486216	1.65	0.0193	Vmn2r32	vomeronasal 2, receptor 32
17242177	-1.62	0.0193	Col6a1	collagen, type VI, alpha 1
17449084	-1.73	0.0193	Igfbp7	insulin-like growth factor binding protein 7
17285559	-1.55	0.0193	Elmo1	engulfment and cell motility 1
17295278	-1.65	0.0193	Hexb	hexosaminidase B
17358138	-1.47	0.0193	Tmem2	transmembrane protein 2
17356874	1.32	0.0193	Pygm	muscle glycogen phosphorylase
17270193	1.35	0.0193	Slc25a39	solute carrier family 25, member 39
17383819	1.5	0.0194	Fam78a	family with sequence similarity 78, member A
17438246	-1.39	0.0194	Pdgfra	platelet derived growth factor receptor, alpha polypeptide
17434254	1.33	0.0194	Gm13306; Ccl27b; Gm2506	predicted gene 13306; chemokine (C-C motif) ligand 27b; predicted gene 2506
17530141	1.34	0.0194	Pccb	propionyl Coenzyme A carboxylase, beta polypeptide
17278328	-2.8	0.0194	Serpina3n	serine (or cysteine) peptidase inhibitor, clade A, member 3N
17476288	1.68	0.0194	Cox7a1	cytochrome c oxidase subunit VIIa 1
17398557	-2.27	0.0195	Sfrp2	secreted frizzled-related protein 2
17261840	-1.46	0.0195	Hmmr	hyaluronan mediated motility receptor (RHAMM)
17408863	1.44	0.0195	Atp5f1	ATP synthase, H+ transporting, mitochondrial F0 complex, subunit B1
17390066	-1.53	0.0196	Ehd4	EH-domain containing 4
17537003	-1.52	0.0196	Uprt	uracil phosphoribosyltransferase (FUR1) homolog (S. cerevisiae)
17522958	-1.72	0.0197	Ctdspl	CTD (carboxy-terminal domain, RNA polymerase II, polypeptide A) small phosphatase-like
17341356	-1.79	0.0197	Gm26384	predicted gene, 26384 [Source:MGI Symbol;Acc:MGI:5456161]
17539888	1.69	0.0197	Magix	MAGI family member, X-linked
17330359	-1.49	0.0201	Pla1a	phospholipase A1 member A
**17497058**	**-4.88**	**0.0201**	**Cpxm2**	**carboxypeptidase X 2 (M14 family)**
17436457	-1.35	0.0201	Tacc3	transforming, acidic coiled-coil containing protein 3
17518378	-1.46	0.0201	Dennd4a	DENN/MADD domain containing 4A
17272461	-1.71	0.0203	Cygb	cytoglobin
17330815	-1.66	0.0203	Dzip3	DAZ interacting protein 3, zinc finger
17457790	1.74	0.0203	Tcrb-J; Trbj2-1	T cell receptor beta, joining region; T cell receptor beta joining 2-1
17447089	1.45	0.0203	Ctbp1	C-terminal binding protein 1
17424631	2.72	0.0203	Rmrp	RNA component of mitochondrial RNAase P
17513076	-1.6	0.0206	Mlkl	mixed lineage kinase domain-like
17402797	-1.4	0.0208	Cenpe	centromere protein E
17269486	1.93	0.0208	P3h4	prolyl 3-hydroxylase family member 4 (non-enzymatic)
17470119	1.66	0.0208	Ret	ret proto-oncogene
17414434	-1.35	0.0208	Ugcg	UDP-glucose ceramide glucosyltransferase
17400162	1.81	0.0208	Selenbp1	selenium binding protein 1
17396009	-1.68	0.0208	Zc2hc1a	zinc finger, C2HC-type containing 1A
17310816	2.13	0.0209	Cmbl	carboxymethylenebutenolidase-like (Pseudomonas)
17271920	-1.51	0.0209	Hn1	hematological and neurological expressed sequence 1
17228057	-1.5	0.0209	1700025G04Rik	RIKEN cDNA 1700025G04 gene
17334819	1.31	0.0209	0610011F06Rik	RIKEN cDNA 0610011F06 gene
17300959	1.56	0.021	Rpl13-ps3	ribosomal protein L13, pseudogene 3 [Source:MGI Symbol;Acc:MGI:3646682]
17244189	2.08	0.021	Gm24119	predicted gene, 24119 [Source:MGI Symbol;Acc:MGI:5453896]
17465906	-1.63	0.021	Creb3l2	cAMP responsive element binding protein 3-like 2
17514255	1.83	0.021	Gm39287	predicted gene, 39287
17214487	1.56	0.021	Speg	SPEG complex locus
17517222	1.71	0.0211	Arhgap20	Rho GTPase activating protein 20
17425408	2.28	0.0213	Gm12519	predicted gene 12519
17267418	-1.74	0.0213		
17322327	-1.43	0.0214	Cbx5	chromobox 5
17224813	-1.51	0.0214	Dock10	dedicator of cytokinesis 10
17284349	-1.45	0.0215	Ighm	immunoglobulin heavy constant mu
17317233	-1.6	0.0215	Atad2	ATPase family, AAA domain containing 2
17550572	2.22	0.0215		
17492549	1.78	0.0217	Man2a2	mannosidase 2, alpha 2
17484907	1.52	0.0217	Snora52; Rplp2	small nucleolar RNA, H/ACA box 52; ribosomal protein, large P2
17522916	-1.62	0.022	Itga9	integrin alpha 9
17407926	1.66	0.022	Mrps21	mitochondrial ribosomal protein S21
17316700	-1.61	0.022	Azin1	antizyme inhibitor 1
17532629	2.33	0.022	mt-Ts2; mt-Tl2	mitochondrially encoded tRNA serine 2 [Source:MGI Symbol;Acc:MGI:102474]; mitochondrially encoded tRNA leucine 2 [Source:MGI Symbol;Acc:MGI:102481]
17273059	-1.71	0.022	LOC105246895; Gm11772	uncharacterized LOC105246895; predicted gene 11772 [Source:MGI Symbol;Acc:MGI:3649468]
17484080	-1.38	0.022	Fam175b	family with sequence similarity 175, member B
17317153	-1.47	0.022	Sntb1	syntrophin, basic 1
17351559	-1.62	0.022	Ldlrad4	low density lipoprotein receptor class A domain containing 4
17263463	1.3	0.022	Flcn	folliculin
17308965	-1.52	0.022	Diaph3	diaphanous related formin 3
17349922	-1.65	0.022	Pcdhb16	protocadherin beta 16
17524760	1.41	0.022	Kank2	KN motif and ankyrin repeat domains 2
17470105	-1.31	0.022	Csgalnact2	chondroitin sulfate N-acetylgalactosaminyltransferase 2
17333946	-1.8	0.022	Zfp948	zinc finger protein 948
17504160	1.27	0.022	Polr2c	polymerase (RNA) II (DNA directed) polypeptide C
17356288	1.41	0.022	Ctsf	cathepsin F
17465942	-3.35	0.0221	Atp6v0a4	ATPase, H+ transporting, lysosomal V0 subunit A4
17506137	1.49	0.0221	Wfdc1	WAP four-disulfide core domain 1
17315570	-1.59	0.0221	Nckap1l	NCK associated protein 1 like
17474983	2.16	0.0222	Cadm4	cell adhesion molecule 4
17282970	-3.11	0.0223	Dio2	deiodinase, iodothyronine, type II
17439842	-1.59	0.0226	Pkd2	polycystic kidney disease 2
17357717	2.08	0.0226	Gm25443	predicted gene, 25443 [Source:MGI Symbol;Acc:MGI:5455220]
17289181	-1.5	0.0226	Arsb	arylsulfatase B
17357004	1.79	0.0226	Macrod1	MACRO domain containing 1
17240190	-1.54	0.0227	Dse	dermatan sulfate epimerase
17420897	1.45	0.0227	Sdhb	succinate dehydrogenase complex, subunit B, iron sulfur (Ip)
17235268	1.38	0.0227	Ndufs7	NADH dehydrogenase (ubiquinone) Fe-S protein 7
17548836	-1.35	0.0227	Gm2036	predicted gene 2036
17251370	-1.49	0.0227	Myh10	myosin, heavy polypeptide 10, non-muscle
17466624	-1.49	0.0227	Tmem176b	transmembrane protein 176B
17338821	1.74	0.0228	Rpl36	ribosomal protein L36
17291767	-1.8	0.0228	Tubb2a	tubulin, beta 2A class IIA
17509891	1.43	0.0228	Ndufa13; Yjefn3	NADH dehydrogenase (ubiquinone) 1 alpha subcomplex, 13; YjeF N-terminal domain containing 3
17234910	1.44	0.0228	Cdc34	cell division cycle 34
17524944	-1.38	0.0229	Zfp599	zinc finger protein 599
17344140	-1.32	0.023	D17H6S56E-5	DNA segment, Chr 17, human D6S56E 5
17490432	1.61	0.023	Med25	mediator complex subunit 25
17516412	-1.74	0.023	3110039I08Rik	RIKEN cDNA 3110039I08 gene
17253700	1.28	0.023	Lyrm9	LYR motif containing 9
17224125	1.31	0.023	Mreg	melanoregulin
17213213	-1.61	0.023	Casp8	caspase 8
17297801	1.68	0.023	Gm2260; Gm2274	predicted gene 2260; predicted gene 2274
17297833	1.68	0.023	Gm2260; Gm2274	predicted gene 2260; predicted gene 2274
17368387	1.52	0.023	Gm23969	predicted gene, 23969 [Source:MGI Symbol;Acc:MGI:5453746]
17362197	1.37	0.023	Vegfb	vascular endothelial growth factor B
17342483	1.69	0.023	Fam195a	family with sequence similarity 195, member A
17547507	1.43	0.0232		
17344978	-1.38	0.0232	9130008F23Rik	RIKEN cDNA 9130008F23 gene
17438419	1.4	0.0232	2310040G07Rik	RIKEN cDNA 2310040G07 gene [Source:MGI Symbol;Acc:MGI:1917534]
17549214	1.94	0.0232		
17325438	-1.49	0.0232	Hcls1	hematopoietic cell specific Lyn substrate 1
17274346	-1.76	0.0232	5730507C01Rik	RIKEN cDNA 5730507C01 gene
17233536	-1.85	0.0232	P4ha1	procollagen-proline, 2-oxoglutarate 4-dioxygenase (proline 4-hydroxylase), alpha 1 polypeptide
17380741	1.66	0.0232	Rps21	ribosomal protein S21
17347307	1.4	0.0233	Heatr5b	HEAT repeat containing 5B
17374028	1.48	0.0234	Mpped2	metallophosphoesterase domain containing 2
17478730	-1.84	0.0234	Gabrb3	gamma-aminobutyric acid (GABA) A receptor, subunit beta 3
17462224	-1.99	0.0234	Gm25905	predicted gene, 25905 [Source:MGI Symbol;Acc:MGI:5455682]
17402357	-2.12	0.0234	1810037I17Rik	RIKEN cDNA 1810037I17 gene
17459014	-1.68	0.0234	Abcg2	ATP-binding cassette, sub-family G (WHITE), member 2
17505995	-1.68	0.0234	Plcg2	phospholipase C, gamma 2
17367055	-1.45	0.0234	Vim	vimentin
17412237	-1.38	0.0234	Mms22l	MMS22-like, DNA repair protein
17316956	-1.43	0.0234	Trps1	trichorhinophalangeal syndrome I (human)
17346749	-1.66	0.0234	Rab31	RAB31, member RAS oncogene family
17421545	-3.16	0.0234	Nppa	natriuretic peptide type A
17496645	1.37	0.0234	Dctpp1	dCTP pyrophosphatase 1
17522167	1.86	0.0235	Plxnb1	plexin B1
17427211	1.38	0.0235	Gm23443	predicted gene, 23443 [Source:MGI Symbol;Acc:MGI:5453220]
17307837	1.47	0.0236	Ephx2	epoxide hydrolase 2, cytoplasmic
17447259	-1.48	0.0236	Tnip2	TNFAIP3 interacting protein 2
17355189	-1.85	0.0236	Spire1	spire homolog 1 (Drosophila)
17343813	-1.51	0.0236	H2-Aa	histocompatibility 2, class II antigen A, alpha
17467040	1.84	0.0236	Crhr2	corticotropin releasing hormone receptor 2
17325109	-1.43	0.0237	Itgb5	integrin beta 5
17470820	1.43	0.0237	Grcc10	gene rich cluster, C10 gene
17359344	-1.48	0.0237	Entpd1	ectonucleoside triphosphate diphosphohydrolase 1
17405789	-1.57	0.0238	Trim59	tripartite motif-containing 59
17230153	1.31	0.0238	Fh1	fumarate hydratase 1
17252200	1.59	0.0238	Kif1c	kinesin family member 1C
17532635	2.37	0.0238	mt-Tt	mitochondrially encoded tRNA threonine [Source:MGI Symbol;Acc:MGI:102473]
17356811	1.35	0.024	Map4k2	mitogen-activated protein kinase kinase kinase kinase 2
17546234	-1.67	0.024	Vamp7	vesicle-associated membrane protein 7
17474400	1.39	0.024	Sympk	symplekin
17227753	-1.38	0.0241	Trove2	TROVE domain family, member 2
17482508	1.38	0.0242	Vwa3a	von Willebrand factor A domain containing 3A
17398406	-2.08	0.0242	Fam198b	family with sequence similarity 198, member B
17348467	1.53	0.0242	3110002H16Rik	RIKEN cDNA 3110002H16 gene
17433204	1.32	0.0242	Tmem201	transmembrane protein 201
17279317	1.99	0.0242	A530016L24Rik	RIKEN cDNA A530016L24 gene

17303472	-1.39	0.0243	Gm9800	predicted gene 9800 [Source:MGI Symbol;Acc:MGI:3710580]
17533994	-1.6	0.0243	Dock11	dedicator of cytokinesis 11
17286707	-1.51	0.0243	Gcnt2	glucosaminyl (N-acetyl) transferase 2, I-branching enzyme
17461166	-1.76	0.0245	Gxylt2	glucoside xylosyltransferase 2
17278761	-1.53	0.0245	AF357425	snoRNA AF357425
17271399	-1.62	0.0245	Abca9	ATP-binding cassette, sub-family A (ABC1), member 9
17230195	-1.33	0.0245	Cep170	centrosomal protein 170
17325514	-2.02	0.0247	Fstl1	follistatin-like 1
17307493	1.43	0.0248	Sprvd7	SPRY domain containing 7
17500996	1.29	0.0248	Acsl1	acyl-CoA synthetase long-chain family member 1
17301823	1.68	0.0248	Gfra2	glial cell line derived neurotrophic factor family receptor alpha 2
17400486	-1.6	0.0249	Mtmr11	myotubularin related protein 11
17308194	1.37	0.0249	Rhobtb2	Rho-related BTB domain containing 2
17312396	1.41	0.0249	Cyc1	cytochrome c-1
17538060	-1.73	0.0249	Mum1l1	melanoma associated antigen (mutated) 1-like 1
17422039	1.5	0.0249	Klhl21	kelch-like 21
17489824	-1.37	0.0249	Dpy19l3	dpy-19-like 3 (C. elegans)
17550502	-1.49	0.0249		
17380199	1.52	0.0249	Rbm38	RNA binding motif protein 38
17483383	1.77	0.0249	Snora30	small nucleolar RNA, H/ACA box 30
17547980	-1.46	0.0249	Olfr175-ps1	olfactory receptor 175, pseudogene 1
17369679	1.49	0.0249	Prrc2b	proline-rich coiled-coil 2B
17274120	2.2	0.0249	Rpl36-ps3	ribosomal protein L36, pseudogene 3 [Source:MGI Symbol;Acc:MGI:3642480]
17426890	-1.35	0.0249	Psip1	PC4 and SFRS1 interacting protein 1
17405764	-1.48	0.025	Ift80	intraflagellar transport 80
17474547	1.96	0.025	Rtn2	reticulon 2 (Z-band associated protein)
17393076	-1.4	0.025	Commd7	COMM domain containing 7
17400720	-1.5	0.025	Rnf115	ring finger protein 115
17475777	-1.6	0.0251	Fbl	fibrillarin
17371716	-1.36	0.0251	Dync1i2	dynein cytoplasmic 1 intermediate chain 2
17340084	-1.34	0.0251	Dync2li1	dynein cytoplasmic 2 light intermediate chain 1
17272770	-1.32	0.0251	Timp2	tissue inhibitor of metalloproteinase 2
17276139	-1.34	0.0251	Dact1	dapper homolog 1, antagonist of beta-catenin (xenopus)
17321263	1.56	0.0251	Adcy6	adenylate cyclase 6
17248104	-1.92	0.0251	Rtn4	reticulon 4
17449052	1.42	0.0251	Hopx	HOP homeobox
17435311	1.54	0.0251	Abcb8	ATP-binding cassette, sub-family B (MDR/TAP), member 8
17316725	-1.48	0.0251	Slc25a32	solute carrier family 25, member 32
17382864	1.53	0.0252	Qsox2	quiescin Q6 sulfhydryl oxidase 2
17245399	-1.43	0.0252	Irak3	interleukin-1 receptor-associated kinase 3
17258015	1.46	0.0252	Rpl38	ribosomal protein L38
17506073	1.75	0.0252	Mlycd	malonyl-CoA decarboxylase
17276328	-1.46	0.0252	Hif1a	hypoxia inducible factor 1, alpha subunit
17268909	-1.58	0.0255	Top2a	topoisomerase (DNA) II alpha
17427891	1.48	0.0255	Mrpl37	mitochondrial ribosomal protein L37
17546471	1.42	0.0255	Gm20810	predicted gene, 20810 [Source:MGI Symbol;Acc:MGI:5434166]
17497704	1.7	0.0255	Cox8b	cytochrome c oxidase subunit VIIIb
17532627	2.13	0.0255	mt-Th	mitochondrially encoded tRNA histidine [Source:MGI Symbol;Acc:MGI:102485]
17250585	-2.28	0.0256	Mfap4	microfibrillar-associated protein 4
17331669	-1.36	0.0256	App	amyloid beta (A4) precursor protein
17414836	-1.55	0.0256	Tlr4	toll-like receptor 4
17524107	-1.77	0.0256	Panx1	pannexin 1
17339013	-1.63	0.0256	Adgre1	adhesion G protein-coupled receptor E1
17456084	-1.68	0.0256	Foxp2	forkhead box P2
17290398	-1.44	0.0256	Gtpbp4	GTP binding protein 4
17244057	-2.07	0.0256	Gas2l3	growth arrest-specific 2 like 3
17258272	1.38	0.0258	Mrps7	mitchondrial ribosomal protein S7
17550568	2.13	0.0258		
17550570	2.13	0.0258		
17445507	-1.37	0.0258	Dbf4	DBF4 homolog (S. cerevisiae)
17432264	1.47	0.0258	Plekhm2	pleckstrin homology domain containing, family M (with RUN domain) member 2
17315743	-1.61	0.0259	Osmr	oncostatin M receptor
17386569	-1.33	0.0259	Chn1	chimerin 1
17483912	1.41	0.0259	Htra1	HtrA serine peptidase 1
17483420	1.9	0.0259	Ctf1	cardiotrophin 1
17409649	-1.5	0.0259	Vcam1	vascular cell adhesion molecule 1
17445710	-1.63	0.0259	Gm8991	predicted pseudogene 8991 [Source:MGI Symbol;Acc:MGI:3644227]
17331774	-1.37	0.0261	Cct8	chaperonin containing Tcp1, subunit 8 (theta)
17347448	-1.85	0.0262	Cyp1b1	cytochrome P450, family 1, subfamily b, polypeptide 1
17521971	1.45	0.0263	Slc26a6	solute carrier family 26, member 6
17223916	-1.69	0.0263	Myl1	myosin, light polypeptide 1
17305856	-1.47	0.0263	Exoc5	exocyst complex component 5
17212286	1.75	0.0263	Slc9a2	solute carrier family 9 (sodium/hydrogen exchanger), member 2
17290563	1.81	0.0265	Gm10336; Mtr	predicted gene 10336; 5-methyltetrahydrofolate-homocysteine methyltransferase
17437148	-1.53	0.0265	C1qtnf7	C1q and tumor necrosis factor related protein 7
17401539	1.32	0.0265	Kcna2	potassium voltage-gated channel, shaker-related subfamily, member 2
17309118	1.62	0.0265	Tbc1d4	TBC1 domain family, member 4
17237208	-1.62	0.0265	Csrp2	cysteine and glycine-rich protein 2
17215178	1.38	0.0265	Prss56	protease, serine 56
17258642	2.16	0.0265	Snord1b	small nucleolar RNA, C/D box 1B
17302606	-1.53	0.0265	Tpm3	tropomyosin 3, gamma
17263362	1.36	0.0266	Guk1	guanylate kinase 1
17362207	1.33	0.0266	Dnajc4	DnaJ (Hsp40) homolog, subfamily C, member 4
17515062	1.45	0.0266	Mrpl4	mitochondrial ribosomal protein L4
17241228	1.36	0.0267	Eif4ebp2	eukaryotic translation initiation factor 4E binding protein 2
17298090	-1.36	0.0269	Erc2	ELKS/RAB6-interacting/CAST family member 2
17536931	-1.46	0.0269	Gm24509; Gm25358	predicted gene, 24509 [Source:MGI Symbol;Acc:MGI:5454286]; predicted gene, 25358 [Source:MGI Symbol;Acc:MGI:5455135]
17536943	-1.46	0.0269	Gm24509; Gm25358	predicted gene, 24509 [Source:MGI Symbol;Acc:MGI:5454286]; predicted gene, 25358 [Source:MGI Symbol;Acc:MGI:5455135]
17503287	1.31	0.0269	Prdx2	peroxiredoxin 2
17242924	1.64	0.0269	Uqcr11	ubiquinol-cytochrome c reductase, complex III subunit XI
17474035	1.46	0.0269	Napa	N-ethylmaleimide sensitive fusion protein attachment protein alpha
17374053	1.36	0.027	Hadhb	hydroxyacyl-Coenzyme A dehydrogenase/3-ketoacyl-Coenzyme A thiolase/enoyl-Coenzyme A hydratase (trifunctional protein), beta subunit
17431938	1.35	0.027	Igsf21	immunoglobulin superfamily, member 21
17424279	1.31	0.027	Cntfr	ciliary neurotrophic factor receptor
17469320	-1.38	0.027	Tmf1	TATA element modulatory factor 1
17295212	-1.41	0.0271	Polk	polymerase (DNA directed), kappa
17543572	-1.52	0.0271	Il2rg	interleukin 2 receptor, gamma chain
17415076	-1.4	0.0272	Cntln	centlein, centrosomal protein
17312939	1.56	0.0273	Polr2f	polymerase (RNA) II (DNA directed) polypeptide F
17426206	1.4	0.0274	Alad	aminolevulinate, delta-, dehydratase
17217101	1.7	0.0274	Slc41a1	solute carrier family 41, member 1
17428477	1.89	0.0274	Cyp4b1	cytochrome P450, family 4, subfamily b, polypeptide 1
17326859	-1.31	0.0274	Usp16	ubiquitin specific peptidase 16
17238594	-1.41	0.0274	Cd63	CD63 antigen
17416616	1.53	0.0275	Echdc2	enoyl Coenzyme A hydratase domain containing 2
17265384	1.52	0.0275	Gm12318	predicted gene 12318
17260916	-1.46	0.0275	Ugp2	UDP-glucose pyrophosphorylase 2
17269695	1.31	0.0275	Stat5b	signal transducer and activator of transcription 5B
17457540	1.59	0.0276	Ndufb2	NADH dehydrogenase (ubiquinone) 1 beta subcomplex, 2
17274875	1.43	0.0276	Nampt	nicotinamide phosphoribosyltransferase
17276934	-1.73	0.0276	Ttc9	tetratricopeptide repeat domain 9
17310872	-1.49	0.0276	Sdc2	syndecan 2
17315482	1.95	0.0276	Gm4544	predicted gene 4544
17394297	1.37	0.0276	Pltp	phospholipid transfer protein
17314011	1.96	0.0276	Pim3; Gm33049	proviral integration site 3; predicted gene, 33049
17514135	1.31	0.0277	Egln1	egl-9 family hypoxia-inducible factor 1
17425701	-2.16	0.0277	Mir3095	microRNA 3095
17378596	1.57	0.0277	Gm22759; Gm14251	predicted gene, 22759 [Source:MGI Symbol;Acc:MGI:5452536]; predicted gene 14251 [Source:MGI Symbol;Acc:MGI:3650992]
17352862	1.78	0.0278	Aqp4	aquaporin 4
17282158	-1.37	0.0278	Rdh11	retinol dehydrogenase 11
17465856	-1.82	0.0278	Ptn	pleiotrophin
17436931	1.64	0.0278	Grpel1	GrpE-like 1, mitochondrial
17477679	1.34	0.0278	Nosip	nitric oxide synthase interacting protein
17362111	1.39	0.0278	Esrra; Mir6990	estrogen related receptor, alpha; microRNA 6990
17539027	-1.42	0.0278	2210013O21Rik	RIKEN cDNA 2210013O21 gene
17342029	1.38	0.0278	Ndufb10	NADH dehydrogenase (ubiquinone) 1 beta subcomplex, 10
17358832	-1.42	0.0278	Ifit1	interferon-induced protein with tetratricopeptide repeats 1
17211004	1.66	0.0279	Adhfe1	alcohol dehydrogenase, iron containing, 1
17432440	-1.51	0.0279	Pdpn	podoplanin
17273073	1.44	0.028	Faap100	Fanconi anemia core complex associated protein 100
17350040	1.62	0.0283	Arhgap26	Rho GTPase activating protein 26
17217048	-1.52	0.0283	Rab7b	RAB7B, member RAS oncogene family
17478745	-1.44	0.0283	Atp10a	ATPase, class V, type 10A
17549186	-1.38	0.0283		
17537581	-1.45	0.0284	Tnmd	tenomodulin
17361764	1.56	0.0284	Fam89b; Sssca1	family with sequence similarity 89, member B; Sjogrens syndrome/scleroderma autoantigen 1 homolog (human)
17514853	-1.5	0.0284	Slc36a4	solute carrier family 36 (proton/amino acid symporter), member 4
17411135	1.96	0.0284	Gm10287	predicted gene 10287 [Source:MGI Symbol;Acc:MGI:3642138]
17529222	1.29	0.0284	Gm26907	predicted gene, 26907
17265268	-1.56	0.0284	Cxcl16	chemokine (C-X-C motif) ligand 16
17481594	1.64	0.0284	Olfr473	olfactory receptor 473
17263223	1.33	0.0284	Obscn	obscurin, cytoskeletal calmodulin and titin-interacting RhoGEF
17390844	-1.51	0.0284	Myef2	myelin basic protein expression factor 2, repressor
17371460	-1.31	0.0285	Ppig	peptidyl-prolyl isomerase G (cyclophilin G)
17230256	-1.54	0.0285	Adss	adenylosuccinate synthetase, non muscle
17527353	1.32	0.0285	Etfa	electron transferring flavoprotein, alpha polypeptide
17239787	-2.2	0.0285	Enpp1	ectonucleotide pyrophosphatase/phosphodiesterase 1
17545159	-1.77	0.0285	Amot	angiomotin
17327502	-1.45	0.0285	Sh3bgr	SH3-binding domain glutamic acid-rich protein
17360462	-1.45	0.0285	Acsl5	acyl-CoA synthetase long-chain family member 5
17437803	1.38	0.0286	N4bp2	NEDD4 binding protein 2
17310893	-1.42	0.0286	Mtdh	metadherin
17300591	-1.33	0.0286	Irf9	interferon regulatory factor 9
17338124	1.33	0.0286	Mrpl2	mitochondrial ribosomal protein L2
17419287	-1.47	0.0287	Laptm5	lysosomal-associated protein transmembrane 5
17247652	1.6	0.0288	Zrsr1	zinc finger (CCCH type), RNA binding motif and serine/arginine rich 1
17224771	-1.73	0.0289	Serpine2	serine (or cysteine) peptidase inhibitor, clade E, member 2
17310432	-1.48	0.029	Mtmr12	myotubularin related protein 12
17468730	-1.34	0.0291	Sec61a1	Sec61 alpha 1 subunit (S. cerevisiae)
17546101	-1.42	0.0292	Tlr8	toll-like receptor 8
17532540	1.45	0.0295	Fyco1; Gm17021	FYVE and coiled-coil domain containing 1; predicted gene 17021
17259507	1.57	0.0296	Hmga1-rs1	high mobility group AT-hook I, related sequence 1
17235539	1.38	0.0296	Map2k2	mitogen-activated protein kinase kinase 2
17288859	-1.49	0.0296	A830082K12Rik; Gm8327	RIKEN cDNA A830082K12 gene; predicted gene 8327
17431820	2.54	0.0296	Pla2g5	phospholipase A2, group V
17448905	-1.67	0.0296	Chic2	cysteine-rich hydrophobic domain 2
17295503	1.37	0.0296	Mccc2	methylcrotonoyl-Coenzyme A carboxylase 2 (beta)
17548585	-1.49	0.0296	Gm4887	predicted gene 4887
17548211	1.54	0.0296	LOC102632821; Gm13777	60S acidic ribosomal protein P1-like; predicted gene 13777 [Source:MGI Symbol;Acc:MGI:3650376]
17277794	-1.53	0.0296	Gpr65	G-protein coupled receptor 65
17273116	1.6	0.0296	Oxld1	oxidoreductase like domain containing 1
17497904	2.06	0.0298	Slc25a22	solute carrier family 25 (mitochondrial carrier, glutamate), member 22
17427104	1.71	0.0298	Ifnab	interferon alpha B
17243312	1.29	0.0299	Fzr1	fizzy/cell division cycle 20 related 1 (Drosophila)
17313862	1.72	0.0299	Ppara	peroxisome proliferator activated receptor alpha
17503937	-1.97	0.03	Mt2	metallothionein 2
17272509	-1.56	0.0304	Mxra7	matrix-remodelling associated 7
17368009	1.52	0.0304	Abca2	ATP-binding cassette, sub-family A (ABC1), member 2
17295482	-1.42	0.0304	Map1b	microtubule-associated protein 1B
17375220	-1.43	0.0307	Tubgcp4	tubulin, gamma complex associated protein 4
17257579	1.51	0.0307	Prr29	proline rich 29
17223946	1.43	0.0308	Erbb4	v-erb-a erythroblastic leukemia viral oncogene homolog 4 (avian)
17541713	1.68	0.0309	Gm14586	predicted gene 14586
17427970	-1.39	0.0309	Podn	podocan
17496193	-1.36	0.031	Cd19	CD19 antigen
17353131	-1.39	0.031	Ino80c	INO80 complex subunit C
17229391	-2.42	0.031	Uck2	uridine-cytidine kinase 2
17492478	1.27	0.031	Idh2	isocitrate dehydrogenase 2 (NADP+), mitochondrial
17221662	-1.58	0.031	Tram2	translocating chain-associating membrane protein 2
17393541	1.56	0.031	Gm22758; Gm14173	predicted gene, 22758 [Source:MGI Symbol;Acc:MGI:5452535]; predicted gene 14173 [Source:MGI Symbol;Acc:MGI:3650930]
17548023	-1.52	0.031	LOC102633627; Gm7809	tropomyosin alpha-4 chain pseudogene; predicted gene 7809 [Source:MGI Symbol;Acc:MGI:3643362]
17502944	1.68	0.031	Ndufb7	NADH dehydrogenase (ubiquinone) 1 beta subcomplex, 7
17295270	-1.32	0.031	Nsa2	NSA2 ribosome biogenesis homolog (S. cerevisiae)
17337794	1.38	0.031	Gm25135	predicted gene, 25135 [Source:MGI Symbol;Acc:MGI:5454912]
17392472	1.41	0.031	Ralgapa2	Ral GTPase activating protein, alpha subunit 2 (catalytic)
17382673	1.39	0.031	Phpt1; Mamdc4	phosphohistidine phosphatase 1; MAM domain containing 4
17260440	1.4	0.031	Snora5c; Tbrg4	small nucleolar RNA, H/ACA box 5C; transforming growth factor beta regulated gene 4
17236476	-1.61	0.0311	Ikbip	IKBKB interacting protein
17226593	-1.67	0.0311	Cxcr4	chemokine (C-X-C motif) receptor 4
17510856	1.8	0.0312	Il15	interleukin 15
17469463	-1.42	0.0312	Rybp	RING1 and YY1 binding protein
17344456	1.33	0.0314	2310061I04Rik	RIKEN cDNA 2310061I04 gene
17469587	1.57	0.0316	Gm4604	predicted gene 4604

17328792	1.38	0.0316	Dgcr2	DiGeorge syndrome critical region gene 2
17388177	1.4	0.0316	Nr1h3	nuclear receptor subfamily 1, group H, member 3
17361056	1.47	0.0316	Ndufv1	NADH dehydrogenase (ubiquinone) flavoprotein 1
17338925	1.53	0.0316	Clpp	caseinolytic mitochondrial matrix peptidase proteolytic subunit
17449330	-1.69	0.0316	Gm24121	predicted gene, 24121 [Source:MGI Symbol;Acc:MGI:5453898]
17242773	1.53	0.0316	Polr2e	polymerase (RNA) II (DNA directed) polypeptide E
17302832	-1.72	0.0318	B930095G15Rik	RIKEN cDNA B930095G15 gene
17334846	1.47	0.0318	Tmem8	transmembrane protein 8 (five membrane-spanning domains)
17485226	-1.36	0.0318	Lsp1	lymphocyte specific 1
17442128	-1.37	0.0319	P2rx7	purinergic receptor P2X, ligand-gated ion channel, 7
17222719	-1.68	0.0319	Col5a2	collagen, type V, alpha 2
17535245	-2.04	0.0319	Aff2	AF4/FMR2 family, member 2
17406279	-1.58	0.032	Tlr2	toll-like receptor 2
17431234	-1.58	0.032	Cnksr1	connector enhancer of kinase suppressor of Ras 1
17459338	-2.05	0.0321	Igk-V1; Igkv1-110; Igk-V5	immunoglobulin kappa chain variable 1 (V1); immunoglobulin kappa variable 1-110; immunoglobulin kappa chain variable 5 (V5 family)
17477468	2.15	0.0321	Lrrc4b	leucine rich repeat containing 4B
17395379	-1.43	0.0321	Gm14412	predicted gene 14412 [Source:MGI Symbol;Acc:MGI:3652251]
17549588	-2.19	0.0322		
17549860	-2.19	0.0322		
17258958	1.53	0.0322	Gm26888	predicted gene, 26888 [Source:MGI Symbol;Acc:MGI:5477382]
17456339	-1.42	0.0323	Cped1	cadherin-like and PC-esterase domain containing 1
17293059	1.36	0.0323	Klhl3	kelch-like 3
17262277	-1.4	0.0326	Cnot6	CCR4-NOT transcription complex, subunit 6
17406002	-1.49	0.0328	Golim4	golgi integral membrane protein 4
17372515	-1.28	0.0328	Itgav	integrin alpha V
17305685	-1.42	0.0328	Ddhd1	DDHD domain containing 1
17295266	-1.32	0.033	Gm6169	predicted gene 6169 [Source:MGI Symbol;Acc:MGI:3646298]
17222825	-1.78	0.0331	Nabp1	nucleic acid binding protein 1
17357981	-1.52	0.0331	Prune2	prune homolog 2 (Drosophila)
17525391	1.84	0.0331	Gm5614	predicted gene 5614 [Source:MGI Symbol;Acc:MGI:3644422]
17490417	1.42	0.0332	Ptov1	prostate tumor over expressed gene 1
17401833	-1.47	0.0332	Slc25a24	solute carrier family 25 (mitochondrial carrier, phosphate carrier), member 24
17233930	1.39	0.0334	Ctnna3	catenin (cadherin associated protein), alpha 3
17213336	-1.4	0.0335	Bmpr2	bone morphogenetic protein receptor, type II (serine/threonine kinase)
17435055	-2.06	0.0335	Gsap	gamma-secretase activating protein
17404821	-2.21	0.0337	Ccna2	cyclin A2
17308347	-1.4	0.0337	Gm24890	predicted gene, 24890 [Source:MGI Symbol;Acc:MGI:5454667]
17541926	1.38	0.0337	Fgf13	fibroblast growth factor 13
17317056	1.76	0.0337	Enpp2	ectonucleotide pyrophosphatase/phosphodiesterase 2
17548852	-1.31	0.0337	LOC105243690; Gm14681	nucleophosmin-like; predicted gene 14681 [Source:MGI Symbol;Acc:MGI:3705734]
17510422	-1.37	0.0337	B3gnt3	UDP-GlcNAc:betaGal beta-1,3-N-acetylglucosaminyltransferase 3
17390186	-1.44	0.0337	Tmem87a	transmembrane protein 87A
17545488	-1.87	0.0339	Kctd12b	potassium channel tetramerisation domain containing 12b
17471155	1.38	0.034	Kcna1	potassium voltage-gated channel, shaker-related subfamily, member 1
17547827	1.38	0.034	Gm35595; Gm5802	predicted gene, 35595; predicted gene 5802
17451195	1.5	0.034	Cryba4	crystallin, beta A4
17518664	-1.33	0.0342	Ppib	peptidylprolyl isomerase B
17374455	-1.56	0.0343	Spred1	sprouty protein with EVH-1 domain 1, related sequence
17540027	-1.32	0.0344	Rbm3	RNA binding motif protein 3
17377268	1.28	0.0344	Kiz	kizuna centrosomal protein
17316270	-1.76	0.0346	Gm23033	predicted gene, 23033 [Source:MGI Symbol;Acc:MGI:5452810]
17439481	-1.36	0.0346	Bmp2k	BMP2 inducible kinase
17240342	-1.51	0.0346	Rpf2	ribosome production factor 2 homolog (S. cerevisiae)
17547767	-1.59	0.0346		
17445869	-1.34	0.0346	Dnajc2	DnaJ (Hsp40) homolog, subfamily C, member 2
17231717	-1.57	0.0346	Fuca2	fucosidase, alpha-L- 2, plasma
17270703	-1.52	0.0346	Cyb561	cytochrome b-561
17481684	-1.47	0.0346	Akip1	A kinase (PRKA) interacting protein 1
17251643	1.34	0.0348	Naa38	N(alpha)-acetyltransferase 38, NatC auxiliary subunit
17453140	2.58	0.0349	Phkg1	phosphorylase kinase gamma 1
17332932	1.32	0.035	Sytl3	synaptotagmin-like 3
17250829	1.3	0.035	Tekt3	tektin 3
17289695	-1.44	0.035	Smim15	small integral membrane protein 15
17396492	-1.65	0.035	Eif5a2	eukaryotic translation initiation factor 5A2
17479736	-1.56	0.035	Tm6sf1	transmembrane 6 superfamily member 1
17290457	1.41	0.0352	Ryr2	ryanodine receptor 2, cardiac
17407969	-1.57	0.0352	Plekho1	pleckstrin homology domain containing, family O member 1
17278410	-1.46	0.0353	Gskip	GSK3B interacting protein
17293863	-2.12	0.0353	Uqcrb	ubiquinol-cytochrome c reductase binding protein
17468444	1.35	0.0353	Cml2	camello-like 2
17288695	1.5	0.0353	Mir682; Rpl37a	microRNA 682; ribosomal protein L37a
17507171	1.69	0.0354	Rprl3	ribonuclease P RNA-like 3
17260221	1.49	0.0354	Pold2	polymerase (DNA directed), delta 2, regulatory subunit
17542695	-1.76	0.0354	G6pdx	glucose-6-phosphate dehydrogenase X-linked
17408024	-1.57	0.0356	Fcgr1	Fc receptor, IgG, high affinity I
17302656	-1.61	0.0356	Gpc6	glypican 6
17542853	-1.47	0.0356	Pls3	plastin 3 (T-isoform)
17238449	1.33	0.0356	Ankrd52	ankyrin repeat domain 52
17243361	1.52	0.0356	Ncln	nicalin homolog (zebrafish)
17357054	-1.34	0.0357	Atl3	atlastin GTPase 3
17316415	-1.35	0.0359	Stk3	serine/threonine kinase 3
17504005	1.65	0.0359	Herpud1	homocysteine-inducible, endoplasmic reticulum stress-inducible, ubiquitin-like domain member 1
17321351	1.29	0.0359	Prkag1	protein kinase, AMP-activated, gamma 1 non-catalytic subunit
17365106	1.58	0.0359	Sec31b	Sec31 homolog B (S. cerevisiae)
17351001	1.56	0.0361	Camk2a	calcium/calmodulin-dependent protein kinase II alpha
17360084	1.38	0.0361	As3mt	arsenic (+3 oxidation state) methyltransferase
17451972	1.4	0.0361	Plbd2	phospholipase B domain containing 2
17295877	-1.39	0.0361	Erbb2ip	Erbb2 interacting protein
17316189	-1.57	0.0362	Otulin	OTU deubiquitinase with linear linkage specificity
17361223	1.51	0.0362	Adrbk1	adrenergic receptor kinase, beta 1
17543196	-1.56	0.0362	Pdk3	pyruvate dehydrogenase kinase, isoenzyme 3
17439164	-1.42	0.0362	11. Sep	septin 11
17456414	1.46	0.0363	Asb15	ankyrin repeat and SOCS box-containing 15
17529692	-1.58	0.0363	B430319G15Rik	RIKEN cDNA B430319G15 gene
17358448	3.09	0.0363	Kcnv2	potassium channel, subfamily V, member 2
17482095	-1.97	0.0363	Nucb2	nucleobindin 2
17397632	-1.47	0.0363	Alg5	asparagine-linked glycosylation 5 (dolichyl-phosphate beta-glucosyltransferase)
17235198	1.32	0.0363	Atp5d	ATP synthase, H+ transporting, mitochondrial F1 complex, delta subunit
17397708	-1.45	0.0364	Dclk1	doublecortin-like kinase 1
17291904	-1.35	0.0365	Ssr1	signal sequence receptor, alpha
17223390	-1.41	0.0365	Orc2	origin recognition complex, subunit 2
17474621	1.47	0.0367	Ckm	creatine kinase, muscle
17449958	-1.75	0.0367	Mir703	microRNA 703
17302096	1.38	0.0367	1700108F19Rik	RIKEN cDNA 1700108F19 gene
17493995	-1.51	0.0367	Il18bp	interleukin 18 binding protein
17240226	-1.45	0.0367	Marcks	myristoylated alanine rich protein kinase C substrate
17216870	-1.41	0.0368	Ubxn4	UBX domain protein 4
17395766	1.27	0.0368	Eef1a2	eukaryotic translation elongation factor 1 alpha 2
17389578	-1.31	0.0368	Dph6	diphthamine biosynthesis 6
17326510	-1.37	0.0368	Pros1	protein S (alpha)
17536364	-1.42	0.0368	Msn	moesin
17451390	-1.53	0.0368	Cmklr1	chemokine-like receptor 1
17280310	-1.43	0.0368	Id2	inhibitor of DNA binding 2
17283897	1.48	0.0368	Ccdc85c	coiled-coil domain containing 85C
17289207	-1.31	0.0368	Ap3b1	adaptor-related protein complex 3, beta 1 subunit
17537592	-1.68	0.037	Srpx2	sushi-repeat-containing protein, X-linked 2
17514601	1.42	0.037	Gm29376	predicted gene 29376
17340673	-1.52	0.037	Fndc1	fibronectin type III domain containing 1
17515413	1.36	0.037	Pigyl	phosphatidylinositol glycan anchor biosynthesis, class Y-like
17532597	1.57	0.037	mt-Tv	mitochondrially encoded tRNA valine [Source:MGI Symbol;Acc:MGI:102472]
17304246	-1.36	0.037	Appl1	adaptor protein, phosphotyrosine interaction, PH domain and leucine zipper containing 1
17245729	1.8	0.0371	Slc26a10	solute carrier family 26, member 10
17280571	1.91	0.0372	Gpr22	G protein-coupled receptor 22
17413474	-1.65	0.0372	Reck	reversion-inducing-cysteine-rich protein with kazal motifs
17359304	1.73	0.0372	Cyp2c39	cytochrome P450, family 2, subfamily c, polypeptide 39
17399702	1.38	0.0372	Dennd4b	DENN/MADD domain containing 4B
17406604	1.35	0.0373	Prcc	papillary renal cell carcinoma (translocation-associated)
17465824	-1.95	0.0373	Fam180a	family with sequence similarity 180, member A
17541528	-1.55	0.0373	Igsf1	immunoglobulin superfamily, member 1
17424243	1.72	0.0373	AI464131	expressed sequence AI464131
17390879	-1.91	0.0373	Fbn1	fibrillin 1
17365803	-1.42	0.0373	Nrap	nebulin-related anchoring protein
17257937	1.39	0.0373	Kcnj2	potassium inwardly-rectifying channel, subfamily J, member 2
17221191	1.29	0.0373	Snord87	small nucleolar RNA, C/D box 87
17547567	1.29	0.0373	Gm11964	predicted gene 11964 [Source:MGI Symbol;Acc:MGI:3652241]
17489873	-1.39	0.0373	Uri1	URI1, prefoldin-like chaperone
17540323	-1.75	0.0373	n-R5s3	nuclear encoded rRNA 5S 3 [Source:MGI Symbol;Acc:MGI:4421737]
17282868	-1.49	0.0373	Sptlc2	serine palmitoyltransferase, long chain base subunit 2
17323627	1.28	0.0373	Vpreb2	pre-B lymphocyte gene 2
17447404	1.74	0.0373	Sorcs2	sortilin-related VPS10 domain containing receptor 2
17480048	1.36	0.0376	Me3	malic enzyme 3, NADP(+)-dependent, mitochondrial
17239719	-1.52	0.0377	Eya4	eyes absent 4 homolog (Drosophila)
17448614	1.43	0.0377	Corin	corin
17291361	1.62	0.0377	Aldh5a1	aldhehyde dehydrogenase family 5, subfamily A1
17305986	2.7	0.0377	Rpph1	ribonuclease P RNA component H1
17361988	1.4	0.0377	Arl2	ADP-ribosylation factor-like 2
17502039	1.52	0.0378	Rab3a	RAB3A, member RAS oncogene family
17530503	1.51	0.0379	Col6a6	collagen, type VI, alpha 6
17424088	1.97	0.0379	Mir5123	microRNA 5123
17343026	1.46	0.0379	Ppil1	peptidylprolyl isomerase (cyclophilin)-like 1
17293473	1.4	0.0379		
17397394	-2.51	0.0379	Gm26081	predicted gene, 26081 [Source:MGI Symbol;Acc:MGI:5455858]
17233708	1.34	0.0379	Lrrc20	leucine rich repeat containing 20
17429613	-1.29	0.0379	Cap1	CAP, adenylate cyclase-associated protein 1 (yeast)
17284358	-1.8	0.038	Ighj2	immunoglobulin heavy joining 2 [Source:MGI Symbol;Acc:MGI:4439803]
17304493	-1.55	0.0381	Gnl3	guanine nucleotide binding protein-like 3 (nucleolar)
17548525	-1.56	0.0381	Hnrnpf	heterogeneous nuclear ribonucleoprotein F
17467154	1.61	0.0381	Ppm1k	protein phosphatase 1K (PP2C domain containing)
17325274	-1.62	0.0381	Parp9	poly (ADP-ribose) polymerase family, member 9
17356482	1.32	0.0381	Rnaseh2c	ribonuclease H2, subunit C
17336190	1.91	0.0382	Ndufa7	NADH dehydrogenase (ubiquinone) 1 alpha subcomplex, 7 (B14.5a)
17522761	-1.53	0.0384	Dync1li1	dynein cytoplasmic 1 light intermediate chain 1
17239142	-1.55	0.0384	Samd5	sterile alpha motif domain containing 5
17294289	-2.03	0.0385	Nkd2	naked cuticle 2 homolog (Drosophila)
17247369	1.43	0.0385	Gm22789	predicted gene, 22789 [Source:MGI Symbol;Acc:MGI:5452566]
17327815	1.4	0.0385	Cdip1	cell death inducing Trp53 target 1
17398565	1.47	0.0385	Gm6525	ribosomal protein L36a pseudogene
17215932	1.44	0.0385	Sned1	sushi, nidogen and EGF-like domains 1
17516047	1.36	0.0385	Robo4	roundabout homolog 4 (Drosophila)
17550574	1.59	0.0385		
17405737	-1.4	0.0385	Lxn	latexin
17513641	-1.72	0.0385	Slc7a5	solute carrier family 7 (cationic amino acid transporter, y+ system), member 5
17508025	-1.54	0.0386	Ckap2	cytoskeleton associated protein 2
17296281	-1.33	0.0386	Gpx8	glutathione peroxidase 8 (putative)
17326541	-1.45	0.0386	Vgll3	vestigial like 3 (Drosophila)
17404796	-1.78	0.0386	Anxa5	annexin A5
17324553	-1.31	0.0386	Ccdc50	coiled-coil domain containing 50
17468961	1.6	0.0386	Nup210	nucleoporin 210
17237958	1.31	0.0386	Dctn2	dynactin 2
17357207	1.35	0.0387	Tmem223; Nxf1	transmembrane protein 223; nuclear RNA export factor 1
17469894	1.33	0.0387	Raf1	v-raf-leukemia viral oncogene 1
17529529	-1.29	0.0388	Syncrip	synaptotagmin binding, cytoplasmic RNA interacting protein
17376541	-1.52	0.039	Prnp; Prn	prion protein; prion protein gene complex
17492220	-1.57	0.039	Sv2b	synaptic vesicle glycoprotein 2 b
17353699	-1.85	0.039	Hbegf	heparin-binding EGF-like growth factor
17481695	-1.38	0.039	Ipo7	importin 7
17287185	-1.38	0.039	Nol8	nucleolar protein 8
17214622	1.48	0.039	Slc4a3	solute carrier family 4 (anion exchanger), member 3
17550580	2.12	0.039		
17365444	-1.36	0.0391	Pcgf6	polycomb group ring finger 6
17424608	-1.51	0.0391	Cd72	CD72 antigen
17241646	-1.37	0.0391	Zfp365	zinc finger protein 365
17360890	-1.48	0.0391	Grk5	G protein-coupled receptor kinase 5
17367367	1.34	0.0391	Msrb2	methionine sulfoxide reductase B2
17359340	-1.84	0.0391	Gm27042	predicted gene, 27042
17286621	-1.35	0.0391	Riok1	RIO kinase 1 (yeast)
17450618	1.46	0.0391	Tgfbr3	transforming growth factor, beta receptor III
17324274	-1.42	0.0391	Senp2	SUMO/sentrin specific peptidase 2
17475898	1.39	0.0391	Sars2	seryl-aminoacyl-tRNA synthetase 2
17489369	1.37	0.0391	Usf2	upstream transcription factor 2
17241934	1.54	0.0391	Gstt1	glutathione S-transferase, theta 1
17258186	1.31	0.0391	Ict1	immature colon carcinoma transcript 1
17230408	1.31	0.0391	Adck3	aarF domain containing kinase 3

17550450	-1.35	0.0391		
17215115	-1.33	0.0391	Ptma	prothymosin alpha
17425836	-1.26	0.0391	Ptbp3; Gm38518	polypyrimidine tract binding protein 3; predicted gene, 38518
17412552	-1.33	0.0391	Ube2j1	ubiquitin-conjugating enzyme E2J 1
17508907	-1.27	0.0396	Mtus1	mitochondrial tumor suppressor 1
17417090	1.62	0.0396	Trabd2b	TraB domain containing 2B
17272870	1.48	0.0396	Gm11755	predicted gene 11755
17419731	1.43	0.0396	Trim63	tripartite motif-containing 63
17459236	-1.41	0.0398	Mad2l1	MAD2 mitotic arrest deficient-like 1
17346975	1.72	0.0398	Emilin2	elastin microfibril interfacer 2
17347281	-1.27	0.0398	Strn	striatin, calmodulin binding protein
17396369	1.35	0.0398	Nceh1; Gm42192	neutral cholesterol ester hydrolase 1; predicted gene, 42192
17467690	1.72	0.0398	Ptcd3	pentatricopeptide repeat domain 3
17324446	-1.38	0.0398	Rtp4	receptor transporter protein 4
17311213	-1.78	0.0398	Rims2	regulating synaptic membrane exocytosis 2
17300544	1.44	0.0398	Fitm1	fat storage-inducing transmembrane protein 1
17231784	1.27	0.0398	Cited2	Cbp/p300-interacting transactivator, with Glu/Asp-rich carboxy-terminal domain, 2
17229842	-1.35	0.0399	Pea15a	phosphoprotein enriched in astrocytes 15A
17316043	-1.56	0.04	Npr3	natriuretic peptide receptor 3
17548928	-1.27	0.04	LOC102639127; Gm7785	zinc finger protein 330 pseudogene; predicted gene 7785 [Source:MGI Symbol;Acc:MGI:3645449]
17425248	1.26	0.04	Tmem246	transmembrane protein 246
17385840	1.33	0.04	Fign	fidgetin
17255719	1.23	0.04	Mrpl10	mitochondrial ribosomal protein L10
17224071	-1.96	0.0401	Fn1	fibronectin 1
17369120	1.39	0.0401	Endog	endonuclease G
17285586	-1.73	0.0401	Aoah	acyloxyacyl hydrolase
17324756	1.43	0.0403	Smco1	single-pass membrane protein with coiled-coil domains 1
17255987	2.2	0.0403	Tcap	titin-cap
17427494	-1.34	0.0403	Dock7	dedicator of cytokinesis 7
17277134	1.63	0.0403	Acot2	acyl-CoA thioesterase 2
17513275	1.25	0.0403	Gm16116	predicted gene 16116
17440826	1.6	0.0403	Acacb	acetyl-Coenzyme A carboxylase beta
17334970	1.26	0.0403	Bnip1	BCL2/adenovirus E1B interacting protein 1
17379304	1.28	0.0403	Pkig	protein kinase inhibitor, gamma
17466195	1.33	0.0404	Mrps33	mitochondrial ribosomal protein S33
17398082	-1.29	0.0404	Tiparp	TCDD-inducible poly(ADP-ribose) polymerase
17455801	-1.58	0.0404	Col1a2	collagen, type I, alpha 2
17340197	1.42	0.0405	Epas1	endothelial PAS domain protein 1
17332236	-1.66	0.0405	Runx1	runt related transcription factor 1
17474480	1.44	0.0406	Six5	sine oculis-related homeobox 5
17350011	-1.36	0.0407	Ndfip1	Nedd4 family interacting protein 1
17455159	1.31	0.0407	Cyp3a44	cytochrome P450, family 3, subfamily a, polypeptide 44
17427961	1.39	0.0407	Cpt2	carnitine palmitoyltransferase 2
17315993	-1.47	0.0408	Rai14	retinoic acid induced 14
17509127	1.46	0.0409	1700029J07Rik	RIKEN cDNA 1700029J07 gene
17364474	-1.55	0.0409	Pdlim1	PDZ and LIM domain 1 (elfin)
17228544	-1.68	0.0409	Soat1	sterol O-acyltransferase 1
17450741	1.48	0.0409	Atp5k	ATP synthase, H+ transporting, mitochondrial F1F0 complex, subunit E
17284049	1.31	0.0409	Ankrd9	ankyrin repeat domain 9
17381457	1.31	0.0409	Echdc3	enoyl Coenzyme A hydratase domain containing 3
17358777	-1.24	0.041	Stambpl1	STAM binding protein like 1
17423136	1.31	0.041	Gm11816	predicted gene 11816
17314691	1.68	0.041	Gm8973	predicted gene 8973 [Source:MGI Symbol;Acc:MGI:3648534]
17446686	1.69	0.041		
17487884	1.56	0.0411	Gsk3a	glycogen synthase kinase 3 alpha
17405075	-1.3	0.0412	Pcdh18	protocadherin 18
17470616	-1.51	0.0413	C3ar1	complement component 3a receptor 1
17370522	-1.75	0.0415	Gm13456	predicted gene 13456 [Source:MGI Symbol;Acc:MGI:3651389]
17219489	1.33	0.0415	Pex19	peroxisomal biogenesis factor 19
17536759	1.26	0.0415	Itgb1bp2	integrin beta 1 binding protein 2
17409668	-1.33	0.0415	Cdc14a	CDC14 cell division cycle 14A
17395466	1.22	0.0415	Taf4a	TAF4A RNA polymerase II, TATA box binding protein (TBP)-associated factor
17264946	-1.49	0.0415	Chrnb1	cholinergic receptor, nicotinic, beta polypeptide 1 (muscle)
17462191	-1.3	0.0415	Hnrnpf	heterogeneous nuclear ribonucleoprotein F
17331148	1.63	0.0415	Cmss1	cms small ribosomal subunit 1
17262702	-1.79	0.0415	Rad50	RAD50 homolog (S. cerevisiae)
17427883	1.59	0.0415	Gm12746	predicted gene 12746 [Source:MGI Symbol;Acc:MGI:3652135]
17512816	1.46	0.0416	Dhodh	dihydroorotate dehydrogenase
17311602	-1.4	0.0417	Mtbp	Mdm2, transformed 3T3 cell double minute p53 binding protein
17468678	-1.44	0.0417	Isy1	ISY1 splicing factor homolog (S. cerevisiae)
17490622	1.48	0.0418	Snord35a	small nucleolar RNA, C/D box 35A
17265096	1.38	0.0419	Slc2a4	solute carrier family 2 (facilitated glucose transporter), member 4
17290093	-2.1	0.0419	Hcn1	hyperpolarization-activated, cyclic nucleotide-gated K+ 1
17392209	-1.28	0.0419	Esf1	ESF1, nucleolar pre-rRNA processing protein, homolog (S. cerevisiae)
17355009	-1.43	0.0419	Nars	asparaginyl-tRNA synthetase
17231040	1.32	0.042	Nenf	neuron derived neurotrophic factor
17457184	-1.33	0.042	Cald1	caldesmon 1
17358132	-1.22	0.042	Abhd17b	abhydrolase domain containing 17B
17455494	1.78	0.042	Gm24105	predicted gene, 24105
17477527	1.36	0.042	2310016G11Rik	RIKEN cDNA 2310016G11 gene
17403571	-1.55	0.042	Ttll7	tubulin tyrosine ligase-like family, member 7
17480078	-1.59	0.0421	Picalm	phosphatidylinositol binding clathrin assembly protein
17425533	1.67	0.0421	Epb41l4b	erythrocyte membrane protein band 4.1 like 4b
17324762	-1.34	0.0421	Rnf168	ring finger protein 168
17288616	1.74	0.0421	Tppp	tubulin polymerization promoting protein
17368658	1.6	0.0423	Ppp1r26	protein phosphatase 1, regulatory subunit 26
17443663	1.25	0.0423	Tsc22d4	TSC22 domain family, member 4
17290078	1.91	0.0424	Gm6416	predicted gene 6416
17270874	1.71	0.0424	Scn4a	sodium channel, voltage-gated, type IV, alpha
17548475	1.34	0.0424		
17490312	1.29	0.0424	Nr1h2	nuclear receptor subfamily 1, group H, member 2
17266107	1.32	0.0425	Abr	active BCR-related gene
17451816	-1.4	0.0427	Hspb8	heat shock protein 8
17359464	1.28	0.0427	Frat1	frequently rearranged in advanced T cell lymphomas
17475026	1.41	0.0429	Ethe1	ethylmalonic encephalopathy 1
17377989	-1.31	0.0429	Mapre1	microtubule-associated protein, RP/EB family, member 1
17350656	-1.29	0.0429	Csnk1g3	casein kinase 1, gamma 3
17468933	1.51	0.0429	Iqsec1	IQ motif and Sec7 domain 1
17498115	1.36	0.043	Prr33	proline rich 33
17539879	-1.31	0.043	Plp2	proteolipid protein 2
17489464	-1.57	0.0431	Scn1b	sodium channel, voltage-gated, type I, beta
17287866	1.33	0.0431	1700066J03Rik	RIKEN cDNA 1700066J03 gene
17285097	1.4	0.0431	Dip2c	DIP2 disco-interacting protein 2 homolog C (Drosophila)
17535767	-1.5	0.0431	Rpl10	ribosomal protein L10
17531591	1.33	0.0431	Nbeal2	neurobeachin-like 2
17217967	-1.26	0.0431	Gm26048	predicted gene, 26048 [Source:MGI Symbol;Acc:MGI:5455825]
17337152	-1.97	0.0431		
17258341	1.33	0.0432	Llgl2	lethal giant larvae homolog 2 (Drosophila)
17416110	-1.27	0.0432	Mier1	mesoderm induction early response 1 homolog (Xenopus laevis
17455507	-1.32	0.0432	Hsph1	heat shock 105kDa/110kDa protein 1
17310808	-1.58	0.0433	Dap	death-associated protein
17510667	-1.52	0.0433	Tmem184c	transmembrane protein 184C
17266185	-1.33	0.0433	Nsrp1	nuclear speckle regulatory protein 1
17475311	1.3	0.0433	Atp5sl	ATP5S-like
17437529	-1.36	0.0433	Stim2	stromal interaction molecule 2
17531080	1.34	0.0435	Rnf123	ring finger protein 123
17229658	-1.54	0.0437	Fcer1g	Fc receptor, IgE, high affinity I, gamma polypeptide
17446060	-1.75	0.0437	AI506816	expressed sequence AI506816
17370641	-1.29	0.0437	Epc2	enhancer of polycomb homolog 2 (Drosophila)
17398368	1.44	0.0437	4921511C10Rik	RIKEN cDNA 4921511C10 gene
17267601	-1.31	0.0439	Scpep1	serine carboxypeptidase 1
17361404	-1.63	0.044	Dpp3	dipeptidylpeptidase 3
17243122	1.32	0.0441	Timm13	translocase of inner mitochondrial membrane 13
17498791	1.34	0.0441	Map2k7	mitogen-activated protein kinase kinase 7
17282641	1.41	0.0442	Prox2	prospero homeobox 2
17330314	1.6	0.0443	Ndufb4	NADH dehydrogenase (ubiquinone) 1 beta subcomplex 4
17409328	-1.31	0.0444	Sars	seryl-aminoacyl-tRNA synthetase
17548869	-2.15	0.0444		
17495677	-1.33	0.0445	Eri2	exoribonuclease 2
17517404	-1.4	0.0446	Dnaja4	DnaJ (Hsp40) homolog, subfamily A, member 4
17297913	-1.31	0.0446	Arf4	ADP-ribosylation factor 4
17496063	1.36	0.045	Gtf3c1	general transcription factor III C 1
17480534	1.35	0.045	Gdpd5	glycerophosphodiester phosphodiesterase domain containing 5
17221768	-1.37	0.045	Smap1	small ArfGAP 1
17547711	-1.38	0.045	Gm21319	predicted gene, 21319
17266563	-1.65	0.0452	Wsb1	WD repeat and SOCS box-containing 1
17329640	-1.55	0.0452	Atp13a3	ATPase type 13A3
17399812	-1.55	0.0453	S100a6	S100 calcium binding protein A6 (calcyclin)
17471342	1.47	0.0453	Fkbp4	FK506 binding protein 4
17524648	1.42	0.0453	Cdc37	cell division cycle 37
17276714	-1.38	0.0453	Mpp5	membrane protein, palmitoylated 5 (MAGUK p55 subfamily member 5)
17471127	-1.57	0.0453	Cd9	CD9 antigen
17344124	1.48	0.0453	1110038B12Rik	RIKEN cDNA 1110038B12 gene [Source:MGI Symbol;Acc:MGI:1916013]
17487927	1.55	0.0453	Lipe	lipase, hormone sensitive
17289794	-1.99	0.0453	Plk2	polo-like kinase 2
17283291	1.41	0.0453	Gm2614	predicted gene 2614
17403150	-1.26	0.0456	Tspan5	tetraspanin 5
17408375	1.54	0.0456	Gm22341	predicted gene, 22341 [Source:MGI Symbol;Acc:MGI:5452118]
17371296	-2.22	0.0457	Xirp2	xin actin-binding repeat containing 2
17335262	1.38	0.0457	Zfp523	zinc finger protein 523
17212364	-1.58	0.0458	1500015O10Rik	RIKEN cDNA 1500015O10 gene
17307113	1.4	0.0458	Gja3	gap junction protein, alpha 3
17450690	-1.41	0.0458	Evi5; Gm42149	ecotropic viral integration site 5; predicted gene, 42149
17392690	1.33	0.0458	Acss1	acyl-CoA synthetase short-chain family member 1
17363683	-1.34	0.0459	Pum3	pumilio RNA-binding family member 3
17459365	-1.64	0.046	Igkv13-84	immunoglobulin kappa chain variable 13-84
17233294	-1.4	0.046	Nus1	nuclear undecaprenyl pyrophosphate synthase 1 homolog (S. cerevisiae)
17286998	1.27	0.0461	Rbm24	RNA binding motif protein 24
17473171	1.45	0.0461	Ndufa3	NADH dehydrogenase (ubiquinone) 1 alpha subcomplex, 3
17348604	-1.4	0.0463	Impact	impact, RWD domain protein
17475431	1.76	0.0463	Nlrp9a	NLR family, pyrin domain containing 9A
17409005	1.62	0.0465	Slc6a17	solute carrier family 6 (neurotransmitter transporter), member 17
17475295	1.47	0.0465	4732471J01Rik	RIKEN cDNA 4732471J01 gene
17456584	-1.42	0.0465	Calu	calumenin
17310673	1.3	0.0467	Ank	progressive ankylosis
17376608	1.69	0.0468	1110034G24Rik	RIKEN cDNA 1110034G24 gene
17326166	-1.29	0.0468	Cd47	CD47 antigen (Rh-related antigen, integrin-associated signal transducer)
17509083	-1.28	0.0468	Fam149a	family with sequence similarity 149, member A
17260357	1.47	0.0468	H2afv	H2A histone family, member V
17412395	-1.45	0.0468	Bach2	BTB and CNC homology 2
17454398	-1.36	0.0468	Cox19	cytochrome c oxidase assembly protein 19
17447289	-1.31	0.0469	Nop14	NOP14 nucleolar protein
17212461	-1.68	0.0469	Col3a1	collagen, type III, alpha 1
17331078	-1.9	0.0471	Tmem45a	transmembrane protein 45a
17503300	1.39	0.0471	Hook2	hook homolog 2 (Drosophila)
17315960	-1.29	0.0471	Brix1	BRX1, biogenesis of ribosomes, homolog (S. cerevisiae)
17393483	1.4	0.0471	Nfs1	nitrogen fixation gene 1 (S. cerevisiae)
17396593	-1.33	0.0473	Sec62	SEC62 homolog (S. cerevisiae)
17352347	-1.4	0.0473	Tmx3	thioredoxin-related transmembrane protein 3
17468501	1.39	0.0474	2310040G24Rik	RIKEN cDNA 2310040G24 gene
17338174	1.28	0.0474	Pex6	peroxisomal biogenesis factor 6
17266322	1.33	0.0474	Eral1	Era (G-protein)-like 1 (E. coli)
17213428	-1.67	0.0474	Cyp20a1	cytochrome P450, family 20, subfamily a, polypeptide 1
17353639	-1.27	0.0474	Dnajc18	DnaJ (Hsp40) homolog, subfamily C, member 18
17504444	-1.24	0.0475	Cmtm3	CKLF-like MARVEL transmembrane domain containing 3
17497693	1.44	0.0475	Sirt3	sirtuin 3
17510749	-1.44	0.0475	Abce1	ATP-binding cassette, sub-family E (OABP), member 1
17308800	1.43	0.0475	Dnajc15	DnaJ (Hsp40) homolog, subfamily C, member 15
17287579	1.39	0.0477	Zfp346	zinc finger protein 346
17391212	-1.23	0.048	Ncaph	non-SMC condensin I complex, subunit H
17445089	1.38	0.048	Fry	furry homolog (Drosophila)
17395039	1.35	0.0481	Gm22773	predicted gene, 22773 [Source:MGI Symbol;Acc:MGI:5452550]
17300519	1.33	0.0481	Dcaf11	DDB1 and CUL4 associated factor 11
17527735	1.43	0.0481	Neo1	neogenin
17541856	-1.33	0.0482	Mtap7d3	MAP7 domain containing 3
17441595	1.47	0.0482	Tbx3	T-box 3
17402558	-1.36	0.0482	Elovl6	ELOVL family member 6, elongation of long chain fatty acids (yeast)
17501170	1.39	0.0482	Asb5	ankyrin repeat and SOCs box-containing 5
17261730	-1.42	0.0482	Rars	arginyl-tRNA synthetase
17446836	1.28	0.0482	Mpv17	MpV17 mitochondrial inner membrane protein
17464850	-1.38	0.0482	Thsd7a	thrombospondin, type I, domain containing 7A
17281142	-1.34	0.0482	Cfl2	cofilin 2, muscle
17361304	1.38	0.0482	Lrfn4	leucine rich repeat and fibronectin type III domain containing 4

17324332	-1.54	0.0482	Fetub	fetuin beta
17398180	-1.51	0.0482	Mfsd1	major facilitator superfamily domain containing 1
17445860	-1.72	0.0485	Napepld	N-acyl phosphatidylethanolamine phospholipase D
17466530	3.49	0.0485	Rny1	RNA, Y1 small cytoplasmic, Ro-associated
17368476	-2.15	0.0485	Adamtsl2	ADAMTS-like 2
17457913	1.8	0.0486	Clcn1	chloride channel, voltage-sensitive 1
17548965	-1.51	0.0486	Gm2666; Csprs	predicted gene 2666; component of Sp100-rs
17334495	1.28	0.0489	Nme3; Mrps34	NME/NM23 nucleoside diphosphate kinase 3; mitochondrial ribosomal protein S34
17344126	-2.47	0.0489	Hspa1b; Hspa1a	heat shock protein 1B; heat shock protein 1A
17438189	-1.47	0.049	Rasl11b	RAS-like, family 11, member B
17508325	-1.9	0.049	Htra4	HtrA serine peptidase 4
17481723	-1.32	0.0491	Snora23	small nucleolar RNA, H/ACA box 23
17240840	-1.36	0.0491	Mcm9	minichromosome maintenance complex component 9
17428659	-1.38	0.0491	Nasp	nuclear autoantigenic sperm protein (histone-binding)
17446643	1.27	0.0492	Hadha	hydroxyacyl-Coenzyme A dehydrogenase/3-ketoacyl-Coenzyme A thiolase/enoyl-Coenzyme A hydratase (trifunctional protein), alpha subunit
17347353	-1.53	0.0493	Gm6548	eukaryotic translation elongation factor 1 alpha 1 pseudogene
17262855	-1.48	0.0493	Tnip1	TNFAIP3 interacting protein 1
17392366	1.48	0.0494	Banf2os	barrier to autointegration factor 2, opposite strand
17229891	1.53	0.0494	Atp1a2	ATPase, Na+/K+ transporting, alpha 2 polypeptide
17403463	1.54	0.0496	Syde2	synapse defective 1, Rho GTPase, homolog 2 (C. elegans)
17364948	1.39	0.0496	Slc25a28	solute carrier family 25, member 28
17324623	-2.08	0.0496	Hes1	hairy and enhancer of split 1 (Drosophila)
17549822	-1.39	0.0496		
17277697	1.29	0.0496	Adck1	aarF domain containing kinase 1
17497414	1.39	0.0496	Mapk1ip1	mitogen-activated protein kinase 1 interacting protein 1
17468172	1.28	0.0497	Dguok	deoxyguanosine kinase
17539125	-1.5	0.0498	Rps6ka3	ribosomal protein S6 kinase polypeptide 3
17263220	-1.36	0.0499	2310058D17Rik	RIKEN cDNA 2310058D17 gene [Source:MGI Symbol;Acc:MGI:1922944]
