# Supplementary figures and images for: Cpxm2 as a novel candidate for cardiac hypertrophy and failure in hypertension
Source: Hypertens Res. 2021 Dec 16;45(2):292–307. doi: 10.1038/s41440-021-00826-8 (PMC8766285; doi:10.1038/s41440-021-00826-8)

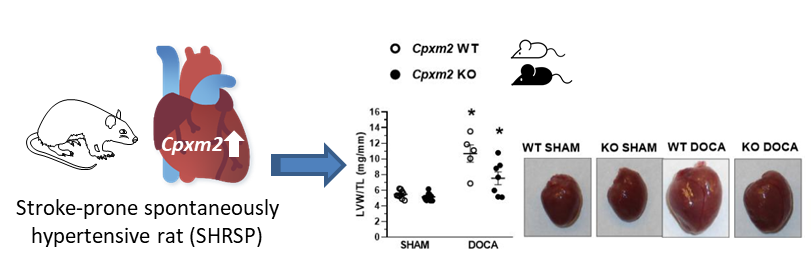

Supplement: Supplementary file 6 — Supplementary image [file 41440_2021_826_MOESM6_ESM.tif]
